# New scaling relations to compute atom-in-material polarizabilities and dispersion coefficients: part 2. Linear-scaling computational algorithms and parallelization†

**DOI:** 10.1039/c9ra01983a

**Published:** 2019-10-17

**Authors:** Thomas A. Manz, Taoyi Chen

**Affiliations:** Department of Chemical & Materials Engineering, New Mexico State University Las Cruces New Mexico 88003-8001 USA tmanz@nmsu.edu

## Abstract

We present two algorithms to compute system-specific polarizabilities and dispersion coefficients such that required memory and computational time scale linearly with increasing number of atoms in the unit cell for large systems. The first algorithm computes the atom-in-material (AIM) static polarizability tensors, force-field polarizabilities, and *C*_6_, *C*_8_, *C*_9_, *C*_10_ dispersion coefficients using the MCLF method. The second algorithm computes the AIM polarizability tensors and *C*_6_ coefficients using the TS-SCS method. Linear-scaling computational cost is achieved using a dipole interaction cutoff length function combined with iterative methods that avoid large dense matrix multiplies and large matrix inversions. For MCLF, Richardson extrapolation of the screening increments is used. For TS-SCS, a failproof conjugate residual (FCR) algorithm is introduced that solves any linear equation system having Hermitian coefficients matrix. These algorithms have mathematically provable stable convergence that resists round-off errors. We parallelized these methods to provide rapid computation on multi-core computers. Excellent parallelization efficiencies were obtained, and adding parallel processors does not significantly increase memory requirements. This enables system-specific polarizabilities and dispersion coefficients to be readily computed for materials containing millions of atoms in the unit cell. The largest example studied herein is an ice crystal containing >2 million atoms in the unit cell. For this material, the FCR algorithm solved a linear equation system containing >6 million rows, 7.57 billion interacting atom pairs, 45.4 billion stored non-negligible matrix components used in each large matrix-vector multiplication, and ∼19 million unknowns per frequency point (>300 million total unknowns).

## Abstract 2

Linear-scaling algorithms were developed to compute atom-in-material polarizabilities and dispersion coefficients using MCLF and TS-SCS methods.

## Graphical abstract

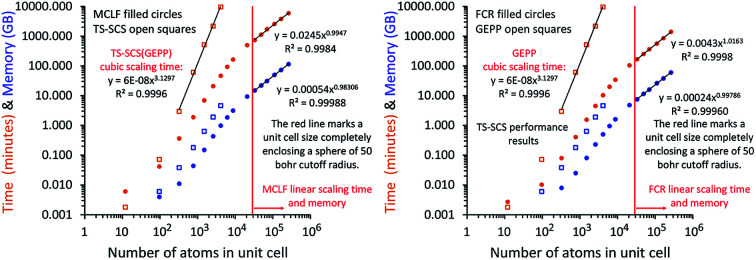

## 1. Introduction

In the first part of this series, we introduced the MCLF method (acronym from authors' last initials) to compute atom-in-material (AIM) polarizabilities and dispersion coefficients.^1^ We compared chemical performance of MCLF to the Tkatchenko–Scheffler method with self-consistent screening (TS-SCS).^1^ Computed polarizabilities and/or dispersion coefficients were compared to experimental and/or high-level computational benchmark data for isolated atoms, diatomic molecules, small polyatomic molecules, fullerenes, polyacenes, and solids.^1^ For HIV reverse transcriptase complexed with an inhibitor, computed MCLF and TS-SCS AIM polarizabilities and dispersion coefficients were compared to the OPLS biomolecular force-field.^1^

In this article, we introduce computationally efficient algorithms that extend the MCLF and TS-SCS methods to materials having large numbers of atoms in the unit cell. For sufficiently large systems, both the required memory and computational time scale linearly with increasing number of atoms in the unit cell. Our methods can easily be applied to materials containing millions of atoms in the unit cell. This is orders of magnitude larger than unit cells for materials previously studied with MCLF and TS-SCS methods. For small unit cells, our methods are still faster and require less memory than direct matrix inversion with negligible difference in computational precision.

The TS-SCS method was introduced in 2012.^2^ TS-SCS can be combined with multibody dispersion (MBD), a damping function, and density functional theory (DFT) to give a DFT + dispersion method.^2^ Ambrosetti *et al.* introduced range-separated dipole interaction tensors to avoid (or minimize) double-counting dispersion interactions in the combined MBD@rsSCS framework.^3^ The TS-SCS method requires atom-in-material 〈*r*^3^〉 radial moments as inputs.^2,4^ Several TS-SCS variants used different partitioning methods to compute the 〈*r*^3^〉 moments: TS-SCS/Hirshfeld,^2,4,5^ TS-SCS/iterative Hirshfeld,^6,7^ and TS-SCS/DDEC6.^1^ Normally, the TS method assumes a constant polarizability to 〈*r*^3^〉 moment ratio for a specific chemical element irrespective of its charge states in a material.^2,4^ Recently, Gould *et al.* introduced a Fractionally Ionic (FI) method that adjusts the polarizability to 〈*r*^3^〉 moment ratio using partial atomic charges.^8^ This requires a library of reference ion polarizabilities and *C*_6_ coefficients for free atoms in different charge states.^8,9^ However, these reference polarizabilities and *C*_6_ coefficients are hard to compute for anions such as O^2−^ that contain unbound electrons^10^ in their free state.

In the TS-SCS method, the *C*_6,*A*_ dispersion coefficient is computed using the Casimir–Polder integral (eqn (8)), which requires AIM polarizabilities as a function of imaginary frequency (aka imfreq). These AIM polarizabilities are computed by solving the following linear equation system1
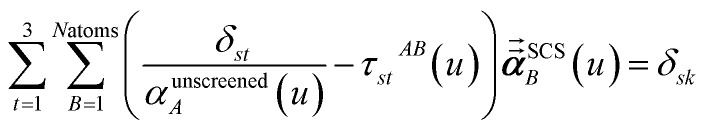
at each imfreq point *u*, where *τ*_*st*_^*AB*^(*u*) is the dipole–dipole interaction tensor, *s* and *t* are spatial indices (*e.g.*, *x*, *y*, or *z*), *δ*_*st*_ is the Kronecker delta, *α*^unscreened^_*A*_(*u*) is the isotropic AIM unscreened polarizability as a function of *u*, and 
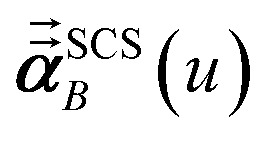
 is the self-consistent screened AIM polarizability tensor as a function of *u*.^2^ A dipole interaction cutoff length function turns off dipole interactions between atoms separated by a distance larger than this cutoff length (*e.g.*, 50 bohr). This makes the coefficients matrix sparse when the material's unit cell is much larger than this cutoff length. Because the dipole interaction tensor is symmetric in both atomic indices and spatial indices, developing a computationally efficient TS-SCS algorithm is functionally equivalent to developing a computationally efficient algorithm to solve a set of real-valued symmetric sparse linear equations.

At its heart, therefore, this is a fundamental linear algebra problem. Specifically, to solve a linear equation system **M***y* = ***W*** having a sparse real-valued symmetric coefficient matrix **M**. A naïve approach would invert the matrix **M** to compute *y* = (inverse(**M**))***W***. However, this is computationally infeasible for matrices containing a large number of rows (*N*rows). First, direct matrix inversion algorithms (*e.g.*, Gaussian elimination with partial pivoting (GEPP) and the more advanced Strassen algorithm) have computational costs scaling between (*N*rows)^2^ and (*N*rows)^3^.^11^ Second, inverse(**M**) may be dense (or at least considerably less sparse than **M**) leading to high matrix multiplication costs. The cost of multiplying two dense square matrices is between (*N*rows)^2^ and (*N*rows)^3^,^11^ and multiplying a dense square matrix times a dense vector would cost (*N*rows)^2^ if using conventional multiplication. Therefore, the primary goal is to solve this linear equation system without any large dense matrix multiplies or large matrix inversions. Here, a new conjugate residual algorithm is introduced to achieve this with a computational cost proportional to *N*rows. This new conjugate residual algorithm resists round-off errors.

As described in the first article of this series,^1^ the MCLF method includes numerous important innovations. It uses a conduction limit upper bound to ensure assigned AIM polarizabilities do not exceed those of a perfect conductor. It uses *m*-scaling to smoothly transition between the scaling behaviors of surface and buried atoms. It uses new scaling relationships to describe changes in the polarizability-to-〈*r*^3^〉 moment ratio as a function of the atomic charge state without requiring quantum mechanical (QM) computed reference polarizabilities and *C*_6_ values for charged atoms. This is an important advantage, because some isolated charged atoms are unstable (*e.g.*, O^2−^ as discussed above). To compute several different kinds of AIM polarizabilities, MCLF separates directional from non-directional dipole interaction tensor contributions. This includes: (a) non-directionally screened polarizabilities to be used as force-field input parameters, (b) fluctuating polarizabilities to compute the *C*_6_ dispersion coefficients, and (c) static polarizabilities containing long-range dipole alignment due to a constant externally applied electric field. A new polarizability partition and iterative polarizability screening ensure assigned AIM polarizabilities are non-negative. A proof that MCLF *α*^force-field^, *α*^non-dir^(*u*), *α*^low_freq^, and *α*^screened^(*u*) are ≥0 is provided in the ESI.† MCLF uses a multibody screening function to capture the fluctuating dipole alignment at short distances and disorder at long distances. This leads to more accurate *C*_6_ coefficients. Quantum Drude oscillator (QDO) parameters yield higher-order AIM dispersion coefficients (*e.g.*, *C*_8_, *C*_9_, *C*_10_) and associated mixing rules.

The MCLF method includes polarizability screening using a much different approach than TS-SCS.^1^ While TS-SCS solves a linear equation system (eqn (1)), MCLF uses an incremental polarizability screening.^1^ Accordingly, our linear-scaling MCLF algorithm is vastly different in mathematical approach than our linear-scaling TS-SCS algorithm. As explained in Section 2 below, we use Richardson extrapolation of the screening increments to achieve high computational efficiency and precision for MCLF. Both our linear-scaling MCLF and linear-scaling TS-SCS algorithms avoid large dense matrix multiplications and large matrix inversions.

These computational methods should find widespread use beyond the specific applications studied herein. This new conjugate residual algorithm solves any linear equation system having Hermitian coefficients matrix. (Conditioning is required for ill-conditioned matrices.) Because it has mathematically provable stable convergence that resists round-off errors, this conjugate residual algorithm should find widespread applications to solve a plethora of scientific computing problems involving large sparse linear equation systems. Potential applications for such a method are vast.

The remainder of this article is organized as follows. Section 2 presents the linear-scaling MCLF algorithm. The failsafe conjugate residual and linear-scaling TS-SCS algorithms are introduced in Section 3. Section 4 contains the performance results: required computational times, required memory, and parallelization efficiencies. All computational timing and memory results reported in this paper are for the MCLF or TS-SCS analysis; they do not include the prior quantum chemistry calculation (if any) and prior atomic population analysis used to provide the input data. Section 5 concludes. The ESI† contains mathematical derivations and proofs pertaining to these methods.

Finally, we comment on the choice of atomic population analysis method (APAM) used to provide input data to the MCLF and TS-SCS methods. The memory requirements for MCLF and TS-SCS analysis do not depend on which APAM provides the input data. The required computational time for MCLF analysis does not depend on which APAM provides the input data. The required computational time for TS-SCS analysis using the FCR algorithm only depends on the number of FCR iterations, which could potentially be weakly affected by the choice of APAM used to provide the input data. In this article, we used DDEC6 (ref. 12 and 13) atomic population analysis to generate the input data for MCLF and TS-SCS analysis. (Other stockholder partitioning methods could potentially be used.) Currently, DDEC6 is the most recent generation of the Density Derived Electrostatic and Chemical (DDEC) methods that are optimized to quantify important electrostatic, magnetic, and chemical properties across diverse materials.^12–18^ All DDEC6 calculations were performed using the Chargemol program.^12^

The overall sequence is: QM calculation → APAM → MCLF or TS-SCS analysis. DDEC6 and many other stockholder partitioning methods can be made strictly linear-scaling by using a cutoff radius around each atom in a material.^12^ The electron and spin densities assigned to an atom in the material are zero outside this cutoff radius. This yields linear-scaling computational time and memory, because the number of integration points per atom does not increase as *N*atoms increases.^12^*N*atoms is the number of atoms in the unit cell.

QM computation of the material's electron and spin densities is the rate-limiting step in this overall sequence. Significant progress has been made developing linear-scaling DFT codes that have applications to studying unit cells containing ∼10 000 atoms.^62–69^ Orbital-free DFT calculation (which is presently most suitable for treating metallic conductors^66^) was performed for a million atom system.^66^ Further improvements are needed to enable DFT calculations on millions and billions of atoms. Even without DFT calculations of such large systems, the MCLF (or TS-SCS) method could still find applications to materials containing millions of atoms. For example, by using DFT calculations and DDEC6 analysis on smaller clusters to parameterize information for atom types, followed by MCLF (or TS-SCS) analysis for the full material. This strategy would make sense, because DDEC6 analysis is more localized (*i.e.*, cutoff radius = 5 Å) compared to MCLF (or TS-SCS) analysis (*e.g.*, dipole interaction cutoff length = 50 bohr).^1,13^

## 2. Linear-scaling MCLF algorithm

### 2.1 How strict linear-scaling is achieved

Strict linear-scaling means that each and every part of the MCLF program scales no worse than linear in computational time and memory as *N*atoms increases when *N*atoms is sufficiently large. First, no allocatable arrays having multiple dimensions of size proportional to *N*atoms (*e.g.*, *My_array*(*N*atoms, *N*atoms)) are allocated for *N*atoms greater than a threshold. Second, no nested DO loops having two indices of ranges proportional to *N*atoms (*e.g.*, requiring *Order*(*N*atoms^2^) iterations) are executed for *N*atoms greater than a threshold. This is achieved by making sure the program is written such that all sets of nested DO loops and allocatable arrays (that are operational for *N*atoms greater than a threshold) have at most one index whose range is proportional to *N*atoms.

This is physically enabled by using a dipole interaction cutoff length such that any two atoms farther apart have no direct interactions. As explained in Section 2.2 below, the program constructs lists of interacting atom pairs and uses these in subsequent calculations. Because each atom in the material only directly interacts with a limited number of other atoms, for sufficiently large *N*atoms the total number of symmetry unique interacting atom pairs is proportional to *N*atoms. This is true even if the material is periodic and extends forever. This is conceptually equivalent to a sparse matrix algebra.

Other mathematical innovations are also employed. First, a special lookup table method (see Section 2.4) is used to compute the total dispersion coefficient2
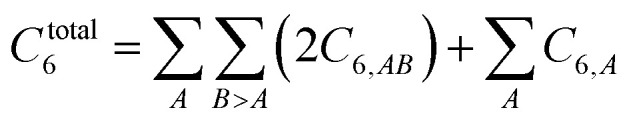
without having to directly itemize this summation over all pairs of atoms in the unit cell. Second, directly inverting a large matrix (even if it is sparse) can potentially require more operations than *Order*(*N*rows). As explained in Section 2.5, a linear-scaling algorithm was developed that avoids large matrix inversion. This algorithm is linear-scaling, because it requires a fixed number of large matrix-vector multiplications in which the matrix is sparse. This matrix is the dipole interaction tensor, whose non-zero components correspond to the interacting atom pairs. Hence, each large matrix-vector multiplication corresponds to looping over the lists of symmetry unique interacting atom pairs.

As explained in Section 2.6, all of these linear-scaling innovations were parallelized to enable fast multi-core computing. Moreover, all of these innovations were programmed in a general way that handles 0, 1, 2, or 3 periodic boundary conditions (PBC). For input files of materials having periodic unit cells, these MCLF and TS-SCS programs include the option to use or ignore the input file PBC. For example, a molecule can be simulated in a planewave code (such as VASP) by placing it near the center of a large cube with PBC. In this case, to yield AIM polarizabilities and dispersion coefficients for the isolated molecule, the MCLF or TS-SCS program should be set to ignore these PBC; this turns off all interactions between periodic images.

### 2.2 Lists of interacting atom pairs

We use a capital letter (*e.g.*, *A*, *B*) to represent an atom in the reference unit cell. A small letter represents a translated atomic image that is not necessarily located in the reference unit cell. For example, *b* = (*B*, *L*_1_, *L*_2_, *L*_3_) represents the image of atom *B* that is translated *L*_1_ times the first lattice vector ***v⃑***_1_, *L*_2_ times the second lattice vector ***v⃑***_2_, and *L*_3_ times the third lattice vector ***v⃑***_3_.

3 ***R⃑***_*b*_ = ***R⃑***_*B*_ + *L*_1_***v⃑***_1_ + *L*_2_***v⃑***_2_ + *L*_3_***v⃑***_3_

is the nuclear position of this image, where ***R⃑***_*B*_ is the nuclear position of the parent atom in the reference unit cell.

During MCLF analysis, two separate lists of interacting atom pairs are prepared after the unscreened calculation and before the screened calculation. For convenience, we refer to these as the ‘small’ and ‘large’ lists, where ‘small’ and ‘large’ refer to the interaction cutoff distance. Without loss of generality, the first atom in each pair can be considered to reside within the reference unit cell. The second atom is located somewhere within the dipole interaction cutoff length of the first atom. Only translation symmetry unique atom pairs need to be included in the loops over atom pairs. As explained in the earlier bond order article, an atom image pair is translation symmetry unique if and only if at least one of the following criteria is satisfied: (i) the index numbers of the atoms are different (*e.g.*, atom 210 and atom 1056), (ii) *L*_1_ > 0, (iii) *L*_1_ = 0 and *L*_2_ > 0, or (iv) *L*_1_ = *L*_2_ = 0 and *L*_3_ > 0.^18^

The small lists all atom pairs having ‘overlapping’ Gaussian dipole model densities as defined by the cutoff criterion4
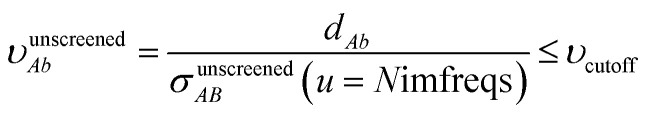
where

5 *d*_*Ab*_ = ‖***R⃑***_*A*_ − ***R⃑***_*b*_‖

is the distance from atom *A* to image *b*, and *σ*^unscreened^_*AB*_(*u* = *N*imfreqs) is the static attenuation length. This criterion ensures erfc(*υ*_*Ab*_) ≈ exp(−(*υ*_*Ab*_^2^)) ≈ 0 for any atom pair not included in the list. The value *υ*_cutoff_ = 5^4/3^ was chosen such that even if the (partially) screened polarizability is larger than the unscreened polarizability by up to a factor of five, then erfc(*υ*^screened^_*Ab*_) ≤ erfc(5) = 1.5 × 10^−12^ and exp(−(*υ*^screened^_*Ab*_)^2^) ≤ exp(−25) = 1.4 × 10^−11^. The following information was saved in the small list array for each included atom pair: the index numbers of the first and second atoms, the translation integers for the second atom, the atomic number of each atom, *d*_*Ab*_, the value of the smooth cutoff function, the value of the multibody screening function times the smooth cutoff function, and the following six tensor components: *η*_1,1_ = 3(Δ*x*)^2^/*d*_*Ab*_^2^ − 1, *η*_1,2_ = *η*_2,1_ = 3Δ*x*Δ*y*/*d*_*Ab*_^2^, *η*_1,3_ = *η*_3,1_ = 3Δ*x*Δ*z*/*d*_*Ab*_^2^, *η*_22_ = 3(Δ*y*)^2^/*d*_*Ab*_^2^ − 1, *η*_2,3_ = *η*_3,2_ = 3Δ*y*Δ*z*/*d*_*Ab*_^2^, *η*_3,3_ = 3(Δ*z*)^2^/*d*_*Ab*_^2^ − 1. Here, Δ*x*, Δ*y*, and Δ*z* are the Cartesian components of ***R⃑***_*A*_ − ***R⃑***_*b*_.

The large lists all atom pairs (*A*, *B*) in the reference unit cell for which atom *A* (first atom) is located a distance less than dipole interaction cutoff length to at least one image of atom *B* (second atom). The self-pair (*A*, *A*) is included if and only if one of the non-trivially translated images of atom *A* is located a distance less than dipole interaction cutoff length to atom *A* in the reference unit cell. The number of atom pairs in the large list is always ≤*N*atoms(*N*atoms + 1)/2. For example, a NaCl crystal containing two atoms in the reference unit cell would contain three pairs in the large list: Na–Na, Na–Cl, and Cl–Cl. For each pair (*A*, *B*) in the large list, a loop is performed over all atom *b* images located within dipole interaction cutoff length of atom *A*, and the following sums are accumulated and stored:6
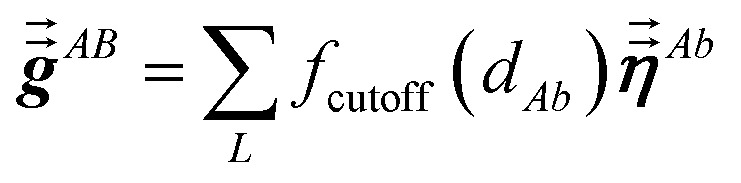
7
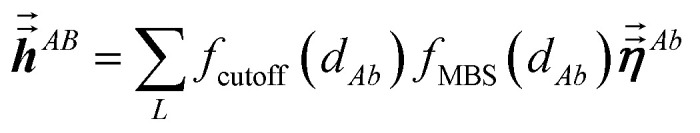
Here, *f*_cutoff_ and *f*_MBS_ are the smooth cutoff function and multi-body screening function, respectively, described in the prior article.^1^ Since 
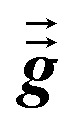
 and 
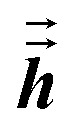
 are symmetric with respect to exchange of the spatial indices, only six components need to be computed and stored for each.

Our algorithm for constructing these small and large lists has time and memory requirements scaling linearly with increasing *N*atoms. Section S4 of the ESI† explains this algorithm's details. Fig. S1 of the ESI† is a flow diagram summarizing this process. The ESI† also contains Fortran modules that can be examined for coding details. Data is grouped to enable fast computation by avoiding all array searches. For example, information is ordered such that arrays do not have to be searched to identify which atoms belong in each spatial region. Also, each array allocation is performed once, rather than continuously appending arrays (which would be extremely slow). This is accomplished by first performing a ‘dry run’ code block that executes a sequence to count up the required array size, followed by array allocation, followed by a code block that writes data to the allocated array. The four key steps to construct these lists are:

1. *Define basis vectors and unit cell parallelepiped:* A parallelepiped of non-zero volume is constructed to enclose the system's unit cell. Three basis vectors correspond to this parallelepiped's non-collinear edges. For a periodic direction, the basis vector is the corresponding periodic lattice vector. For a non-periodic direction, the basis vector is chosen to be of a non-zero length that fully encloses all the nuclear positions. Periodic basis vectors can be non-perpendicular to each other (*e.g.*, triclinic unit cells), but each non-periodic basis vector is chosen to be perpendicular to the other basis vectors.

2. *Divide the unit cell parallelepiped into spatial regions:* This unit cell parallelepiped is divided into a whole number of spatial regions along each basis vector. A periodic direction produces an infinite number of periodic images of each region, while a non-periodic direction has only the reference image. Atoms in the reference unit cell are classified by region, and a sorted list is prepared such that atoms of the same region are adjacent in this sorted list. Because each spatial region is defined such that its volume is less than that of a sphere of dipole interaction cutoff length radius, the number of atoms in each region is always below a threshold. The code ignores regions that do not contain any atoms. Because empty regions are skipped, having a few atoms in the center of an enormous unit cell would execute quickly.

3. *Construct arrays listing interacting region pair images:* Two spatial region images interact if the minimum distance between inter-region points is ≤the dipole interaction cutoff length. Because the spatial regions and their images are coordinate system indexed, a list of interacting region pair images is constructed without having to construct a double summation over all region pairs. Thus, even for an extremely large unit cell (*e.g.*, containing billions of atoms) divided into many regions (*e.g.*, millions), the list of interacting region pair images is constructed in time and memory scaling linearly with increasing unit cell size. Different regions can interact with different periodic images. For example, in an extremely large unit cell, a region near the center would interact only with nearby regions in the reference unit cell, while a region not too far from the left edge would interact with some regions in the reference unit cell and some other region images in the left-translated unit cell. Thus, a first array is constructed listing pairs of regions having any interacting images, and a second array is prepared that lists which specific images of each particular region pair interact. Region pairs that do not interact are not included in these two arrays.

4. *Construct two lists of interacting atom pairs:* Because interacting atom pairs must be contained in interacting region pair images, the code identifies the interacting atom pairs by executing an outer loop over the interacting region pair images and inner loops over the atoms in these regions (along with tests for inclusion criteria). Using the list of atoms sorted by region makes this process cache access friendly. For each such atom pair, tests are performed to determine if it meets the small and large list inclusion criteria. If so, its information is added to the small and/or large lists. Because the number of interacting region pair images scales linearly with large *N*atoms and the number of atoms in each region is below a threshold, these small and large lists are constructed in linear-scaling computational time and memory for large *N*atoms.

Having separate small and large lists provides the following computational efficiencies during the subsequent MCLF polarizability screening. First, non-directional screening only needs to be performed over the ‘overlapping’ atoms contained in the small list. Second, the computationally expensive erfc function only needs to be evaluated for atom pairs in the small list. This provides computational savings during directional screening that first loops over all pairs in the small list and then over all pairs in the large list. Third, the large list contains pre-computed sums over all periodic images of interacting atom pairs. For non-overlapping atoms, this avoids re-computing any sums over periodic images at each screening increment and each frequency point.

These two lists of interacting atom pairs are used as follows. For each screening increment of each frequency point, non-directional screening loops over all atom pairs in the small list to compile the necessary dipole–dipole interaction terms. For each screening increment of each frequency point, directional screening first loops over all atom pairs in the small list and then over all atom pairs in the large list to compile the associated dipole–dipole interaction terms. More details are provided in Sections 2.5 and 2.6 below.

Table 1 studies the effect of dipole interaction cutoff length on computed precision. Graphene was chosen as a test system, because it has strong long-range dipole–dipole coupling. As shown in Table 1, *α*^static^ was the most sensitive to the dipole interaction cutoff length, and *α*^force-field^ was the least sensitive. For 2-dimensional sheets such as graphene, doubling the dipole interaction cutoff length increases the computational cost by approximately four-fold. For dense materials with large unit cells, doubling the dipole interaction cutoff length increases the computational cost by approximately eight-fold. We selected dipole interaction cutoff length = 50 bohr as a good compromise between computational cost and precision. This value was used for all other results in this article.

**Table 1:** Effect of dipole interaction cutoff length on the MCLF computed properties of graphene. Results (in atomic units) are per carbon atom

	10 bohr	25 bohr	50 bohr	75 bohr	100 bohr
*C*_6_	37.91	50.33	**53.36**	53.80	53.90
*α* ^static^	11.42	18.13	**22.73**	24.82	26.01
*α* ^low_freq^	9.75	11.81	**12.28**	12.35	12.36
*α* ^force-field^	7.03	7.03	**7.03**	7.03	7.03

The graphene primitive unit cell was optimized in VASP^19–21^ using the PBE^22^ functional, a 400 eV planewave cutoff, and the projector augmented wave (PAW^23,24^) method. The *k*-point mesh and grid spacing followed previous recommendations.^12^ This yielded a nearest neighbor C–C distance of 1.42 Å. Our MCLF and TS-SCS calculations of graphene were performed using 2-D not 3-D periodic boundary conditions (*i.e.*, the spacing between graphene layers was set to infinite at the start of MCLF or TS-SCS calculation).

### 2.3 Integration over imaginary frequencies

The Casimir–Polder integral relates the *C*_6,*AB*_ dispersion coefficient between two subsystems *A* and *B* to the product of their fluctuating polarizabilities at imfreq 
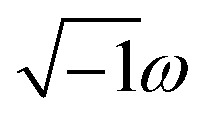
 integrated over all imfreqs:^25^8
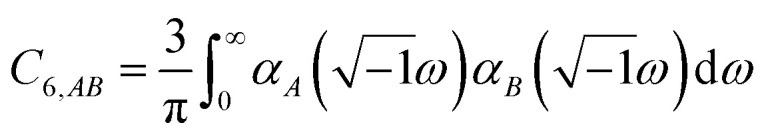


As shown in eqn (8), the integration limits are zero to infinity. For convenience, we used the substitution of variables9
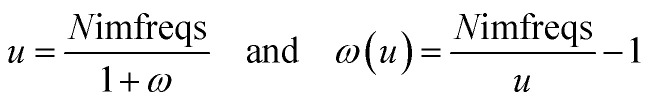
to make both integration limits finite. Differentiating eqn (9) yields10
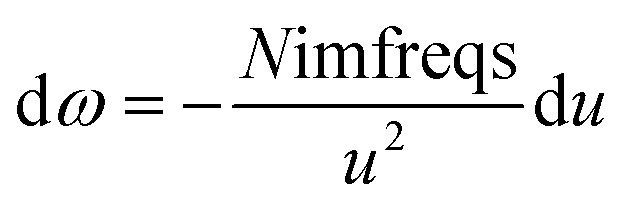
which upon substitution into eqn (8) for *A* = *B* gives11
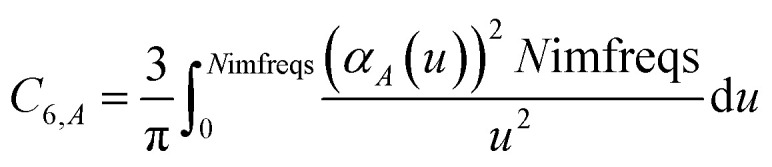
*u* = 0 corresponds to infinite imfreq. Near infinite imfreq, the polarizability becomes inversely proportional to *ω*^2^.^26^ This is incorporated into the TS-SCS^2,4^ and MCLF^1^ methods using a Padé approximation^27^ for the dynamic unscreened polarizability:12
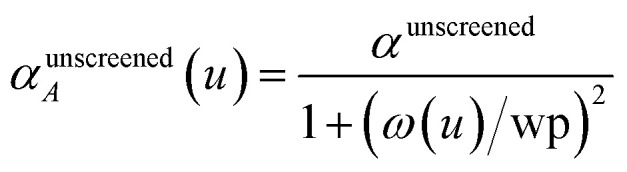
At this limit, the integrand in eqn (11) simplifies to13
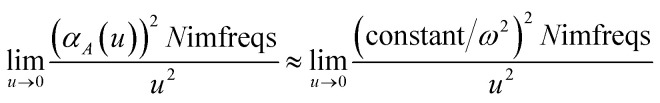
Substituting eqn (9) into (13) gives14
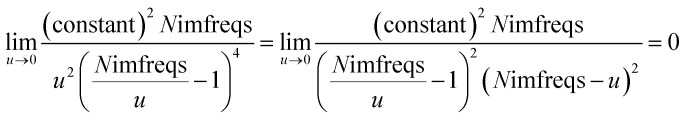
Therefore, the *u* = 0 point contributes nothing to the integral.

We numerically integrated using Richardson extrapolation (*i.e.*, Romberg integration).^28–30^ Dividing the (0, 1) interval into 2^*G*^ segments and performing Romberg integration of order (*G*, *G*) yields an integration error of the order 2^−*G*(2*G*+2)^.^28^ Normally, Romberg integration of 2^*G*^ segments corresponds to 2^*G*^ + 1 integration points. Since the *u* = 0 point contributes nothing to the integral, this leaves only *N*imfreqs = 2^*G*^ nontrivial integration points. The ESI† contains the Romberg integration weights *c*^Romberg^_*G*,*u*_ of these 2^*G*^ integration points for *G* = 1 to 5. The integral is computed using the following sum:15
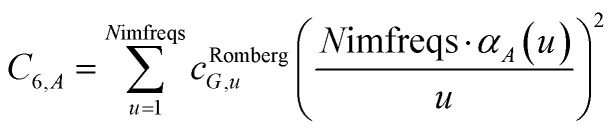
where16
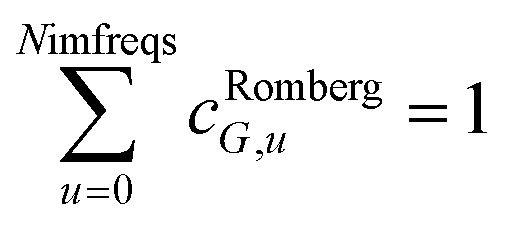


Table 2 shows computed AIM *C*_6_ coefficients for four materials. We used the same geometries and electron densities for K solid, NaF solid, and C_60_ fullerene as in the companion article.^1^ The K bcc solid showed a small difference (0.3%) in the *C*_6_ coefficient between 16 and 32 integration points. All other results were virtually identical for 16 and 32 integration points. Moreover, all results for 8 integration points differed by <1% from the higher integration points. This shows the results are highly converged for 16 integration points, which is the value we chose.

**Table 2:** Effect of Romberg integration order (*G*) and number of integration points (*N*imfreqs = 2^*G*^) on the computed atom-in-material *C*_6_ coefficients in atomic units

*G* →	3	4	5
*N*imfreqs →	8	**16**	32
Graphene	53.33	**53.36**	53.36
K bcc solid	444.29	**448.35**	447.00
NaF solid	11.69 (Na)	**11.69 (Na)**	11.69 (Na)
	45.13 (F)	**45.26 (F)**	45.26 (F)
C_60_	29.57	**29.67**	29.67

### 2.4 Lookup table for computing *C*_6_ = sum(*C*_6,*AB*_)

Although the mixed *C*_6,*AB*_ dispersion coefficients could in principle be computed from the Casimir–Polder integral using *α*_*A*_(*u*) and *α*_*B*_(*u*), this would involve many integrations for unit cells containing thousands or more atoms. Therefore, we used the following mixing formula which is consistent with both Padé approximation^27^ and QDO^31^ models:17
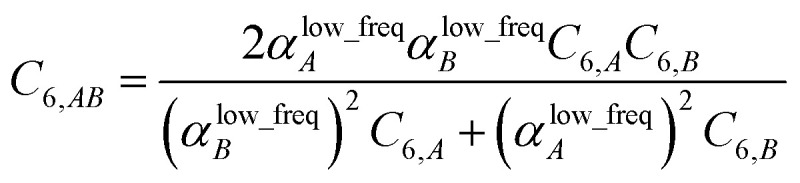


In our method, the polarizabilities appearing in eqn (17) must be *α*^low_freq^, because these are the polarizabilities associated with the dispersion interaction. Of note, the TS and TS-SCS methods use similar mixing formula, except the polarizabilities appearing in the mixing formula are *α*^TS^ and *α*^TS-SCS^,^2,4^ because those methods do not yield *α*^low_freq^.

When using the mixing rule of eqn (17), itemized computation of the *C*_6_ dispersion coefficient per unit cell requires a total of (*N*atoms)(*N*atoms − 1)/2 individual *C*_6,*AB*_ evaluations. This itemized approach to computing *C*^total^_6_ would be computationally expensive for unit cells containing a billion or more atoms. Atomistic (*e.g.*, molecular dynamics or Monte Carlo) simulations employing an interaction cutoff distance require only a small subset of {*C*_6,*AB*_} for large unit cells, because atoms outside the cutoff distance do not interact. Therefore, it would be extremely wasteful to compute and print all the individual *C*_6,*AB*_ values for large unit cells. It is preferred to compute and print {*α*^low_freq^_*A*_} and {*C*_6,*A*_} that can be inserted into eqn (17) to generate selected *C*_6,*AB*_ whenever needed. For a billion atoms, storing {*α*^low_freq^_*A*_} and {*C*_6,*A*_} requires only 16 gigabytes (GB), while storing {*C*_6,*AB*_} would require 4 exabytes. Therefore, it is unnecessary for the program to compute, store, or print the full {*C*_6,*AB*_}.

A linear-scaling algorithm to compute *C*^total^_6_ is now introduced to address this issue. Defining18
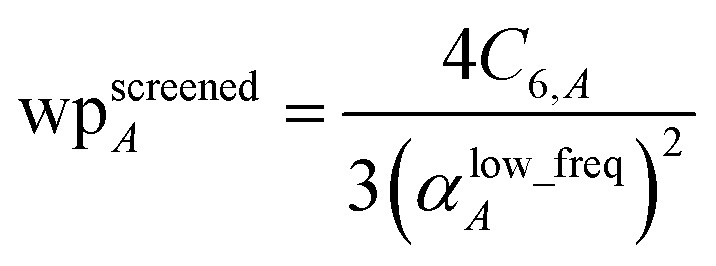
allows eqn (17) to be re-written as the following variant of the London formula19
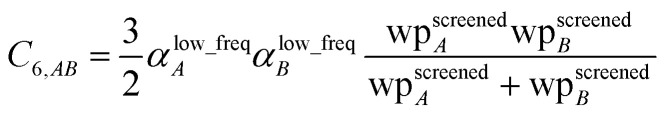
and analogously for the unscreened values20
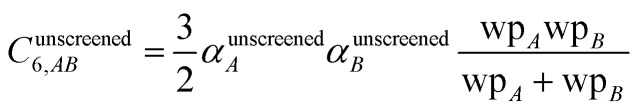


Linear-scaling computation is achieved by constructing a lookup table that uniformly spaces ln(wp_values):

21 *x*_1_ = ln(min(wp_values)) − 10^−2^

22 *x*_Num_lookup_ = ln(max(wp_values)) + 10^−2^

23
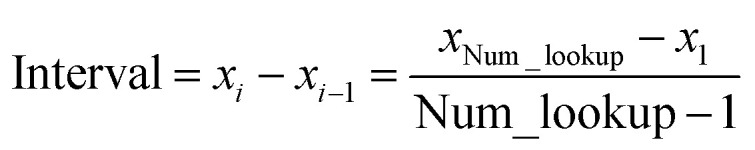

24 wp_table_*i*_ = exp(*x*_*i*_)

The ±10^−2^ in eqn (21) and (22) ensures the interval always remains non-zero even if max(wp_values) = min(wp_values), and it also ensures the floor operation in eqn (25) will not produce an index *j* < 1 or > Num_lookup even in the presence of fixed precision floating point round-off. An array alpha_table having Num_lookup components is initialized to zero. A loop is then performed over all atoms in the unit cell. Each atom *A*'s polarizability *α*_*A*_ contributes to two adjacent array values:25
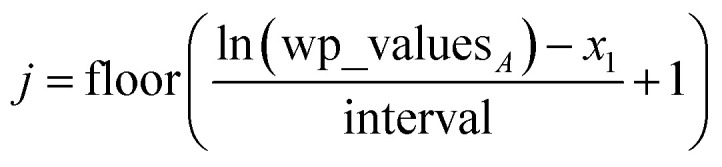
26
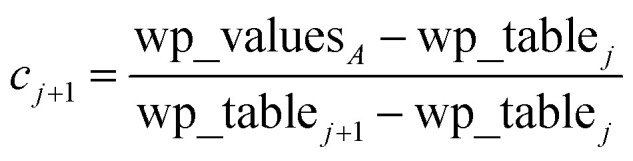

27 *c*_*j*_ = 1 − *c*_*j*+1_

28 alpha_table_*j*_ ← alpha_table_*j*_ + *c*_*j*_*α*_*A*_

29 alpha_table_*j*+1_ ← alpha_table_*j*+1_ + *c*_*j*+1_*α*_*A*_

After this loop completes, *C*^total^_6_ is computed by the following double summation (that involves an outer loop over lookup table entries and an inner loop over atoms in the unit cell):30
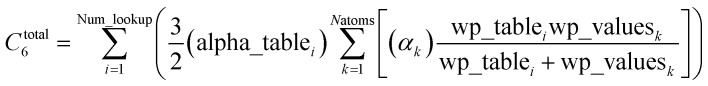
The code skips the inner loop over atoms whenever alpha_table_*i*_ = 0.

Since each atom contributes to exactly two alpha_table components (eqn (28) and (29)), the total number of loop iterations performed is always ≤min(2(*N*atoms^2^), (*N*atoms × Num_lookup)) (eqn (30)) + *N*atoms (eqn (25)–(29)) + Num_lookup (eqn (24)). For *N*atoms > Num_lookup this method exhibits linear-scaling computational cost, because as *N*atoms increases only the size of the inner loop in eqn (30) increases linearly while the outer loop over lookup table entries has fixed size. For systems containing a small number of symmetry distinct atoms, each symmetry identical atom contributes to the same two alpha_table components, so the number of non-zero alpha_table components never exceeds twice the number of symmetry distinct atoms, which results in super acceleration for computations on large supercells comprised of many symmetry equivalent primitive cells (*e.g.*, see graphene and ice in Table 3).

**Table 3:** Accuracy of wp lookup table computation of *C*^unscreened^_6_ and *C*^screened^_6_ compared to directly itemized computation. Num_lookup = 10^5^. Unsigned relative errors (UREs) are listed in parts per trillion (ppt = 10^−12^). Computational times (in seconds) for serial execution on the Comet cluster are listed. The column labeled PBC is the number of periodic boundary conditions. *N*atoms is the number of atoms per unit cell

Material	PBC	*N*atoms	Unscreened			Screened		
			URE (ppt)	Itemized (s)	Lookup (s)	URE (ppt)	Itemized (s)	Lookup (s)
C_60_	0	60	0.002	0.000	0.005	0.002	0.000	0.005
C_50_H_24_	0	74	0.02	0.000	0.005	0.1	0.000	0.005
B-DNA	3	733	13.6	0.003	0.012	14.5	0.003	0.012
KUCDIW	3	1104	0.7	0.008	0.006	0.9	0.007	0.006
Graphene	2	2	0.001	0.000	0.005	0.003	0.000	0.005
Graphene	2	20 000	1.2	2.6	0.007	0.6	2.6	0.006
Ice	3	12	0.5	0.000	0.005	0.7	0.000	0.005
Ice	3	165 888	0.4	177	0.30	0.7	176	0.27
Ice	3	263 424	0.5	447	0.87	0.7	444	0.77
Ice	3	1 053 696	0.3	7859	3.7	0.6	7929	3.4
Ice	3	2 107 392	1.4	31 368	7.5	4.0	31 719	6.7

The ESI† contains a Fortran file that implements this algorithm (eqn (21)–(30)) as a function *C*^total^_6_(alpha_values, wp_values, *N*atoms, Num_lookup). This function parallelizes eqn (30) using OpenMP. Using appropriate arguments, this same function can be called to compute either *C*^unscreened^_6_ or *C*^screened^_6_.

This method has astonishingly high precision. As proved in Section S3 of the ESI,† the unsigned relative error (URE) in *C*^total^_6_ is always less than interval^2^/16, which is ≤∼4 × Num_lookup^−2^. Thus, with Num_lookup = 10^5^, the URE is usually ≤∼4 × 10^−10^. Table 3 confirms this high computational precision for several example materials.

All real variables were 64-bit, except *C*^total^_6_ (both eqn (2) and (30)), alpha_table, and a temporary variable to accumulate the inner sum of eqn (30)) were 128-bit reals. The need for a 128-bit real (aka ‘quadruple precision’) for the *C*^total^_6_ variable is straightforward to demonstrate. For a unit cell containing one billion similar atoms, *C*^total^_6_ ≈ 10^18^*C*_6,*A*_. Since 64-bit reals have approximately 15 significant base-ten digits, attempting to accumulate *C*^total^_6_ in the traditional manner by adding *C*_6,*AB*_ one-at-a-time would cause the accumulation to stall at *C*^total^_6_ ≈ 10^15^*C*_6,*A*_ which would be off by a factor of ∼10^3^. For a unit cell containing one million atoms, accumulating *C*^total^_6_ one atom pair at a time using a 64-bit real number will not stall, but the final working capacity will be reduced to ∼3 precision digits: 15 (digits for 64-bit reals) − 12 digits (for *C*^total^_6_/*C*_6,*A*_ ≈ 10^12^) = ∼3 remaining digits of precision for individual *C*_6,*AB*_ addition. 128-Bit reals have approximately 34 significant base-ten digits, which is adequate. Although using 128-bit reals for alpha_table and the temporary variable are not required, this helps preserve quadruple precision during *C*^total^_6_ computation.

Computational times in Table 3 refer to the time required to call and compute the *C*^total^_6_ function (*i.e.*, eqn (2) and (17) or (21)–(30)) and do not include times required to compute function inputs. As shown in Table 3, the wp lookup table computation can sometimes be slower than directly itemized computation when *N*atoms is small but is always faster when *N*atoms is large. Therefore, the program is normally configured to perform directly itemized computation of *C*^total^_6_ when *N*atoms < threshold_wp_lookup, and wp lookup table computation otherwise. For all calculations in this paper except those listed in Table 3, we set threshold_wp_lookup = 2 × Num_lookup. When *N*atoms > ∼2 × Num_lookup, the lookup table algorithm always requires fewer iterations than directly itemized computation.

This same wp lookup table method for computing *C*^total^_6_ is also employed in the TS-SCS(FCR) method described in Section 3 below. The same Num_lookup = 10^5^ and threshold_wp_lookup = 2 × Num_lookup are used there. The same function described above is used, but called with the alpha_values and wp_values appropriate for computing *C*^TS^_6_ or *C*^TS-SCS^_6_ for the TS-SCS(FCR) method.

### 2.5 Avoiding direct inversion of large matrices

Direct inversion of large matrices is extremely computationally expensive. Gaussian elimination with partial pivoting (GEPP) is a common matrix inversion algorithm that exhibits numerical instability for some matrices but is usually stable in practice.^32,33^ GEPP, QR factorization, Cholesky and LDL decomposition (for positive definite Hermitian matrices), and other common matrix inversion algorithms have computational costs scaling proportional to the number of rows (*N*rows) cubed.^11,33^ The more complicated Strassen algorithm has scaling proportional to *N*rows^log(7)/log(2)^ = *N*rows^2.807…^.^11^

A direct matrix inversion algorithm would first construct the dipole interaction tensor, then invert it to get the multibody polarizability matrix, and then contract the multibody polarizability matrix to get the AIM polarizability tensors.^34^ Consider a material containing 1 million atoms in the unit cell. For this material, the dipole interaction tensor has 3 million rows and an equal number of columns. The computational cost of the matrix inversion step would be on the order of *N*rows^3^ = (3 × 10^6^)^3^ = ∼2.7 × 10^19^ floating point operations (*i.e.*, ∼27 exaflop). This corresponds to ∼7500 computational hours on a teraflop computer or ∼7.5 computational hours on a petaflop computer for each imfreq point. Since the dipole interaction tensor would need to be inverted at several (*e.g.*, 16) imfreq points, a TS-SCS or MCLF calculation on this material would be computationally prohibitive when using direct matrix inversion. Moreover, storing the full multi-body polarizability matrix in double-precision arithmetic would take (8 bytes per double precision real) × (3 × 10^6^ rows) × (3 × 10^6^ columns) = 7.2 × 10^13^ bytes = 72 terabytes of random access memory (RAM). This is a huge amount of memory.

To address this problem, we developed a new computational algorithm that converges to the same solution without requiring any matrix inversions. This inverse-free algorithm is conceptually related to the iterative Schulz method for matrix inversion. In the Schulz method, an estimate **P**^(*i*−1)^ for the inverse of matrix **Q** is iteratively refined by^35^

31 **P**^(*i*)^ = 2**P**^(*i*−1)^ − **P**^(*i*−1)^**QP**^(*i*−1)^

where index *i* is the Schulz iteration. The key difference between our inverse-free algorithm and the Schulz matrix inversion method is that we exploit the particular structures of the dipole interaction tensor and multi-body polarizability matrix contraction to enable us to work with *N*atoms scalars for the non-directional screening and *N*atoms 3 × 3 matrices for the directional screening instead of working with *N*atoms × *N*atoms and 3*N*atoms × 3*N*atoms matrices for matrix inversions using GEPP or Schulz method. This allows us to reduce the computational cost from cubic scaling (for GEPP or Schulz method using conventional matrix multiplications) to linear in *N*atoms as the unit cell becomes sufficiently large.

Substituting **Q**_*j*+1_ = **D**_*j*_ + *Δ*_*j*_*τ*_*j*_ (see companion article^1^ for specific definitions of **D**_*j*_ and *τ*_*j*_) into (31) with *i* = 1 gives the first Schulz iteration as

32 **P**_*j*+1_^(1)^ = 2**P**^(0)^_*j*+1_ − **P**^(0)^_*j*+1_(**D**_*j*_ + *Δ*_*j*_*τ*_*j*_)**P**^(0)^_*j*+1_

where

33 **P**^(0)^_*j*+1_ = inv(**D**_*j*_)

is the initial estimate for inv(**Q**_*j*+1_). The subscript *j* + 1 is the screening iteration and should not be confused with the Schulz iteration (superscript *i*). *Δ*_*j*_ is the screening increment. Substituting eqn (33) into (32) and simplifying yields

34 **P**_*j*+1_^(1)^ = inv(**D**_*j*_) − inv(**D**_*j*_)(*Δ*_*j*_*τ*_*j*_)inv(**D**_*j*_)

Note that: (1) *τ*_*j*_ includes non-zero blocks only for interacting atom pairs (*i.e.*, atom pairs in the ‘small’ and/or ‘large’ lists), (2) **D**_*j*_ and inv(**D**_*j*_) are block-diagonal matrices, (3) the non-zero blocks of inv(**D**_*j*_) are the partially screened atomic polarizabilities {*α*_*j*_^*A*^} (which are scalar for non-directional screening and tensor for fluctuating and static screening), and (4) the multi-body polarizability matrix **P** is only needed in its contracted form as partially screened atomic polarizabilities {*α*_*j*_^*A*^}.

Three different types of atomic polarizabilities are computed using this inverse-free algorithm. As explained in the companion article,^1^ atoms with larger pre-screened polarizability get a proportionally larger piece of the screening mixed polarizability contribution:35
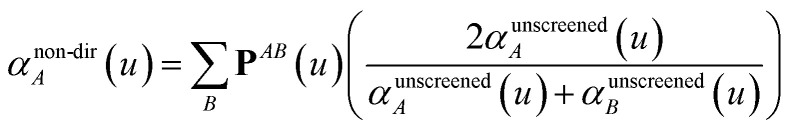
36
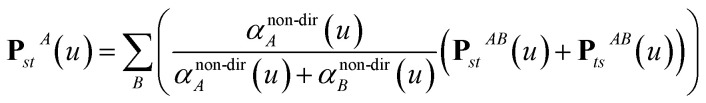
37
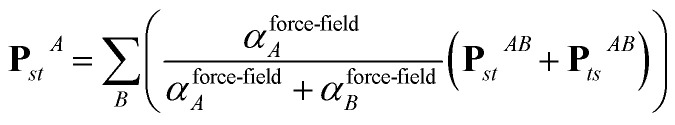
where *s* and *t* represent spatial directions ∈{*x*, *y*, *z*}. Eqn (35), (36), and (37) correspond to the non-directional, fluctuating, and static polarizabilities, respectively. Using these to simplify eqn (34) yields38
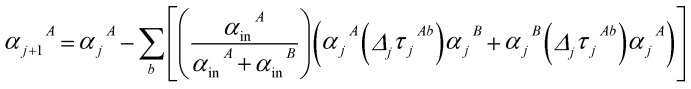


For non-directional screening to compute *α*^non-dir^_*A*_(*u*), eqn (38) becomes39
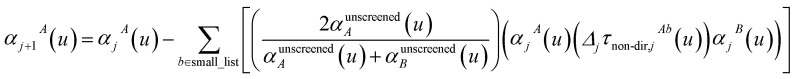
*τ*_non-dir,*j*_^*Ab*^(*u*) is defined in the companion article,^1^ where the attenuation length for the pair of atoms *A* and *B*40
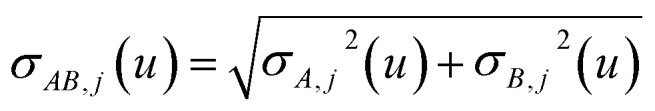
is updated at the start of screening iteration *j* + 1 using *α*_*j*_^*A*^(*u*) and *α*_*j*_^*B*^(*u*) to compute the spherical Gaussian dipole width41
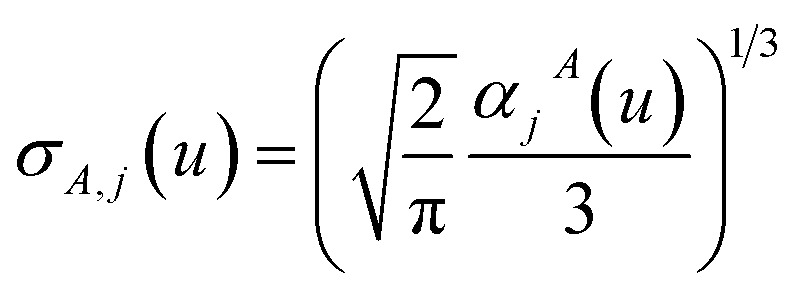
The process starts with

42 *α*_*j*=1_^*A*^(*u*) = *α*^unscreened^_*A*_(*u*)

and ends with *α*^non-dir^_*A*_(*u*) as the value on the left-side of eqn (39) after the last screening increment finishes.

For directional screening to compute *α*^screened^_*A*_(*u*), eqn (38) becomes43
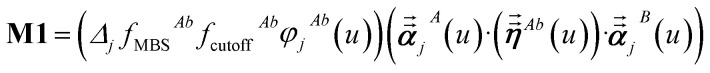
44
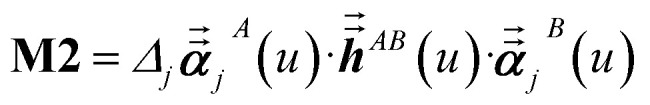
45
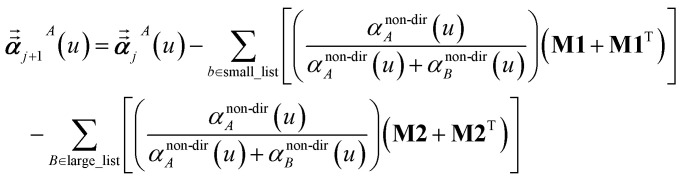
where **M1** and **M2** are square matrices with 3 rows and46
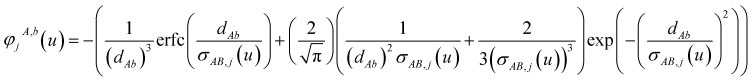
47
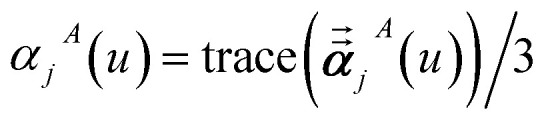


In eqn (46), the attenuation length *σ*_*AB*,*j*_(*u*) is updated at the start of screening iteration *j* + 1 using *α*_*j*_^*A*^(*u*) and *α*_*j*_^*B*^(*u*) from eqn (47) inserted into eqn (40) and (41). The process starts with48
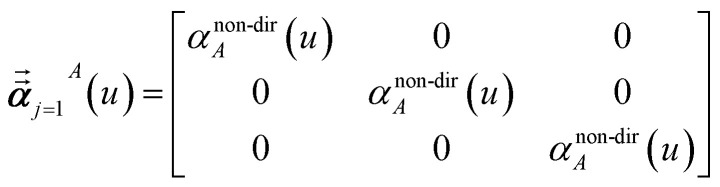
and ends with *α*^screened^_*A*_(*u*) as one-third the trace of the tensor on the left-side of eqn (45) after the last screening increment finishes.

For directional screening to compute 
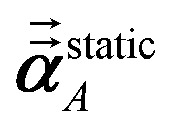
, eqn (38) becomes49
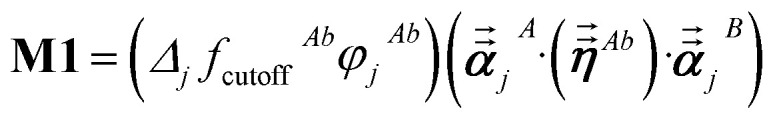
50
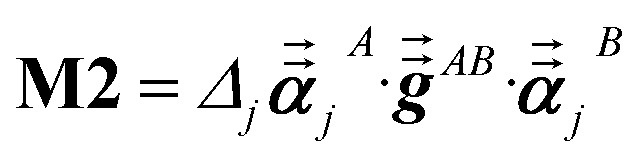
51
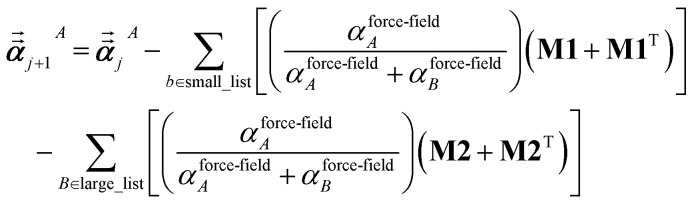
where *φ*_*j*_^*Ab*^ and *α*_*j*_^*A*^ are defined analogous to eqn (46) and (47) except based on 
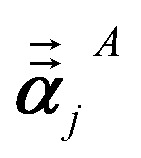
 from eqn (51). The attenuation length *σ*_*AB*,*j*_(*u* = *N*imfreqs) is updated at the start of screening iteration *j* + 1 using *α*_*j*_^*A*^ and *α*_*j*_^*B*^ inserted into eqn (40) and (41). The process starts using eqn (48) and ends with 
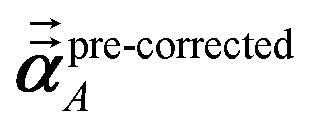
 as the tensor on the left-side of eqn (51) after the last screening increment finishes. As explained in the companion article,^1^ the following anisotropic polarizability correction is then applied to get the static polarizability tensors52
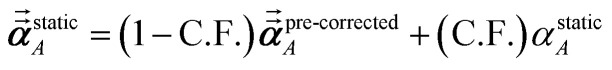
53
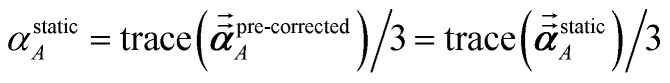
using a correction factor (C.F.) = 0.2. Summing 
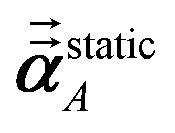
 over all atoms in the unit cell gives the unit cell (or molecular) polarizability tensor. Taking one-third the trace of the AIM or molecular static polarizability tensors yields the corresponding isotropic static polarizabilities.

The order of the error residual is now derived. Since **P**_*j*+1_^(1)^ is a calculated estimate of inv(**Q**_*j*+1_), we begin by defining the error residual as

54 Residual = ‖**I** − **Q**_*j*+1_**P**_*j*+1_^(1)^‖

where ‖‖ is any desired matrix norm and **I** is the identity matrix. Multiplying eqn (34) by **Q**_*j*+1_ = **D**_*j*_ + *Δ*_*j*_*τ*_*j*_ gives

55 **Q**_*j*+1_**P**_*j*+1_^(1)^ = **I** − *Δ*_*j*_*τ*_*j*_inv(**D**_*j*_)(*Δ*_*j*_*τ*_*j*_)inv(**D**_*j*_)

Multiplying each side of eqn (55) by −1, adding **I**, and taking the norm gives

56 ‖**I** − **Q**_*j*+1_**P**_*j*+1_^(1)^‖ = ‖*Δ*_*j*_*τ*_*j*_inv(**D**_*j*_)*Δ*_*j*_*τ*_*j*_inv(**D**_*j*_)‖ = (*Δ*_*j*_)^2^‖*τ*_*j*_inv(**D**_*j*_)*τ*_*j*_inv(**D**_*j*_)‖

The right-most side follows from the theorem that norm(scalar × matrix) = scalar × norm(matrix).^36^ For convenience, we used fixed size screening increments: *Δ*_*j*_ = *Δ*. Eqn (56) shows that at each screening iteration, the difference between this inverse-free algorithm and direct matrix inversion will be on the order of *Δ*^2^, where *Δ* is the screening increment. Since the total number of screening iterations is 1/*Δ*, the total difference between this inverse-free algorithm and direct matrix inversion will be on the order of *Δ*.

By taking the limit *Δ* → 0, the inverse-free and direct matrix inversion algorithms converge to the identical solution. We used Richardson extrapolation^30,37^ to evaluate the limit *Δ* → 0. As explained in the prior paragraph, without Richardson extrapolation the overall error using screening increment *Δ* is of *Order*(*Δ*). Each Richardson extrapolation step removes a successive power of *Δ* in the error. After *K* Richardson extrapolation steps (RES), the remaining error will thus be of *Order*(*Δ*^*K*+1^). We extrapolated using screening increments of 1, 2^−1^, 2^−2^, …2^−*K*^. During each such screening process, the screening increments sum to 157
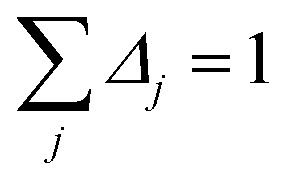
Therefore, this extrapolation corresponds to extrapolating from results computed with 1, 2, 2^2^, …2^*K*^ screening points. The final *Δ* = 2^−*K*^ undergoes *K* RES leading to a residual error of *Order*(2^−*K*(*K*+1)^). Note that 7 RES is approximately twice as expensive as 6 RES and four times as expensive as 5 RES.

Richardson extrapolation was applied to the following atomic polarizabilities:

Non-directional screening:58
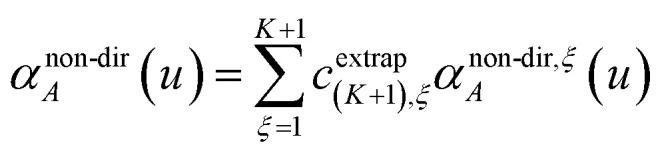


Frequency-dependent directional screening:59
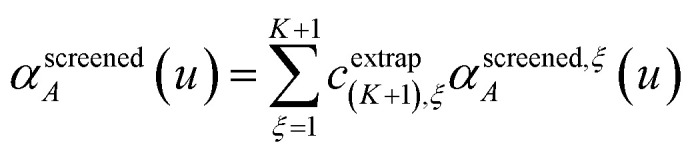


Static induced directional screening:60
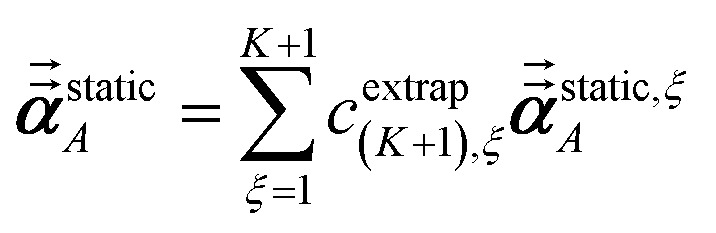
As explained above, *α*^force-field,*ξ*^_*A*_, *α*^screened,*ξ*^_*A*_(*u*), 
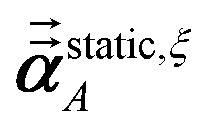
 are the values computed using 2^*ξ*−1^ screening points (*i.e.*, screening increment of 2^1−*ξ*^). The coefficients *c*^extrap^_(*K*+1),*ξ*_ for *K* = 1 to 7 RES are given in the ESI.† Note that

61 *α*^low_freq^ = *α*^screened^_*A*_(*u* = *N*imfreqs)

62 *α*^force-field^ = *α*^non-dir^_*A*_(*u* = *N*imfreqs)

Table 4 lists computed results for graphene. After testing, we settled on five RES for the force-field polarizabilities (*i.e.*, non-directional screening), five RES for the fluctuating polarizabilities used to compute *C*_6_ coefficients, and seven RES for the static polarizabilities. The static polarizability requires more RES than the force-field and fluctuating polarizabilities, because of the longer range dipole–dipole interactions contributing to the static polarizability. The inverse-free and GEPP algorithms converged to the same results in the limit *Δ* → 0. The inverse-free algorithm is preferable, because it exhibits better computational cost scaling than GEPP. As an additional test, the (5,7) RES calculation was repeated using a 20 000 atom supercell (which is a 100 × 100 replication of the 2 atom primitive unit cell) which shows the MCLF results for a large supercell are identical to those computed for a primitive unit cell. The 20 000 atom graphene supercell was constructed using DDEC6 AIM properties from the primitive cell; no DFT calculation on the large supercell was needed.

**Table 4:** Effect of the number of Richardson extrapolations steps (RES) applied to the MCLF screening increments. Graphene was chosen as a test system, because it has strong long-range dipole–dipole coupling. For (5,7) the results for a 20 000 atom supercell are shown in parentheses. Results are per atom

	(5,5)^a^ RES	(6,6)^a^ RES	(7,7)^a^ RES	(5,7)^a^ RES	(7,7)^a^ GEPP^b^
*α* ^static^	22.62	22.72	22.73	**22.73 (22.73)**	22.73
*α* ^low_freq^	12.28	12.28	12.28	**12.28 (12.28)**	12.28
*C*_6_	53.36	53.36	53.36	**53.36 (53.36)**	53.36
*α* ^force-field^	7.03	7.03	7.03	**7.03 (7.03)**	7.03
Relative computational cost	17	34	68	**20** c	Cubic

a The first number in parentheses refers to the number of RES used for non-directional screening and short-range directional screening to compute *α*^non-dir^_*A*_(*u*) and *α*^screened^_*A*_(*u*), respectively. The second number in parentheses is the number of RES used for long-range directional screening to compute 
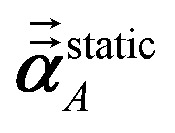
.

b Explicit large matrix construction and inversion using Gaussian elimination with partial pivoting.

c Nominal computational cost for (5,7) algorithm includes 16 frequency points at 5 RES plus the static polarizability at 7 RES, which gives 16(1) + 1(4) = 20 compared to 17 for the (5,5) algorithm.

### 2.6 Code design and parallelization

The MCLF code was parallelized using OpenMP. OpenMP divides the work among different computing cores on a single cache-coherent node.^38,39^ The Fortran code was parallelized by adding OpenMP compiler directives that parallelized the most computationally intense loops. OpenMP codes can be compiled in serial mode by simply instructing the compiler to ignore the OpenMP directives. We used several techniques for efficient OpenMP parallelization discussed in one of our previous articles.^12^ Specifically, the array indices were ordered in a cache friendly manner, large arrays were declared as shared variables to avoid large memory increases when adding more parallel threads, and when needed reductions were used with the *parallel* directive rather than with the *do schedule* directive (see Section 3.3 for example).^12^ Directives such as *single*, *atomic*, and *critical* that require one thread to wait on another should be kept to a minimum.^12,38,39^ To maximize parallelization efficiency, each thread should be given enough work such that the overhead time to set up the threads is a small percentage of the parallel region time.^12,38,39^ For example, when parallel work is done over the ‘small’ and ‘large’ atom pair lists (*e.g.*, Fig. 3 and 4), the parallel threads were created just prior to looping over the ‘small’ list and terminated just after looping over the ‘large’ list.

**Fig. 1:**
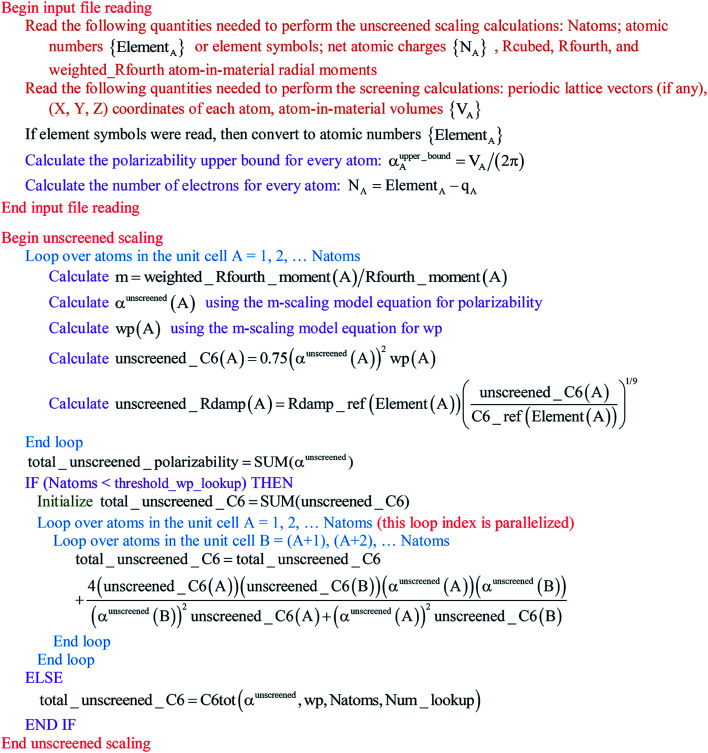
Pseudocode for the input file reading and unscreened calculation using *m*-scaling. The *m*-scaling model equations for polarizability and wp are given in the companion article.^1^

**Fig. 2:**
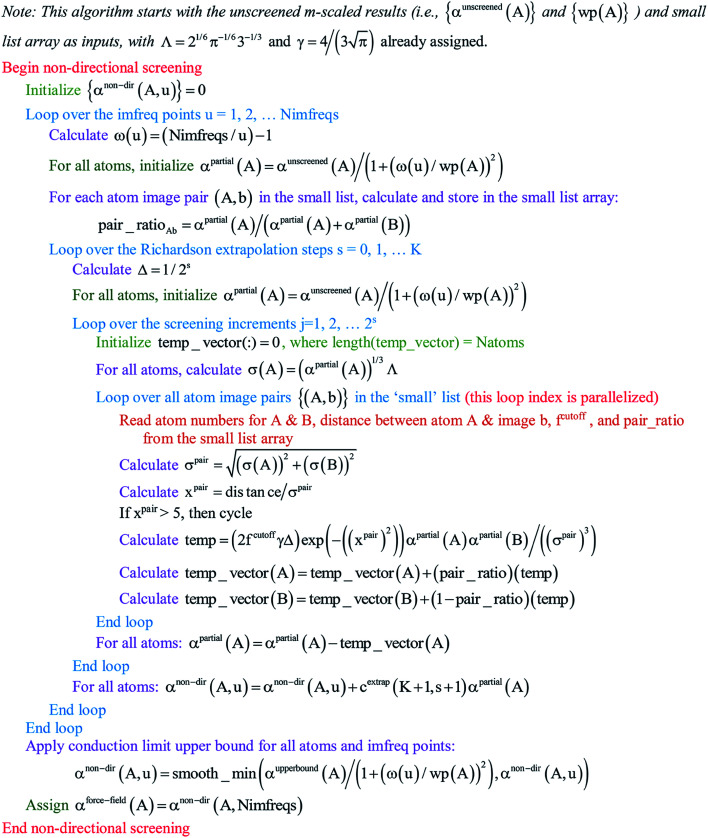
Pseudo-code for non-directional screening to compute {*α*^non-dir^_*A*_(*u*)} and the force-field polarizabilities {*α*^force-field^_*A*_} using inverse-free algorithm and Richardson extrapolation.

**Fig. 3:**
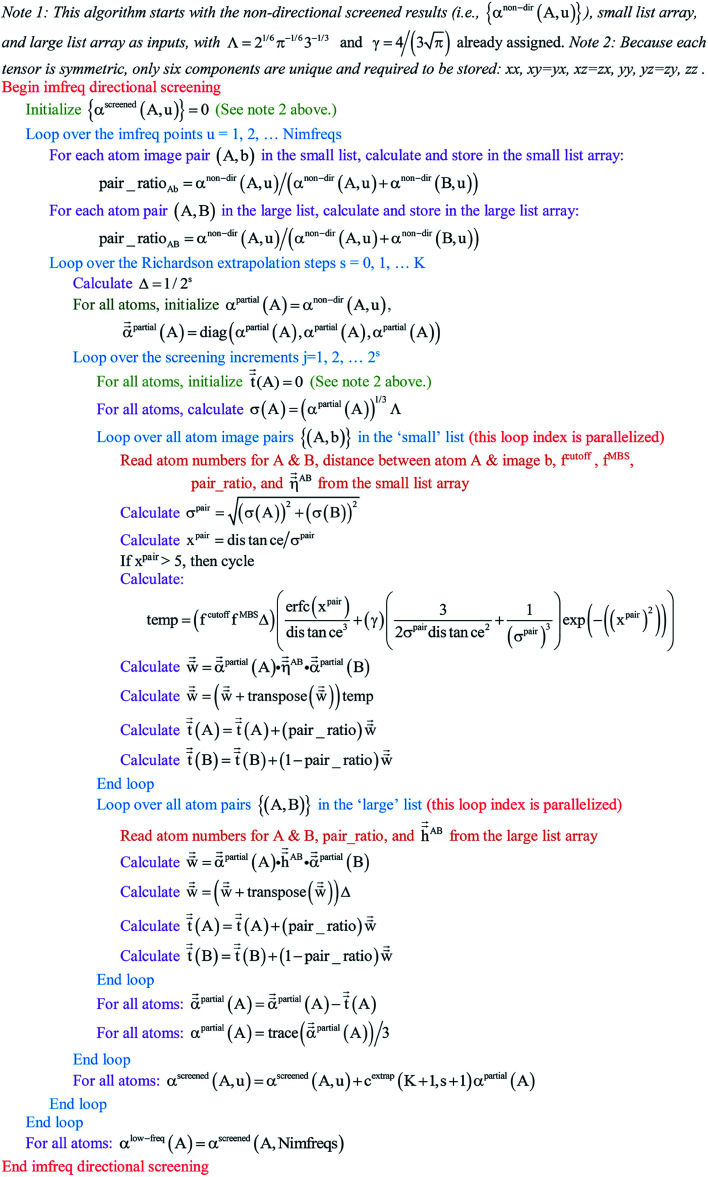
Pseudocode for imfreq directional screening to compute {*α*^screened^_*A*_(*u*)} and {*α*^low_freq^_*A*_} using inverse-free algorithm and Richardson extrapolation.

Paradoxically, the computer code to perform MCLF analysis is actually simpler than the mathematical equations that define MCLF analysis. The reason is that many array indices that appear in the mathematical equations are actually not required in the computer code, because these quantities can be computed in-place. For this reason, pseudocodes for the main parts of MCLF analysis are illustrated in this section. Fig. 1 illustrates the pseudocode for input file reading and unscreened calculation using *m*-scaling. Fig. 2, 3, and 4 illustrate pseudocodes for Richardson extrapolation with inverse-free algorithms used to compute {*α*^non-dir^_*A*_(*u*)}, {*α*^screened^_*A*_(*u*)}, and 
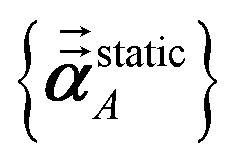
, respectively. The array indices in these figures correspond to what is actually needed in the computer program: (1) the atom number appears as an array index rather than as a subscript, (2) the screening increment does not appear as an array index because temporary results for each screening increment are computed in-place, (3) the imfreq integration point *u* appears as an array index only where needed, *etc.*Fig. 5 illustrates a pseudocode for computing the screened dispersion coefficients and QDO parameters. On each figure, the OpenMP parallelized loops are marked with ‘(this loop index is parallelized)’.

**Fig. 4:**
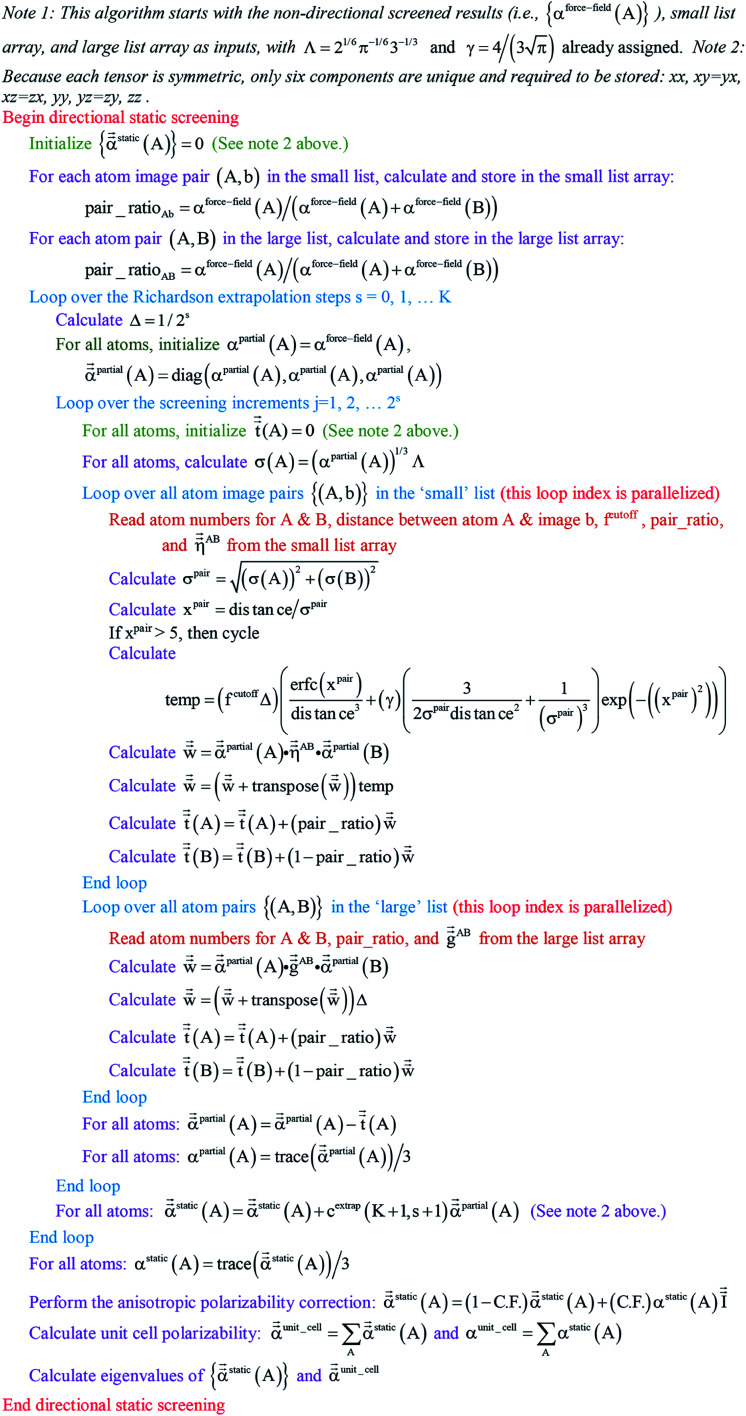
Pseudocode for directional static screening to compute the static polarizability tensors 
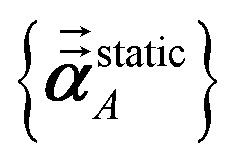
 using inverse-free algorithm and Richardson extrapolation.

**Fig. 5:**
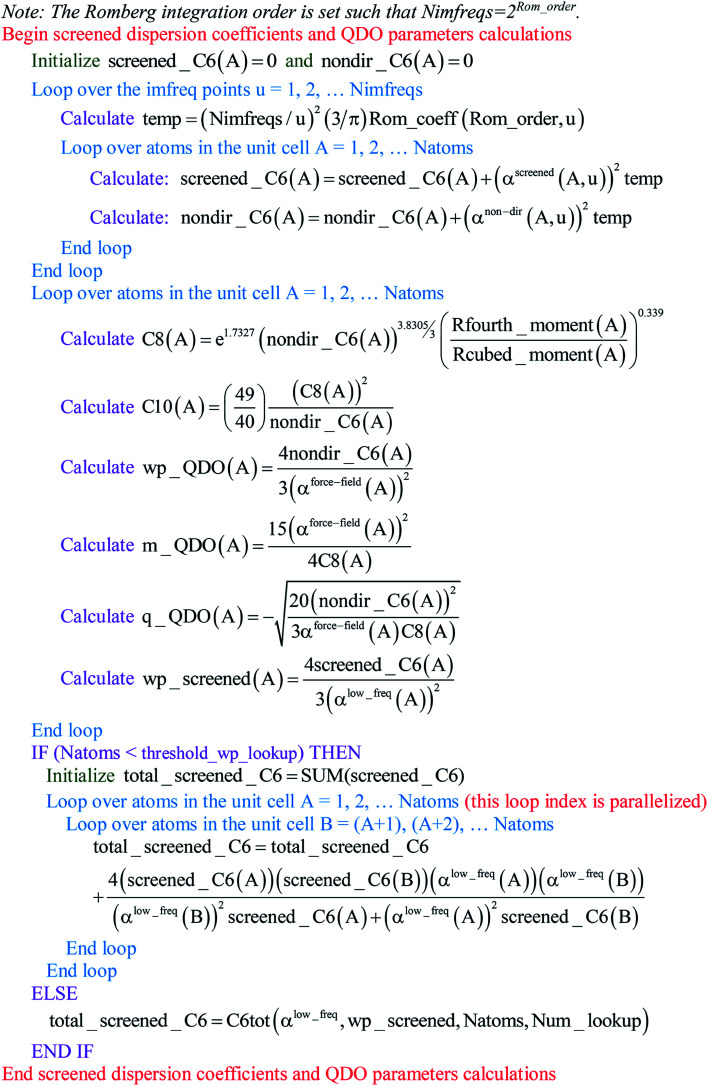
Pseudocode for computing the screened dispersion coefficients and QDO parameters.

## 3. Linear-scaling TS-SCS algorithm

### 3.1 Overview and problem statement

The TS-SCS calculation consists of four main parts: (a) input file reading and unscreened calculation using the TS scaling law^4^ to compute *α*^TS^ and *C*^TS^_6_ for each atom, (b) setting up the ‘small’ and ‘large’ atom pair lists, (c) self-consistent screening to computed the TS-SCS polarizability at each imfreq point, and (d) Casimir–Polder integration to compute the TS-SCS *C*_6_ coefficients. Just as for MCLF, we used Romberg integration with 16 imfreq points to compute *C*_6_ for TS-SCS.

Step (b) follows a procedure analogous to that described in Section 2.2 with the following differences. For the ‘small’ list, similar information was stored for TS-SCS as for MCLF, except *f*_MBS_^*A*,*b*^ does not need to be stored for TS-SCS. For the ‘large’ list, only 
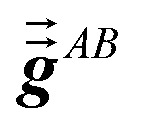
 needs to be stored and 
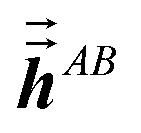
 does not need to be stored for TS-SCS. This allowed each pair in the ‘large’ list for TS-SCS to be stored in a single cache line (*i.e.*, 8 double-precision real numbers), while two cache lines were used to store data for each pair in the ‘large’ list of MCLF analysis. (Stored data for each pair in the ‘large’ list of MCLF analysis required slightly less than two cache lines, but this was rounded up to two whole cache lines to give different atom pairs their own cache lines.) Using whole cache lines provides a slight simplification for tasks that parallelize over atom pairs in the ‘large’ list.)

For MCLF analysis, the smooth cutoff function described in the companion article was used.^1^ This same smooth cutoff function could also be used for TS-SCS. However, for consistency with prior literature,^40^ we used the sharp cutoff function

63 *f*_cutoff_(*d*_*Ab*_) = (1 − *H*(*d*_*Ab*_ − *d*_*cutoff*_))

for TS-SCS analysis, where *d*_*Ab*_ = *r*^*AB*,*L*^ is the distance between atom *A* and the image *b* in bohr. *d*_cutoff_ is the dipole interaction cutoff length. *H* is the Heaviside step function. In this article as well as the companion one,^1^ we set *d*_cutoff_ to 50 bohr.

Eqn (1) is analogous to solving for induced dipole moments in a polarizable force-field64
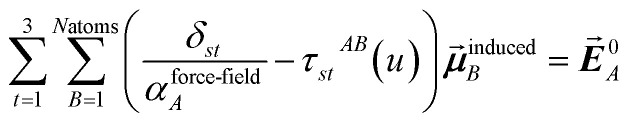
where ***E⃑***^0^_*A*_ is the electric field acting on site *A* due to permanent charges, permanent multipoles, and externally applied electric field. 
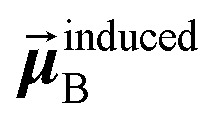
 is the dipole moment induced on site *B*. *α*^force-field^_*A*_ is the polarizability of site *A*. Specifically, setting *α*^force-field^_*A*_ to *α*^unscreened^_*A*_(*u*) and ***E⃑***^0^_*A*_ to (1, 0, 0) yields 
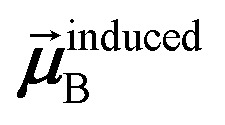
 that gives the first column of 
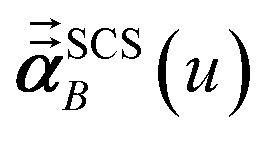
. Solving again with ***E⃑***^0^_*A*_ set to (0, 1, 0) yields 
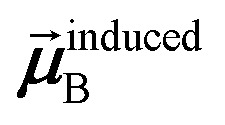
 that gives the second column of 
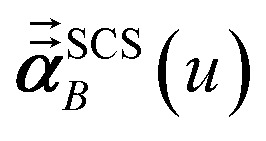
. Finally, solving with ***E⃑***^0^_*A*_ set to (0, 0, 1) yields 
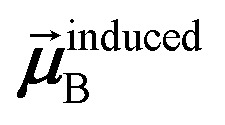
 that gives the third column of 
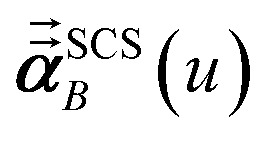
. Hence, a mathematical procedure that is optimized for solving the polarizability eqn (64) can be an efficient way to solve the TS-SCS eqn (1).

As described by Applequist *et al.*, the coefficients matrix in the polarizability equation can be expressed as a two-dimensional array (*i.e.*, as a matrix with rows and columns) by combining spatial and atomic indices.^34^ Since there are three spatial components (*i.e.*, *x*, *y*, and *z*) in the dipole moment for each atom, the coefficients matrix for the polarizability equation (see eqn (1) and (64)) has *N*rows = 3 × *N*atoms and an equal number of columns.^34^ This coefficients matrix is formed by splicing together *N*atoms × *N*atoms blocks, where each block is a 3 × 3 unit for the spatial indices.^34^ From a programming perspective, it is simply a matter of choice as to whether to employ a two-dimensional array with combined atomic and spatial indices or to keep the atomic and spatial indices as distinct array dimensions. Equivalent mathematical problems arise in both cases. Regardless, a large-sized coefficients array arises when there is a large number of atoms in the unit cell.

Developing algorithms to solve large linear equation systems (*e.g.*, **Ax** = **B**) played a key role in the history of electronic computing.^41–44^ (The typeface is used to distinguish **A** as a matrix from *A* as an atom.) Here, we are particularly interested in solving large systems of sparse linear equations, and many different algorithms have been described in the prior literature for doing this.^42,43,45,46^ Krylov subspace techniques solve a linear equation system using iterations in which some error norm is minimized in a Krylov subspace whose order increases with iteration number.^47,48^ A Krylov subspace of order *n* defined by matrix **M** and vector ***W*** is^48^

65 *K*_*n*_(**M**,***W***) = span[***W***, **M*W***, **M**^2^***W***, **M**^3^***W***, …**M**^*n*−1^***W***]

A key consideration is how to generate an orthonormal vector basis that spans the Krylov subspace sequence without having to store vectors from all prior iterations.^49,50^ For a Hermitian matrix **M**, short-term recurrences can be constructed that span the Krylov subspace while requiring only vectors from a few recent iterations to be explicitly stored (*e.g.*, from the current and two most recent prior iterations).^44,47,48^ By definition, a Hermitian matrix equals its Hermitian conjugate (*i.e.*, complex conjugate transpose). Conjugate gradient algorithms are a widespread class of Krylov subspace techniques.^43,47,48,51^

Conditioning plays a key role in conjugate gradient type methods.^42,46,47,51,52^ Conditioning multiplies the original coefficients matrix by one or more matrices to transform the original linear equation **Ax** = **B** into a new linear equation **M***y* = ***W*** that is easier to solve.^42,46,47,51,52^ Either left multiplication or right multiplication, or a combination of both, can be used during conditioning.^42,47,51,52^ Conditioning can accomplish one of more of the following:^42,47,51,52^

(i) If the original coefficients matrix **A** is not Hermitian, conditioning may make the new coefficients matrix **M** Hermitian., or

(ii) If the original coefficients matrix **A** is not positive (semi)definite, conditioning may make the new coefficients matrix **M** positive (semi)definite., or

(iii) If the original coefficients matrix **A** is not square, conditioning may make the new coefficients matrix **M** square. This is important for solving least-squares problems., or

(iv) To improve convergence speed, conditioning may make the eigenspectrum of **M** more clustered than that of **A**. After conditioning, conjugate gradient methods minimize some error norm within the Krylov subspace *K*_*n*_(**M**,***W***).

Recently, truncated conjugate gradient algorithms were used to solve for induced dipole moments, polarization energies, and the corresponding forces in polarizable force fields.^53,54^ Aviat *et al.* used the Orthomin^47^ conjugate gradient algorithm (also called Hestenes' and Stiefel's method^55^) truncated at fixed order to solve the multibody polarization equations efficiently without introducing spurious energy drifts during molecular dynamics simulations using polarizable force fields.^53,54^ The key limitation of the Orthomin conjugate gradient algorithm is the coefficients matrix **M** in the linear equation **M***y* = ***W*** must be Hermitian positive definite.^47^ Although the polarization eqn (64) contains a Hermitian coefficients matrix, we do not know whether or not it is always positive definite. The coefficients matrix is positive definite if and only if all of its eigenvalues are positive.^56^ For many common circumstances the coefficients matrix in this polarization equation is likely to be positive definite, but we do not have any information at hand about exceptions that could potentially give an indefinite coefficients matrix in the polarization equation. If the coefficients matrix **M** is indefinite, the Orthomin conjugate gradient algorithm will fail if 〈*z*^(*i*)^|**M***z*^(*i*)^〉 = 0 at any iteration, when *z*^(*i*)^ ≠ 0.

Herein, **x**^(*i*)^ is the estimated **x** at iteration *i*, *r*^(*i*)^ is the residual, and *z*^(*i*)^ is the conditioned residual:^47^

66 *r*^(*i*)^ = **B** − **Ax**^(*i*)^

67 *z*^(*i*)^ = **C***r*^(*i*)^ = ***W*** − **M***y*^(*i*)^

In this article, the dot product of two vectors ***v*** and ***w*** is defined as68
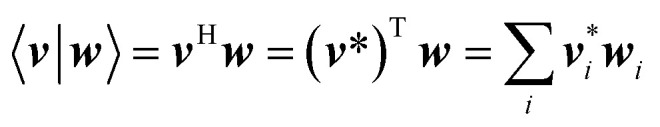


Three old conjugate gradient algorithms are Orthomin, Orthodir, and Orthores.^47,50^ As mentioned above, Orthomin can fail for indefinite coefficient matrices. Orthores is algebraically equivalent to Orthomin, and Orthores converges if and only if Orthomin converges.^47^

The Orthodir conjugate gradient algorithm can work for both positive definite and indefinite Hermitian coefficients matrix **M**.^47^ However, it suffers from the accumulation of round-off errors. In the Orthodir algorithm, the *y*-search direction at each successive iteration is computed as

69 *p*^(*i*)^ = **M***p*^(*i*−1)^ − *ς*^(*i*)^*p*^(*i*−1)^ − *ϑ*^(*i*)^*p*^(*i*−2)^

where *ς*^(*i*)^ and *ϑ*^(*i*)^ are chosen to fulfill some chosen conjugacy condition.^47^ In exact arithmetic, eqn (69) would enforce orthogonality between **M***p*^(*i*)^, **M***p*^(*i*−1)^, and **M***p*^(*i*−2)^. Because the choice of direction *p*^(*i*)^ does not explicitly depend on the residual's value (see eqn (69)), a buildup of round-off errors over many iterations can cause the chosen *z*-search direction **M***p*^(*i*)^ to become uncorrelated to the residual's value. When this occurs, the Orthodir algorithm does not operate as intended and may fail to converge.

Conjugate gradient of the normal equations rearranges the linear equation system so that the coefficients matrix is **M** = **A**^H^**A**, thereby ensuring a positive semidefinite Hermitian coefficients matrix **M**.^42,43,47^ Because each eigenvalue of **M** = **A**^H^**A** is the squared magnitude of the corresponding eigenvalue of **A**, all eigenvalues of **M** = **A**^H^**A** are non-negative real-valued. Two common variants are CGNE (also called Craig's method^57^) and CGNR.^42,43,47,55^ Unfortunately, both of these algorithms often converge slowly.^42,43,47^ For example, we programmed CGNE and CGNR for TS-SCS analysis and tested them on the C_50_H_24_ polyacene, but convergence was not reached within 100 iterations for at least one direction at a single imfreq point. For these tests, we used the same convergence threshold as for the FCR algorithm; namely, <10^−5^ for the maximum absolute value of each conditioned residual component.

### 3.2 The FCR algorithm

This provided motivation to develop a failproof conjugate residual (FCR) algorithm that converges robustly and resists round-off errors. FCR solves any linear equation system with Hermitian coefficients matrix

70 **Ax** = **B**

for an exact solution (within the convergence tolerance) if one exists or for a conditioned least-squares solution if no exact solution exists. The matrices **x** and **B** may contain a single column or more than one column. Matrix **A** is non-singular if and only if its determinant is nonzero (*i.e.*, all of its eigenvalues are non-zero). In this case, matrix **A** is invertible and the equation **Ax** = **B** has the unique solution **x** = **A**^−1^**B**. If matrix **A** is singular, then **Ax** = **B** has either no solution or an infinite number of solutions. If the linear equation system is consistent (*i.e.*, has at least one solution), this FCR algorithm returns one of its solutions. When matrix **B** has more than one column, the FCR algorithm is applied separately to each column. When **B** = **I** (identity matrix), the method solves for **x** = **A**^−1^, the inverse of matrix **A**.

The goal of conditioning is to rotate and scale matrix **A** to improve convergence speed:

71 **M** = **CAC**^H^

72 **M***y* = **CAC**^H^*y* = **C**(**B** − **Ax**_0_) = ***W***

where

73 **x** = **C**^H^*y* + **x**_0_

Here, **x**_0_ is any initial guess for **x**. This shift always makes *y*_0_ = 0 as the initial guess for *y*. The conditioning matrix **C** must be non-singular. Since **A** is Hermitian, the matrix **M** will automatically be Hermitian for any conditioning matrix **C**:

74 **M**^H^ = (**CAC**^H^)^H^ = (**C**^H^)^H^**A**^H^**C**^H^ = **CAC**^H^ = **M**

In this article, the dot product of two vectors ***v*** and ***w*** is defined as75
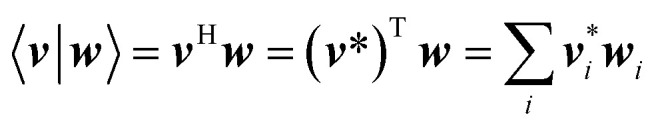
A Hermitian matrix **M** = **M**^H^ can be freely moved between sides of the dot product. For example,

76 〈**MM***q*|*p*〉 = 〈**M***q*|**M***p*〉 = 〈*q*|**MM***p*〉 = *q*^H^**M**^2^*p*

As commonly known, the Hermitian conjugate of a matrix product follows

77 (*ϒΛ*)^H^ = *Λ*^H^*ϒ*^H^

If the linear equation system (*i.e.*, eqn (70)) is inconsistent (*i.e.*, has no exact solution), this FCR algorithm returns a statement that the linear equation system has no exact solution along with a value *y* = *y*^FCR^ that minimizes the least-squares problem

78 Minimize *F* = 〈*z*|*z*〉 = |***W*** − **M***y*|^2^

This represents a best possible choice for *y* irrespective of whether an exact solution to eqn (70) exists. There are some applications where this least-squares fit has utility even if an exact solution to eqn (70) does not exist. (Note: the case where **M***y* = ***W*** is consistent arises as the special case of eqn (78) where the least-squares error is simply zero.)

As commonly defined, the kernel of **M** is the set of all *y* values that solve **M***y* = 0,

79 kernel(**M**) ⇒ **M***y* = 0

When **M** is non-singular, *y* = 0 is the only vector in the kernel. When **M** is singular, the kernel includes *y* = 0 along with an infinite number of non-zero vectors. Manifestly, any vector from the kernel of **M** can be added to *y*^FCR^ without changing the conditioned residual value. Therefore, the set of all equally good (aka ‘best’) solutions to eqn (78) is

80 *y*^all^ = *y*^FCR^ + span[kernel(**M**)]

If the kernel(**M**) is known, this allows calculating the entire set of {*y*^all^} that give the same minimum value of *F*. If **M***y* = ***W*** is consistent, this is the set of *y* values yielding *F* = 0 (*i.e.*, satisfying eqn (72)). The number of vectors in {*y*^all^} is one if **M** is non-singular; otherwise, {*y*^all^} contains an infinite number of vectors.

The FCR method minimizes the conditioned residual norm

81 *Γ*^(*i*)^ = 〈*z*^(*i*)^|*z*^(*i*)^〉

Each iteration involves two search directions, *p*^(*i*)^ and *q*^(*i*)^:

82 *y*^(*i*)^ = *y*^(*i*−1)^ + *γ*^(*i*)^*p*^(*i*)^ + *τ*^(*i*)^*q*^(*i*)^

83 *z*^(*i*)^ = *z*^(*i*−1)^ − *γ*^(*i*)^**M***p*^(*i*)^ − *τ*^(*i*)^**M***q*^(*i*)^

subject to the constraints

84 *p*^(*i*)^, *q*^(*i*)^ ∈ *K*_2*i*_(**M**,***W***)

85 *K*_2*i*_(**M**,***W***) = span[*p*^(1)^, *q*^(1)^, …*p*^(*i*)^, *q*^(*i*)^]

In exact arithmetic, all *z*-search directions are chosen to be orthogonal (aka ‘conjugate’):

86 〈**M***q*^(*i*)^|**M***q*^(*j*≠*i*)^〉 = 〈**M***p*^(*i*)^|**M***q*^(*j*)^〉 = 〈**M***p*^(*i*)^|**M***p*^(*j*≠*i*)^〉 = 0

The conditioned residual component along *z*-search directions **M***p*^(*i*)^ and **M***q*^(*i*)^ are removed in iteration *i*:

87 〈**M***q*^(*i*)^|*z*^(*i*)^〉 = 〈**M***p*^(*i*)^|*z*^(*i*)^〉 = 0

This yields88
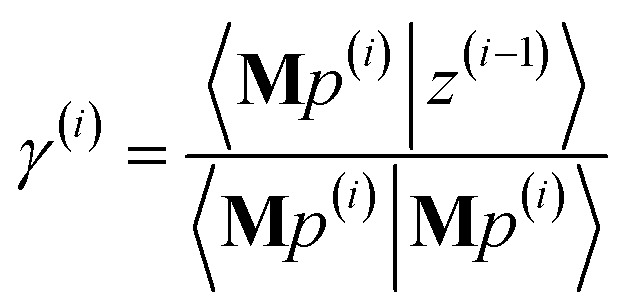
89
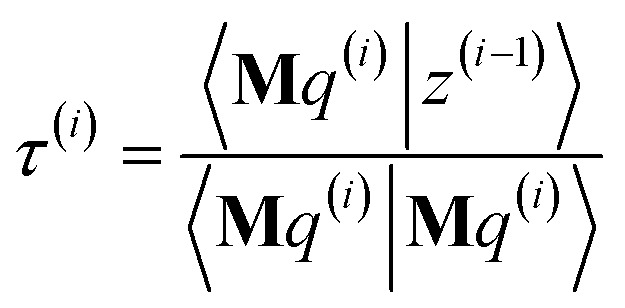
Substituting eqn (88), (89), and (83) into (81) yields (see ESI† for derivation):

90 *Γ*^(*i*)^ = *Γ*^(*i*−1)^ − |*γ*^(*i*)^|^2^〈**M***p*^(*i*)^|**M***p*^(*i*)^〉 − |*τ*^(*i*)^|^2^〈**M***q*^(*i*)^|**M***q*^(*i*)^〉

After computing the new values of *z*^(*i*)^ and 〈**M***z*^(*i*)^|**M***z*^(*i*)^〉, the following convergence tests are performed: (1) if the absolute value of every conditioned residual component is less than a chosen CR_convergence_tol, the algorithm exits and returns a message that the linear equation system is consistent and returns the current value of *y* (which is a nearly exact solution). (2) Else if 
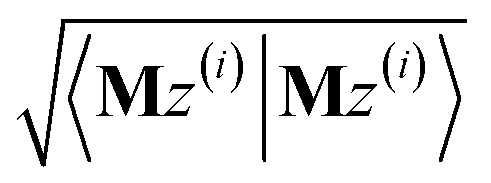
 is less than a chosen **M***z*_length_tolerance, the algorithm exits and returns a statement that the linear equation system is inconsistent and returns the current value of *y* (which is the solution to the least-squares problem in eqn (78)). (3) Else if the number FCR iterations reaches max_CR_steps, the algorithm exits with a message the maximum number of FCR iterations was reached and returns the current value of *y* (which is the partial solution to the FCR problem). (4) Else the algorithm continues to the next iteration.

In some circumstances (A) an exit due to condition (3) is considered an error, but in other circumstances (B) it is the preferred exit condition. In this case (A), the goal is to reach a specified precision rather than a specified number of FCR iterations. In this case, max_FCR_iterations is set to a large value (*e.g.*, 100–1000) and exit *via* condition (3) represents a convergence failure. In case of convergence failure, the algorithm could be restarted using a different (and hopefully better) conditioning matrix. In case (B), the goal is to reach a specified number of FCR iterations rather than a specified precision. In classical atomistic simulations *via* polarizable force fields, using a fixed number of conjugate gradient iterations leads to continuous forces with enhanced energy conservation; therefore, exiting after a small constant number of iterations is preferred.^53,54^ This behavior can be achieved by setting max_FCR_iterations to a small whole number (*e.g.*, ∼5) and setting FCR_convergence_tol and **M***z*_length_tolerance to very small values.

The direction *p*^(*i*)^ is chosen to ensure

91 〈**M***p*^(*i*)^|*z*^(*i*−1)^〉 = 〈**M***z*^(*i*−1)^|**M***z*^(*i*−1)^〉 > 0

when the linear equation system is consistent. This guarantees

92 0 ≤ *Γ*^(*i*)^ < *Γ*^(*i*−1)^

even if round-off errors occurred during prior computations. Hence the method makes forward progress towards the solution in each and every iteration. As derived in the ESI,† these requirements are fulfilled by choosing

93 *p*^(*i*)^ = **M***z*^(*i*−1)^ − *β*^(*i*)^*p*^(*i*−1)^ − *ξ*^(*i*)^*q*^(*i*−1)^

94
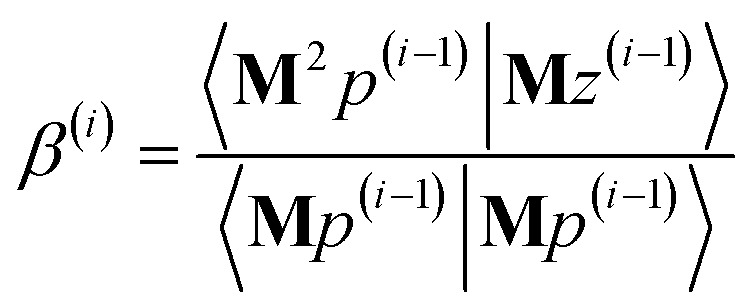
95
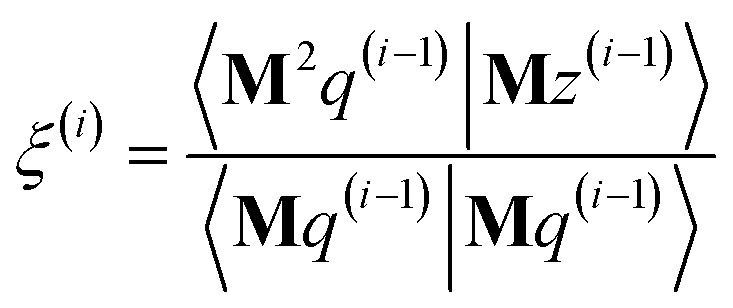
The length of *p*^(*i*)^ is non-zero (see ESI† for derivation):

96 〈*p*^(*i*)^|*p*^(*i*)^〉 = 〈**M***z*^(*i*−1)^|**M***z*^(*i*−1)^〉 + 〈*β*^(*i*)^*p*^(*i*−1)^ + *ξ*^(*i*)^*q*^(*i*−1)^|*β*^(*i*)^*p*^(*i*−1)^ + *ξ*^(*i*)^*q*^(*i*−1)^〉 > 0

As explained in the ESI,† the Krylov subspaces will be spanned with resistance to round-off errors by assigning *q*^(*i*)^*via* the following ordered sequence:97
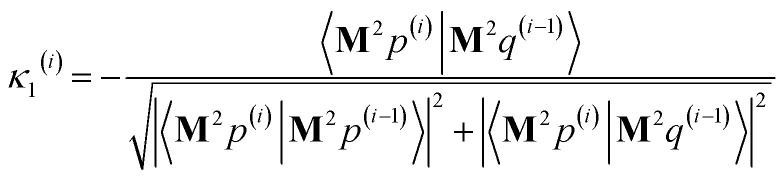
98
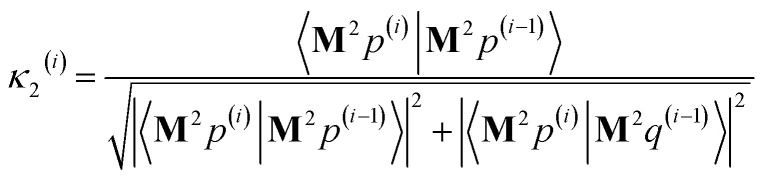

99 *q*^(*i*)^ ← *κ*_1_^(*i*)^**M**^2^*p*^(*i*−1)^ + *κ*_2_^(*i*)^**M**^2^*q*^(*i*−1)^

100
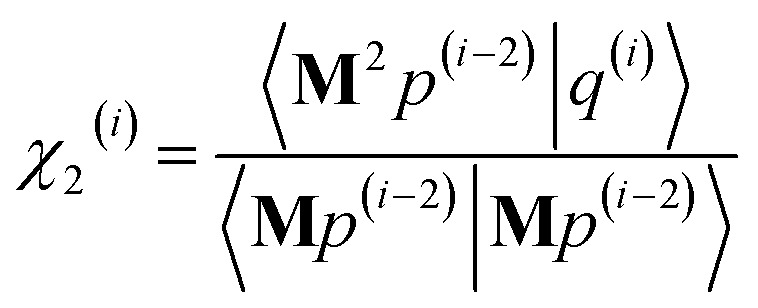
101
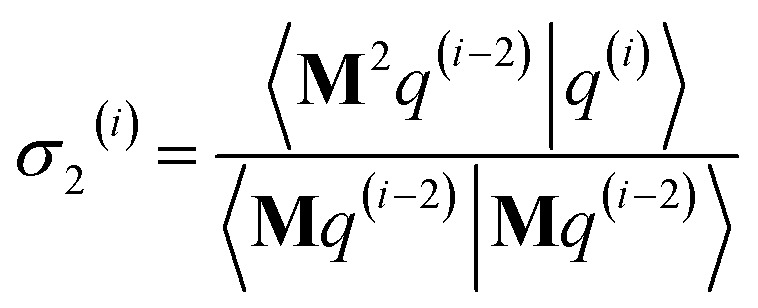

102 *q*^(*i*)^ ← *q*^(*i*)^ − *χ*_2_^(*i*)^*p*^(*i*−2)^ − *σ*_2_^(*i*)^*q*^(*i*−2)^

103
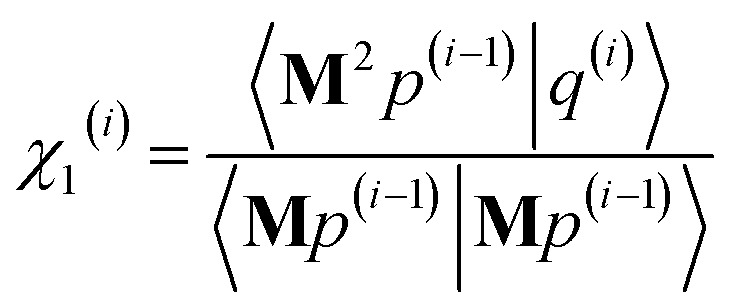
104
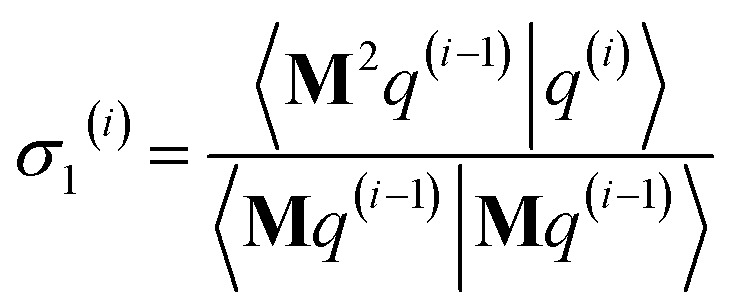

105 *q*^(*i*)^ ← *q*^(*i*)^ − *χ*_1_^(*i*)^*p*^(*i*−1)^ − *σ*_1_^(*i*)^*q*^(*i*−1)^

106
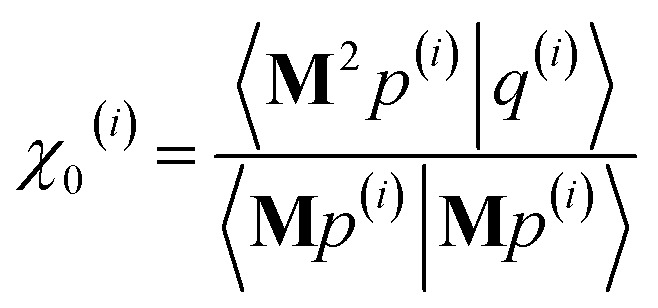

107 *q*^(*i*)^ ← *q*^(*i*)^ − *χ*_0_^(*i*)^*p*^(*i*)^

Eqn (106) and (107) ensure that

108 〈**M***q*^(*i*)^|**M***p*^(*i*)^〉 = 0

even if round-off errors occurred during prior computations. With this result, eqn (103)–(105) and (93)–(95) ensure that

109 〈**M***q*^(*i*)^|**M***p*^(*i*−1)^〉 = 〈**M***q*^(*i*)^|**M***q*^(*i*−1)^〉 = 0

110 〈**M***p*^(*i*)^|**M***p*^(*i*−1)^〉 = 〈**M***p*^(*i*)^|**M***q*^(*i*−1)^〉 = 0

even if round-off errors occurred during prior computations. In exact arithmetic, the length of *q*^(*i*)^ is non-zero in all iterations before the last one, and may either be zero or non-zero on the last iteration (see ESI† for derivation).

The algorithm is initialized with

111 *p*^(*i*≤0)^ = *q*^(*i*≤0)^ = 0

112 *p*^(1)^ = **M***z*^(0)^ = **M*W***

113
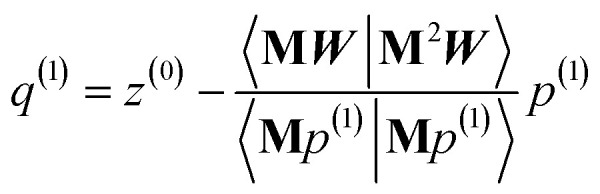


All denominators in the method have the form 〈**M***p*^(*i*)^|**M***p*^(*i*)^〉 or 〈**M***q*^(*i*)^|**M***q*^(*i*)^〉. Since **M** is Hermitian, its eigenvalues are real-valued. Since the eigenvalues of **M**^2^ are the squared eigenvalues of **M**, then all eigenvalues of **M**^2^ are non-negative. Thus, 〈**M***p*^(*i*)^|**M***p*^(*i*)^〉 and 〈**M***q*^(*i*)^|**M***q*^(*i*)^〉 are non-negative. As derived in Section S2 of the ESI,† 〈**M***p*^(*i*)^|**M***p*^(*i*)^〉 = 0 can only occur if 〈**M***z*^(*i*−1)^|**M***z*^(*i*−1)^〉 = 0 which would have already led to the algorithm exiting due to convergence in iteration (*i* − 1). 〈**M***q*^(*i*)^|**M***q*^(*i*)^〉 = 0 may arise from (a) round-off errors or (b) on the last iteration if all components of *z*^(*i*)^ not in kernel(**M**) are already made zero by the **M***p*^(*i*)^ search direction. The algorithm includes division by zero protection at all steps. Specifically, the value of the parameter *γ*^(*i*)^ (eqn (88)), *τ*^(*i*)^ (eqn (89)), *β*^(*i*)^ (eqn (94)), *ξ*^(*i*)^ (eqn (95)), *κ*_1_^(*i*)^ (eqn (97)), *κ*_2_^(*i*)^ (eqn (98)), *χ*_2_^(*i*)^ (eqn (100)), *σ*_2_^(*i*)^ (eqn (101)), *χ*_1_^(*i*)^ (eqn (103)), *σ*_1_^(*i*)^ (eqn (104)), or *χ*_0_^(*i*)^ (eqn (106)) is set to zero if the denominator appearing in the respective equation is zero. (As derived in the ESI,† this is the rigorously correct parameter value in the limit of zero denominator.)

In exact arithmetic, the algorithm converges to the exact solution in a finite number of iterations. The conditioned residual is eliminated along two directions in each iteration (*i.e.*, along **M***p*^(*i*)^ and **M***q*^(*i*)^). In exact arithmetic, these directions are orthogonal to all previous directions **M***p*^(*j*<*i*)^ and **M***q*^(*j*<*i*)^. The number of independent directions in the residual *z*^(*i*)^ is less than or equal to *N*rows, where *N*rows is the number of rows in matrix **M**. Therefore, in exact arithmetic the algorithm converges to the exact result in at most ceiling(*N*rows/2) iterations. In finite arithmetic, the FCR algorithm resists round-off errors as described above.

Further convergence analysis can be obtained by expanding ***W*** in terms of the eigenvectors {***V⃑***_*j*_} of **M**:114
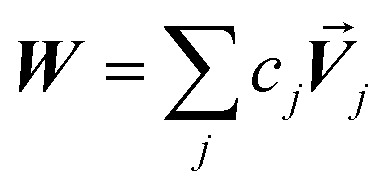
The building blocks of the Krylov subspace are thus115
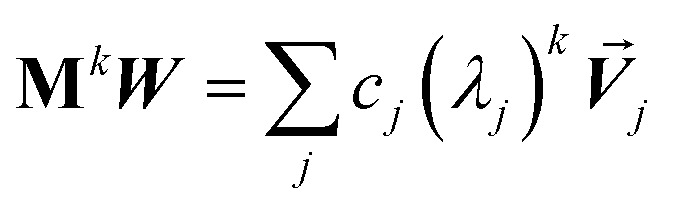
where {*λ*_*j*_} are the corresponding eigenvalues of **M**. These building blocks can generate at most *Ξ* linearly independent vectors, where ***W*** is a linear combination of eigenvectors of **M** having exactly *Ξ* distinct eigenvalues *λ*_*j*_. Since each FCR iteration searches 2 independent directions, in exact arithmetic the algorithm will converge in at most ceiling(*Ξ*/2) iterations. For example, if matrix **M** contains *N*rows = 1 million, but matrix **M** has only 12 distinct eigenvalues, then FCR will converge to the solution in at most 6 iterations in exact arithmetic.

FCR expands *y*^(*i*)^ as116
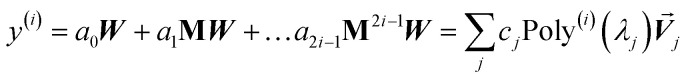
where {*a*_*k*_} are some optimized coefficients and117
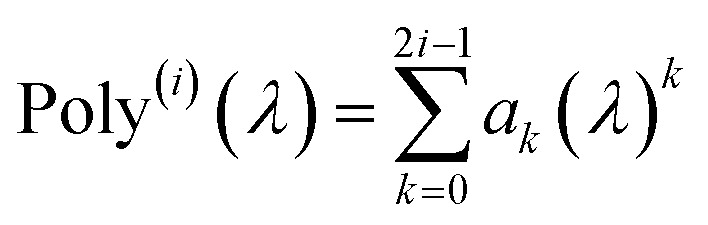
is the associated polynomial. Expanding,118
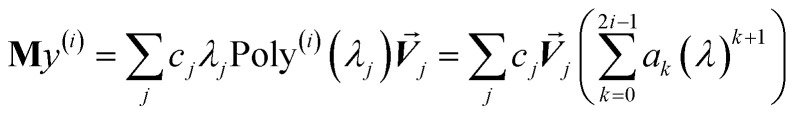


The conditioned residual value at each FCR iteration is given by119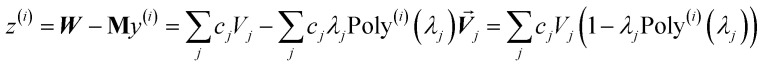 Therefore, the exact solution is reached when

120 Poly^(*i*)^(*λ*_*j*_) ⇒ 1/*λ*_*j*_

The FCR convergence properties are thus dictated by the difficulty of representing {1/*λ*_*j*_} *via* a polynomial Poly^(*i*)^(*λ*). When the eigenvalues are close to one, this is trivial because121
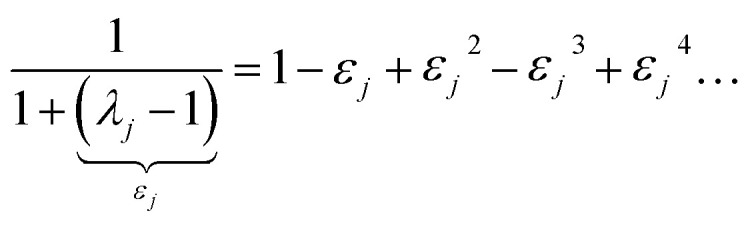
When the eigenvalues are extremely spread out in values, then it takes a higher-order polynomial (and hence a larger number of FCR iterations) to make Poly^(*i*)^(*λ*) ≈ 1/*λ* for all of the present eigenvalues. Thus, the primary goal of conditioning (eqn (71)) is to make the eigenvalues less spread out in values.

All FCR equations presented here apply to both complex-valued and real-valued matrices. If **M** or ***W*** are complex-valued, then {*p*^(*i*)^}, {*q*^(*i*)^}, {*z*^(*i*)^}, and *γ*^(*i*)^, *τ*^(*i*)^, *β*^(*i*)^, *ξ*^(*i*)^, *κ*_1,2_^(*i*)^, *χ*_0,1,2_^(*i*)^, *σ*_1,2_^(*i*)^ must be declared as complex variables. On the other hand, if **M** and ***W*** are real-valued, then all of those quantities should be declared as real variables. For solving the TS-SCS and polarizability equations, the corresponding matrices were real-valued.

For singular **M**, **M***y* does not contain any contributions from any zero eigenvalues, because multiplying by **M** multiplies the corresponding basis eigenvector by its eigenvalue (which is zero) as shown in eqn (118). Consequently, if ***W*** contains any nonzero contribution (*i.e.*, *c*_*j*_ ≠ 0) from an eigenvector ***V⃑***_*j*_ having associated zero eigenvalue (*i.e.*, *λ*_*j*_ = 0), then the linear equation system is inconsistent. Since only the basis eigenvectors appearing in ***W*** need to be represented by **M***y* (see eqn (116)–(119)), the linear equation system is consistent and solved exactly by the FCR algorithm if and only if all nonzero contributions (*i.e.*, *c*_*j*_ ≠ 0) of ***W*** have non-zero eigenvalues (*i.e.*, *λ*_*j*_ ≠ 0). Therefore, even **M***y* = ***W*** systems for singular **M** can be solved exactly by FCR as long as the zero eigenvalues (*i.e.*, *λ*_*j*_ = 0) have zero contribution (*i.e.*, *c*_*j*_ = 0) to ***W***. In this case, the returned solution *y*^FCR^ has no contribution from any eigenvector whose associated eigenvalue is zero (*i.e.*, *y*^FCR^ is orthogonal to kernel(**M**)).

As shown in eqn (118), **M***y* cannot contain any eigenvectors from the kernel(**M**), because each term contributing to **M***y* contains the factor (*λ*_*j*_)^*k*+1≥1^ which is zero for any *V*_*j*_ in kernel(**M**). Consequently, any *V*_*j*_ in kernel(**M**) having non-zero *c*_*j*_ cannot be removed from the conditioned residual (see eqn (118) and (119)). Therefore, when **M***y* = ***W*** is inconsistent, minimizing |***W*** − **M***y*|^2^ corresponds to removing all conditioned residual components that are not in kernel(**M**), while conditioned residual components in kernel(**M**) remain at their initial levels (*i.e.*, same contribution as in ***W***).

The ESI† explains further details. Section S2.1† defines the problem definition and matrix conditioning. Section S2.2† contains eigenvalue decomposition analysis using Krylov subspaces. Section S2.3† defines conjugate search directions that span the Krylov subspaces. Section S2.4† details vector lengths in exact arithmetic. Section S2.5† explains robust convergence and round-off error resistance. Section S2.6† presents a step-by-step computational procedure of this FCR algorithm.

### 3.3 Using FCR to solve the TS-SCS equations

The following procedure was used to solve the TS-SCS equations using the FCR algorithm. First, the following quantities were defined122
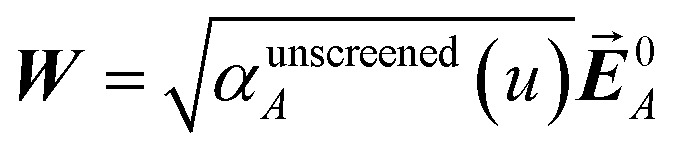
123
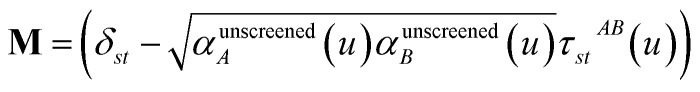
for one value of *u* with ***E⃑***^0^_*A*_ set to (1, 0, 0). The FCR algorithm was then used to solve the linear equation **M***y* = ***W*** for *y* which was solved for 
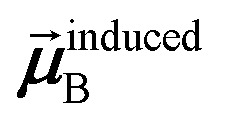
 by multiplying by 
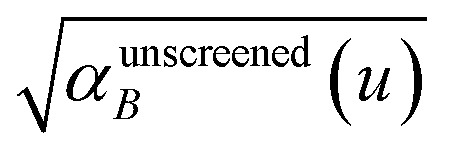
:124
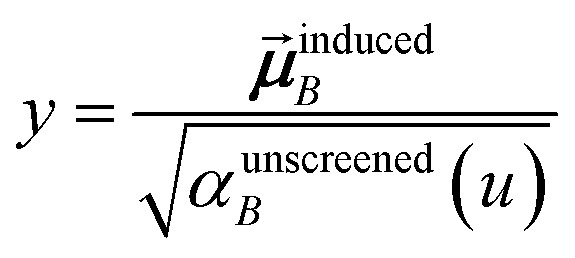

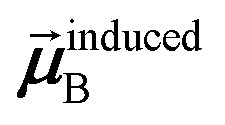
 gives the first column of 
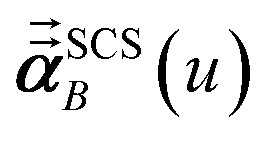
. In this scheme, 
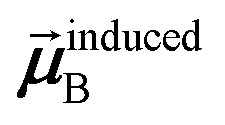
 corresponds to ‘**x**’ in the linear equation system ‘**Ax** = **B**’, and **B** corresponds to the externally applied electric field, {***E⃑***^0^_*A*_}. The conditioning matrix **C** is diagonal with 
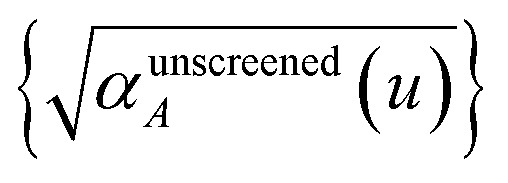
 along the diagonal. Solving again with ***E⃑***^0^_*A*_ set to (0, 1, 0) yields 
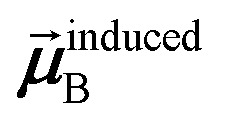
 that gives the second column of 
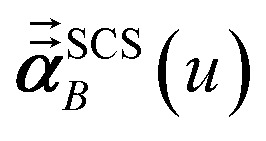
. Finally, solving with ***E⃑***^0^_*A*_ set to (0, 0, 1) yields 
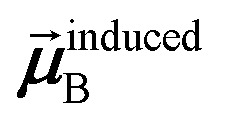
 that gives the third column of 
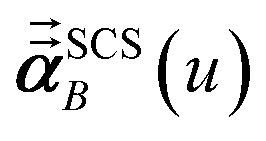
. This entire process is repeated at each imfreq point *u*.

**Fig. 6:**
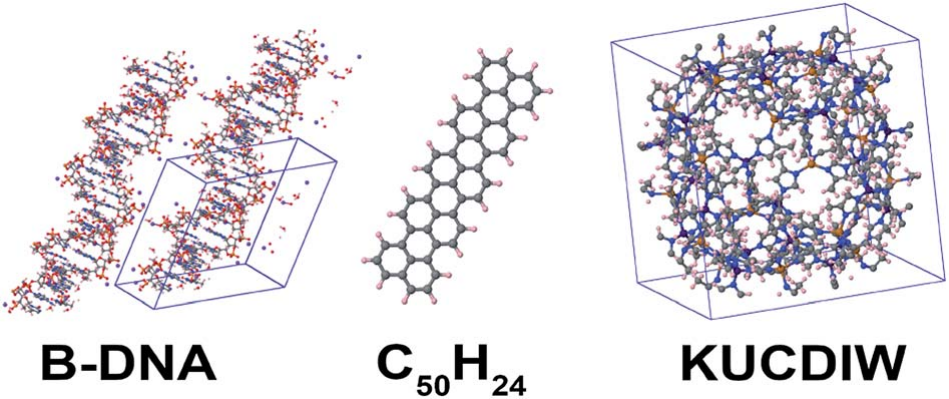
Three diverse chemical structures used to study the TS-SCS convergence properties: the B-DNA decamer, the C_50_H_24_ polyacene, and the KUCDIW metal–organic framework. The atoms are colored by chemical element: grey (C), pink (H), blue (N), purple (Na), red (O), orange (P), copper (Cu), indigo (B).

**Fig. 7:**
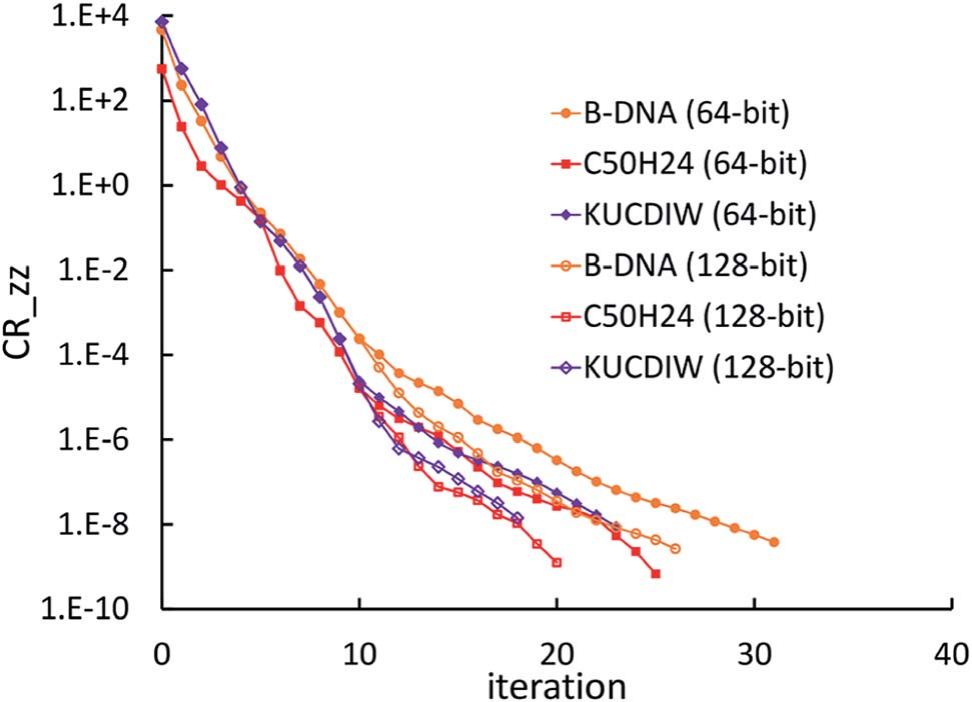
Plot of the conditioned residual norm (*i.e.*, CR_zz) *versus* the FCR iteration number for three diverse materials: the B-DNA decamer, the C_50_H_24_ polyacene, and the KUCDIW metal–organic framework. The conditioned residual norm decreased rapidly and monotonically with increasing iteration number. The size of the real numbers (64-bit or 128-bit) is indicated.

We now discuss a few computational examples. For the examples we studied, the FCR algorithm indicated the linear equations were consistent and converged to a nearly exact solution. Table 5 shows the FCR algorithm converged to the same solution in the same number of iterations for a large supercell as for the small primitive unit cell of the same material. Table 5 also shows FCR converged to the same solution (within the convergence tolerance) as direct matrix inversion using GEPP. Since the number of diverse atom types in graphene and ice is not large, we explored three more diverse chemical structures shown in Fig. 6. These included a large biomolecule (B-DNA) whose geometry was taken from a prior study,^14^ the C_50_H_24_ polyacene geometry taken from the companion article,^1^ and a metal–organic framework whose geometry was taken from the Computation Ready Experimental (CoRE) metal–organic framework (MOF) database.^58^ We used the MOF having Cambridge Structural Database^59^ code KUCDIW. Electron densities for these three materials were generated in VASP using the PBE functional, a 400 (B-DNA and MOF) and 750 (C_50_H_24_) eV planewave cutoff, and the PAW method. The *k*-point mesh and grid spacing followed previous recommendations.^12^ FCR convergence for these three materials is summarized in Table 6 and Fig. 7. As shown in Fig. 7, the conditioned residual norm decreased rapidly and monotonically with increasing iteration number for all three materials. Table 6 shows that a summed total of up to a couple thousand large matrix-vector multiplies may be required to complete TS-SCS(FCR) analysis across all imfreq points. Convergence was highly efficient, with FCR convergence along one direction for a single imfreq point occurring in ≤31 iterations. Each FCR iteration required four large matrix-vector multiplies.

**Table 5:** Effect of unit cell size on the calculated results and number of FCR iterations required to converge TS-SCS/DDEC6 calculation. The convergence threshold was 10^−5^ as the maximum absolute value of any conditioned residual component. The listed number of FCR iterations to converge was the maximum for any one direction at a single imfreq point. The total FCR iterations and large matrix-vector multiplies are summed over all imfreq points and directions. GEPP results are also listed for the primitive unit cells. The polarizabilities and *C*_6_ dispersion coefficients are in atomic units: (a) per carbon atom for graphene and (b) per water molecule for ice

	Graphene			Ice		
	2 atom primitive cell		20 000 atom supercell	12 atom primitive cell		2 107 392 atom supercell
Algorithm	GEPP	FCR	FCR	GEPP	FCR	FCR
*α* ^static^	20.12	20.12	20.12	8.86	8.86	8.86
*C*_6_	108.66	108.66	108.66	42.48	42.48	42.49
FCR iterations to converge	—	1	1	—	6	6
Total FCR iterations	—	48	48	—	181	181
Large matrix-vector multiplies	—	192	192	—	724	724

**Table 6:** TS-SCS(FCR) convergence performance for three diverse materials: (a) the B-DNA decamer, (b) the C_50_H_24_ polyacene, and (c) the KUCDIW metal–organic framework. The convergence threshold was 10^−5^ as the maximum absolute value of any conditioned residual component. The listed number of FCR iterations to converge was the maximum for any one direction at a single imfreq point. The total FCR iterations and large matrix-vector multiplies are summed over all imfreq points and directions. The total computational time is for a serial (*i.e.*, one computing core) run on the Comet cluster. Data for 64-bit (128-bit) reals is shown outside (inside) parentheses

	B-DNA decamer	C_50_H_24_ polyacene	KUCDIW metal–organic framework
Atoms per unit cell	733	74	1104
FCR iterations to converge	31 (26)	25 (20)	23 (18)
Total FCR iterations	483 (417)	360 (323)	390 (347)
Large matrix-vector multiplies	1932 (1668)	1440 (1292)	1560 (1388)
Total computational time in seconds	58 (104)	0.57 (0.97)	107 (195)

Except where otherwise specified, all computations in this article and the companion article^1^ were performed using 64-bit real numbers. For comparison, computations performed using 128-bit reals are also displayed in Table 6 and Fig. 7. Using 128-bit reals increases computational cost and decreases round-off errors compared to 64-bit reals. Calculations converged in fewer FCR iterations using 128-bit reals, but the overall computational time was higher. The converged results were equivalent within the convergence tolerance.

**Fig. 8:**
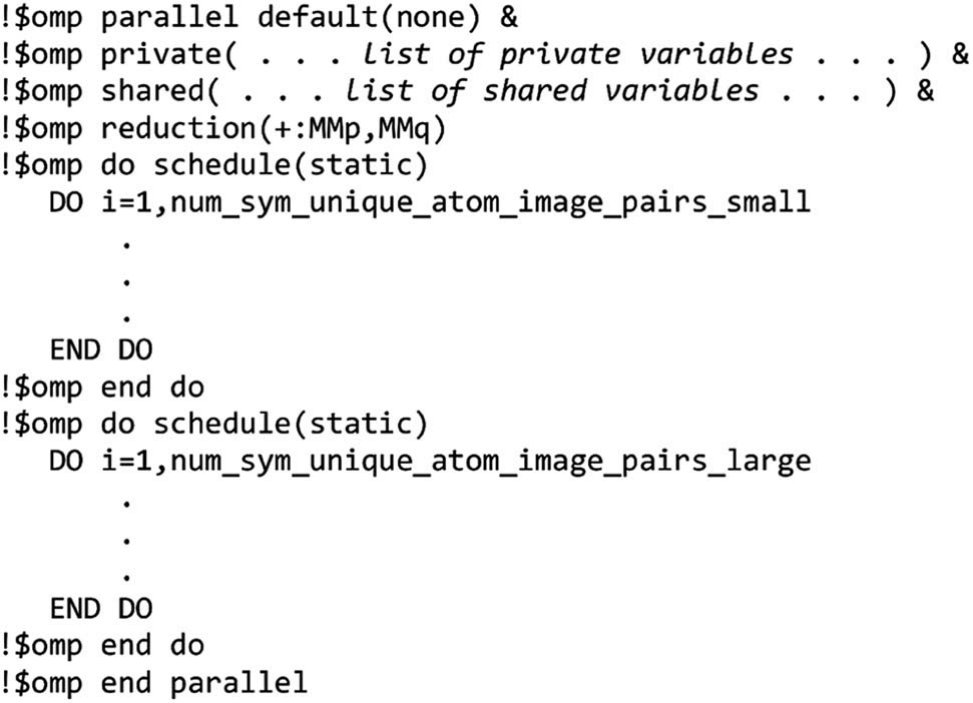
Excerpt of OpenMP parallelized Fortran code that performs the operations **MM***p*(:,1) = **M** × **M***p*(:) and **MM***q*(:,1) = **M** × **M***q*(:). Threads are created by the *parallel* directive. The directive *default(none)* specifies that each variable used in the parallel region must be declared as either private or shared. Reductions are performed over **MM***p* and **MM***q*. The *do schedule(static)* directive assigns loop values to different threads in a round-robin manner.

We used OpenMP to parallelize the most computationally intense parts of this TS-SCS algorithm. The loops parallelized included calculation of unscreened and screened total *C*_6_ for the unit cell (analogous to the loops parallelized in Fig. 1 and 5) and all of the large matrix-vector multiplies in the FCR algorithm. Fig. 8 shows an excerpt of the OpenMP enabled code for the large matrix-vector multiplies to compute **MM***p*(:,1) = **M** × **M***p*(:) and **MM***q*(:,1) = **M** × **M***q*(:).

The TS-SCS(FCR) code achieves linear-scaling computational cost for sufficiently large *N*atoms when the number of FCR iterations does not increase appreciably with increasing system size. To date, we have not observed any materials for which TS-SCS(FCR) converges slowly. However, the fixed number of required iterations for MCLF is a clear advantage compared to the variable number of iterations to converge TS-SCS. First, it makes MCLF convergence highly repeatable, which will become important for applications that involve differentiation with respect to atomic positions (*e.g.*, computing forces). Second, it makes MCLF computational times highly predictable, because the number of required iterations is known up front.

## 4. Performance results

### 4.1 Required computational time and memory

In addition to the linear-scaling MCLF and TS-SCS algorithms described in Sections 2 and 3 above, MCLF and TS-SCS were also programmed using direct matrix inversions *via* Gaussian elimination with partial pivoting (GEPP). GEPP is a widespread algorithm described in many numerical methods textbooks.^60^ This allowed us to compare both the computational time and precision (see Tables 4 and 5) of the inverse-free algorithms to the direct inverse algorithms.

Both the required computational time and memory of the inverse-free algorithms are proportional to the number of atoms in the unit cell times the number of separately cataloged pairwise interactions per atom. *Case 1*: for an isolated molecule much smaller than the dipole interaction cutoff length, increasing the number of atoms in the molecule also increases the number of pairwise interactions per atom. In this case, the required computational time and memory scale proportional to the number of atoms squared. *Case 2*: quadratic scaling of computational time and memory is also observed for periodic materials having small unit cells. As the number of atoms in the unit cell increases, the number of separately cataloged pairwise interactions per atom also increases. *Case 3*: when the unit cell is large enough to completely enclose a sphere of dipole interaction cutoff length radius, the number of separately cataloged pairwise interactions per atom saturates. Making the unit cell even larger does not increase the number of separately cataloged pairwise interactions per atom. In this case, both the required computational time and memory scale linearly with increasing system size.

**Fig. 9:**
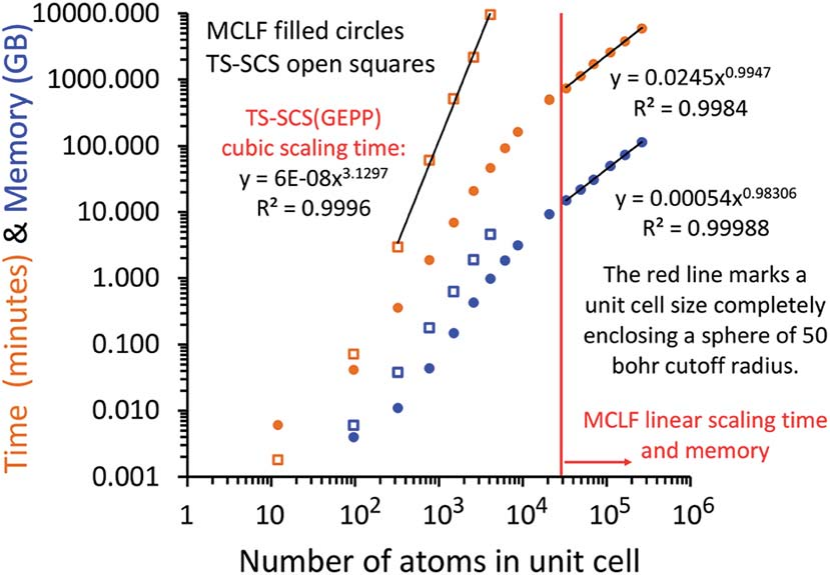
Plot of required computational time and random access memory (RAM) to perform MCLF analysis on ice crystals containing different numbers of atoms in the periodic unit cell: 12, 96, 324, 768, 1500, 2592, 4116, 6144, 8748, 20 736, 32 928, 49 152, 69 984, 111 132, 165 888, and 263 424. For 12 atoms, the required RAM was <1 MB. Beyond a certain system size (governed by the dipole interaction cutoff length), the required computational time and memory scale linearly with increasing system size. TS-SCS results using GEPP for 12 to 4116 atoms per unit cell are also shown for comparison. Because of direct matrix inversion, the TS-SCS method using GEPP has nearly cubic scaling computational cost, which makes it infeasible for large unit cells. Each calculation time is the average of three runs in serial mode.

Fig. 9 plots required computational time and RAM to perform MCLF analysis on ice crystals containing different numbers of atoms in the periodic unit cell. These calculations described the same hexagonal ice crystal structure, but with different sized unit cells. MCLF results from these different sized unit cells are numerically equivalent. Electron densities for the unit cells containing 12 to 8748 atoms were taken from ref. 18. Herein, we also constructed periodic unit cells containing 20 736 to 263 424 atoms from the computed DDEC6 AIM properties of the smaller unit cells; no DFT calculations on these large supercells were needed. As shown in Fig. 9, the required computational time and memory for MCLF analysis scaled linearly with increasing number of atoms in the unit cell when the unit cell was large enough to enclose a sphere of dipole interaction cutoff length radius. The TS-SCS method using direct matrix inversion (GEPP algorithm) is plotted for comparison, because the prior literature used a similar approach.^2^ As shown in Fig. 9, MCLF is less computationally expensive than TS-SCS using GEPP. While TS-SCS(GEPP) calculations larger than 4116 atoms per unit cell did not complete in one week on a single processor, an MCLF calculation with 263 424 atoms in the unit cell completed in 4.1 days on a single processor.

**Fig. 10:**
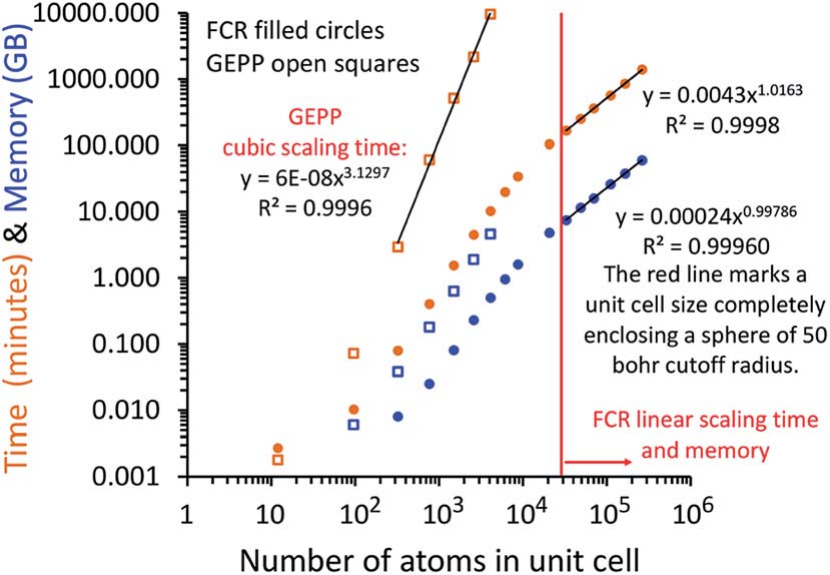
Comparison of failproof conjugate residual (FCR) and Gaussian elimination with partial pivoting (GEPP) algorithms for performing TS-SCS analysis. The same ice supercells were studied as listed in the caption of Fig. 9. The required RAM was <1 MB for 12 atoms (GEPP and FCR) and 96 atoms (FCR). Beyond a certain system size (governed by the dipole interaction cutoff length), the required computational time and memory for the FCR algorithm scale linearly with increasing system size. In contrast, the GEPP algorithm has nearly cubic scaling computational cost, which makes it infeasible for large unit cells. Each calculation time is the average of three runs in serial mode.

Fig. 10 compares the FCR and GEPP algorithms for performing TS-SCS analysis on these ice supercells. Direct matrix inversion using GEPP had nearly cubic scaling computational time with increasing number of atoms in the unit cell, which made it infeasible for unit cells containing >4116 atoms. Similar to MCLF analysis, the required computational time and memory for the TS-SCS(FCR) algorithm scaled linearly with increasing number of atoms in the unit cell when the unit cell was large enough to enclose a sphere of dipole interaction cutoff length radius. The TS-SCS(FCR) calculation with 263 424 atoms in the unit cell completed in 0.97 days on a single processor. TS-SCS(FCR) requires only about 50–60% of the memory as MCLF.

The computational cost of the GEPP algorithm for direct matrix inversion could be minimized by using a highly optimized linear algebra package such as LAPACK. This would result in faster computational times for GEPP than we reported here. However, this optimization would only lower the pre-factor and not the exponent in the required computational time scaling relation. Even for highly optimized code, the cubic-scaling computational time of GEPP makes it impractical for materials containing a large number of atoms in the unit cell.

Tables 7 and 8 list the calculation time breakdowns for MCLF and TS-SCS methods using the inverse-free algorithms. The computational times were consistent between runs, as demonstrated by the small standard deviations (<5%). For MCLF, the most time consuming section is the fluctuating screening, followed by static polarizability screening, and atom image pair matrix initialization. For TS-SCS, the most time consuming section is TS-SCS screening, followed by atom image pair matrix initialization. For both methods, the other sections take only negligible time. Fluctuations in file reading times were presumably due to variations in how quickly the files could be accessed, which depended on current file system load across users. The drop in computational time for unscreened *α* & *C*_6_ computation from 111 132 to 263 424 atoms is due to switching to the wp lookup table method for computing *C*_6_ with 263 424 atoms.

**Table 7:** Calculation time breakdown in seconds for MCLF analysis of ice crystals. Each time is the average of three runs. The standard deviation is based on the total time per run

Atoms in unit cell →	324	1500	4116	8748	20 736	49 152	111 132	263 424
Input file reading	0.01	0.3	0.4	0.1	0.2	0.8	1.6	3.3
Unscreened *α* & *C*_6_	0.1	0.1	0.3	0.9	3.4	16.6	79.5	12.8
Atom image pair matrix initialization	0.4	2.3	8.6	31.0	115.3	394.1	1174.0	2610.9
Non-directional screening	0.4	1.9	5.3	12.8	28.0	66.1	164.3	360.2
Fluctuating screening	16.2	329.7	2230.2	7878.4	23 807.5	54 089.5	123 403.6	281 602.7
Static *α* screening	4.1	82.4	557.3	1923.0	5929.8	13 226.3	29 717.1	69 835.3
Total	21.7	417.1	2798.0	9848.4	29 890.5	67 815.5	154 627.4	355 216.5
Standard deviation	0.9%	2.7%	0.1%	0.2%	0.5%	1.1%	1.4%	0.7%

**Table 8:** Calculation time breakdown in seconds for TS-SCS analysis of ice crystals. Each time is the average of three runs. The standard deviation is based on the total time per run

Atoms in unit cell →	324	1500	4116	8748	20 736	49 152	111 132	263 424
Input file reading	0.07	0.01	0.04	0.03	0.07	0.8	0.3	0.6
Unscreened *α* & *C*_6_	0.1	0.1	0.3	0.8	3.4	16.7	75.7	11.6
Atom image pair matrix initialization	0.2	1.7	6.8	25.1	106.1	365.1	1033.3	2534.6
TS-SCS screening	4.4	91.0	608.6	2027.7	6170.3	14 779.4	32 851.3	81 153.8
Total	4.8	92.7	615.8	2053.7	6279.9	15 162.0	33 960.5	83 700.7
Standard deviation	0.5%	2.3%	0.2%	1.6%	1.3%	0.4%	0.4%	2.9%

### 4.2 Parallelization efficiency

The parallelization efficiency is defined as (time for serial calculation)/((time for parallel calculation) × (number of parallel computing cores)). (Serial and parallel computational times were the average of three runs.) Tables 9 and 10 list the calculated efficiency of MCLF and TS-SCS methods for selected ice crystals. The parallelization efficiencies were excellent. Some jobs had efficiencies greater than 100%, because the parallel program uses OpenMP while the serial program does not. When enabled, OpenMP can speed up the calculation even if only one processor is used.

**Table 9:** Parallelization efficiency for MCLF analysis of ice crystals

Atoms in unit cell	Number of processors				
	1	2	4	8	16
324	113%	112%	107%	88%	73%
1500	113%	111%	109%	104%	100%
4116	108%	107%	105%	98%	103%
8748	121%	112%	117%	111%	114%
20 736	110%	118%	116%	107%	100%
49 152	111%	113%	112%	102%	109%
111 132	115%	105%	111%	106%	108%
263 424	112%	111%	111%	108%	104%

**Table 10:** Parallelization efficiency for TS-SCS analysis of ice crystals using FCR algorithm

Atoms in unit cell	Number of processors				
	1	2	4	8	16
324	98%	97%	93%	74%	58%
1500	103%	99%	97%	91%	89%
4116	97%	97%	96%	95%	94%
8748	101%	99%	97%	92%	95%
20 736	98%	98%	95%	98%	98%
49 152	100%	100%	93%	93%	96%
111 132	97%	100%	97%	96%	94%
263 424	101%	102%	101%	99%	97%

Table 11 compares the RAM requirements for serial execution to 8 parallel cores. Results are listed for both MCLF and TS-SCS(FCR) algorithms. Our results show that running the program in serial and parallel modes requires about the same amount of memory regardless of the number of cores used. In other words, adding parallel cores does not significantly increase the program's memory requirements.

**Table 11:** Comparison of RAM required in GB for serial and parallel program execution. Results are listed for both MCLF and TS-SCS(FCR) analysis of ice crystals

Atoms in unit cell	MCLF		TS-SCS(FCR)	
	Serial	8 core	Serial	8 core
324	0.011	0.012	0.008	0.008
1500	0.15	0.15	0.081	0.082
4116	0.98	0.98	0.50	0.53
8748	3.2	3.2	1.6	1.6
20 736	9.3	9.3	4.8	4.8
49 152	22	22	11.5	11.5
111 132	50	50	26	26
263 424	115	115	60	60

To quantify the performance of these algorithms for larger systems, an ice supercell containing >2 million atoms in the unit cell was prepared from the DDEC6 AIM properties. Specifically, a 2 × 2 × 2 supercell of the 263 424 atom unit cell gave a periodic supercell containing 2 107 392 atoms. Due to the large size, serial (*i.e.*, one processor) TS-SCS(FCR) and MCLF calculations could not complete in less than one week; therefore, only parallel calculations were run. Secondly, the source code had to be compiled using 64-bit integers to accommodate the large range of array index values. This 64-bit integer source code and a large memory node were also used for the 1 053 696 atom system reported in Table 3 above. (All other calculations reported in this paper used 32-bit integers, because this was the compiler's default.) Thirdly, the program had to run on a large memory node, because a normal node does not contain enough RAM to complete the calculation. The processor speed for the large memory node on the Comet cluster was 2.2 GHz compared to 2.5 GHz for its normal nodes. Therefore, one must exercise caution when comparing timing results for this large supercell to results reported above for the other ice supercells that ran on the normal nodes. The MCLF calculation for this material took 21.7 hours (average of 3 runs with standard deviation of 5%) on 48 parallel computing cores with 950 GB RAM. The TS-SCS(FCR) calculation took 5.6 hours (average of 3 runs with standard deviation of 2%) on 48 parallel computing cores with 500 GB RAM.

## 5. Conclusions

We developed computationally efficient algorithms to compute atom-in-material polarizabilities and dispersion coefficients using MCLF and TS-SCS analysis. Our MCLF algorithm uses Richardson extrapolation of the screening increments. Our TS-SCS algorithm uses a special conjugate residual algorithm that resists round-off errors. Both our algorithms have computational time and memory requirements scaling linearly with the number of atoms in the unit cell when the unit cell is much larger than the dipole interaction cutoff distance. For both small and large systems, our algorithms require less computational time and memory than direct matrix inversion, with negligible change in computational precision. Our algorithms achieved this by avoiding both large matrix inversions and large dense matrix multiplies. This is an important achievement, because direct matrix inversion and dense square matrix multiplication have computational costs scaling between *N*rows^2^ and *N*rows^3^.^11^ Other important algorithms to achieve linear scaling included: (a) classification of atoms into spatial regions followed by constructing two lists of interacting atom pairs and (b) a lookup table method to compute *C*^total^_6_.

Our algorithms were easily parallelized to take advantage of multiple computing cores. To minimize false sharing, our algorithms access data in cache line friendly order. Excellent parallelization efficiencies were obtained. Moreover, adding parallel computing cores did not significantly increase memory requirements.

This made it possible to apply the MCLF and TS-SCS methods to materials containing orders of magnitude more atoms per unit cell than previously feasible. Our algorithms can be readily applied to materials containing millions of atoms in the unit cell. The largest example studied herein was an ice crystal containing >2 million atoms in the unit cell. To perform TS-SCS on this material, the FCR algorithm solved a linear equation system containing >6 million rows, 7.57 billion interacting atom pairs in the large list and 87 million in the small list, 45.4 billion stored non-negligible matrix components used in each large matrix-vector multiplication, and ∼19 million unknowns per frequency point (>300 million total unknowns). This problem was solved in 5.6 hours by 48 parallel computing cores with 500 GB RAM. The MCLF calculation for this material took 21.7 hours on 48 parallel computing cores with 950 GB RAM.

The MCLF and TS-SCS software programs described here will be distributed through the same code repository as the Chargemol program. Required inputs for the TS-SCS program are: (i) an *xyz* file containing the list of atoms (as element symbols and *x*, *y*, *z* coordinates in Å), unit cell information (*i.e.*, lattice vectors if system is periodic), and AIM 〈*r*^3^〉 moments and (ii) a calculation_parameters.txt file listing calculation parameter values. Required inputs for the MCLF program are: (i) separate *xyz* files for AIM volumes, 〈*r*^3^〉 moments, 〈*r*^4^〉 moments, weighted 〈*r*^4^〉 moments, and net atomic charges, and (ii) a calculation_parameters.txt file listing calculation parameter values. Settings in the calculation_parameters.txt file choose whether to use an inverse-free method or direct matrix inversion, the Romberg integration order, the dipole interaction cutoff length, whether to ignore PBC, the number of Richardson extrapolation steps (for MCLF), the convergence threshold (for TS-SCS(FCR)), *etc.* The default values are reliable and almost never need to be changed.

Finally, the new failsafe conjugate residual algorithm should find widespread applications to a plethora of scientific computing problems, because it resists round-off errors and solves any linear equation system with Hermitian coefficients matrix. This conjugate residual algorithm has many desirable mathematical properties, because it minimizes the norm of the conditioned residual within a Krylov subspace of increasing order with each successive iteration.

## Authors' contributions

T. A. M. supervised the study, obtained funding, developed all computational and mathematical methods, generated and wrote all mathematical derivations and proofs (including entire FCR algorithm, wp lookup table method, atom image pair array initialization, Richardson extrapolation, and Romberg integration), wrote the entire ESI,† designed all computational tests, and wrote the MCLF and TS-SCS software codes. Both authors performed calculations, data analysis, and prepared tables and figures for the main text. The manuscript was written mainly by T. A. M. with minor contribution from T. C.

## Conflicts of interest

There are no conflicts of interest to declare.

## Supplementary Material

RA-009-C9RA01983A-s001

RA-009-C9RA01983A-s002

RA-009-C9RA01983A-s003

RA-009-C9RA01983A-s004

RA-009-C9RA01983A-s005

RA-009-C9RA01983A-s006

RA-009-C9RA01983A-s007

RA-009-C9RA01983A-s008
