# Supplementary material for: New scaling relations to compute atom-in-material polarizabilities and dispersion coefficients: part 2. Linear-scaling computational algorithms and parallelization
Source: RSC Adv. 2019 Oct 17;9(57):33310–36. doi: 10.1039/c9ra01983a (PMC9073276; doi:10.1039/c9ra01983a)
Supplement: RA-009-C9RA01983A-s001 [file RA-009-C9RA01983A-s001.pdf]

Electronic Supplementary Material (ESI) for RSC Advances.
This journal is © The Royal Society of Chemistry 2019

Electronic Supplementary Information for New scaling relations to compute atom-in-material polarizabilities and dispersion coefficients: part 2. Linear-scaling computational algorithms and parallelization

Thomas A. Manz[1]* and Taoyi Chen[1]

[1]Department of Chemical & Materials Engineering, New Mexico State University, Las Cruces, New Mexico, 88003-8001.

*Corresponding author email: tmanz@nmsu.edu

Contents

S1. Proof that $\alpha^{force\text{-}field}$, $\alpha^{non\text{-}dir}(u)$, α^{low_freq}, and $\alpha^{screened}(u)$ are ≥ 0

S2. Derivations and proofs related to the failproof conjugate residual (FCR) algorithm

S3. Proof the wp lookup table method yields error proportional to the increment squared

S4. Linear-scaling algorithm for setting up the lists of interacting atom pairs

S1. Proof that $\alpha^{force\text{-}field}$, $\alpha^{non\text{-}dir}(u)$, α^{low_freq}, and $\alpha^{screened}(u)$ are ≥ 0

Incremental non-directional screening takes the form

$$\alpha_{j+1}^{A}(u)=\alpha_{j}^{A}(u)-\sum_{b\in small\_list}\left[\left(\frac{2\alpha_{A}^{unscreened}(u)}{\alpha_{A}^{unscreened}(u)+\alpha_{B}^{unscreened}(u)}\right)\left(\alpha_{j}^{A}(u)\left(\Delta_{j}\tau_{non-dir,j}^{Ab}(u)\right)\alpha_{j}^{b}(u)\right)\right] \quad (S1)$$

where Δ_j is the screening increment. Eqn (S1) can be factored to give

$$\alpha_{j+1}^{A}(u)=\alpha_{j}^{A}(u)\left(1-\sum_{b\in small\_list}\left(\frac{2\alpha_{A}^{unscreened}(u)\left(\Delta_{j}\tau_{non-dir,j}^{Ab}(u)\right)\alpha_{j}^{b}(u)}{\alpha_{A}^{unscreened}(u)+\alpha_{B}^{unscreened}(u)}\right)\right) \quad (S2)$$

Since the MCLF method corresponds to the limit $\Delta_j \to 0$, we have

$$\lim_{\Delta_j\to 0}\left(1-\sum_{b\in small\_list}\left(\frac{2\alpha_{A}^{unscreened}(u)\left(\Delta_{j}\tau_{non-dir,j}^{Ab}(u)\right)\alpha_{j}^{b}(u)}{\alpha_{A}^{unscreened}(u)+\alpha_{B}^{unscreened}(u)}\right)\right)>0 \quad (S3)$$

Combining eqn (S2) and (S3) gives

$$\lim_{\Delta_j\to 0}\left(\frac{\alpha_{j+1}^{A}(u)}{\alpha_{j}^{A}(u)}\right)>0 \quad (S4)$$

Hence, $\alpha_{j+1}^{A}(u)$ and $\alpha_{j}^{A}(u)$ have the same sign. Since $\alpha_{j=1}^{A}(u)=\alpha_{A}^{unscreened}(u)\geq 0$, it follows that $\alpha_{j}^{A}(u)\geq 0$ for all values of j. Since $\alpha_{A}^{non-dir}(u)$ corresponds to the final value of j in the limit $\Delta_j \to 0$, it necessarily follows that

$$\alpha_{A}^{non-dir}(u)\geq 0 \quad (S5)$$

Since $\alpha_{A}^{force-field}=\alpha_{A}^{non-dir}(u=\text{Nimfreqs})$, then $\alpha_{A}^{force-field}\geq 0$.

Incremental directional screening to compute $\alpha_{A}^{screened}(u)$ takes the form

$$M1=\left(\Delta_{j}f_{MBS}^{Ab}f_{cutoff}^{Ab}\varphi_{j}^{Ab}(u)\right)\left(\vec{\vec{\alpha}}_{j}^{A}(u)\bullet\left(\vec{\vec{\eta}}^{Ab}(u)\right)\bullet\vec{\vec{\alpha}}_{j}^{b}(u)\right) \quad (S6)$$

$$M2=\Delta_{j}\vec{\vec{\alpha}}_{j}^{A}(u)\bullet\vec{\vec{h}}^{AB}(u)\bullet\vec{\vec{\alpha}}_{j}^{B}(u) \quad (S7)$$

$$\begin{aligned}\vec{\vec{\alpha}}_{j+1}^{A}(u)=\vec{\vec{\alpha}}_{j}^{A}(u)&-\sum_{b\in small\_list}\left[\left(\frac{\alpha_{A}^{non-dir}(u)}{\alpha_{A}^{non-dir}(u)+\alpha_{B}^{non-dir}(u)}\right)\left(M1+M1^{T}\right)\right]\\&-\sum_{B\in large\_list}\left[\left(\frac{\alpha_{A}^{non-dir}(u)}{\alpha_{A}^{non-dir}(u)+\alpha_{B}^{non-dir}(u)}\right)\left(M2+M2^{T}\right)\right]\end{aligned} \quad (S8)$$

where M1 and M2 are square matrices with 3 rows and

$$\varphi_{j}^{Ab}(u)=-\left(\frac{1}{(d_{Ab})^{3}}\text{erfc}\left(\frac{d_{Ab}}{\sigma_{AB,j}(u)}\right)+\left(\frac{2}{\sqrt{\pi}}\right)\left(\frac{1}{(d_{Ab})^{2}\sigma_{AB,j}(u)}+\frac{2}{3\left(\sigma_{AB,j}(u)\right)^{3}}\right)\exp\left(-\left(\frac{d_{Ab}}{\sigma_{AB,j}(u)}\right)^{2}\right)\right)$$

(S9)

$$\alpha_j^A(u) = \text{trace}\left(\vec{\alpha}_j^A(u)\right)/3 \quad \text{(S10)}$$

Eqn (S8) can be factored to give

$$\vec{\alpha}_{j+1}^A(u) = ½\vec{\alpha}_j^A(u)\left(I - \Delta_j M3\right) + ½\left(I - \Delta_j M3\right)\vec{\alpha}_j^A(u) = \vec{\alpha}_j^A(u) - ½\Delta_j\left(\underbrace{\vec{\alpha}_j^A(u)M3 + M3\vec{\alpha}_j^A(u)}_{\text{symmetric}}\right)$$

(S11)

where M3 is some finite matrix. Since M3 remains finite, then in the limit $\Delta_j \to 0$ the eigenvalues of $\left(I - \Delta_j M3\right)$ must approach 1. Since the determinant of a matrix is the product of its eigenvalues,

$$\det\left(I - \Delta_j M3\right) > 0 \quad \text{(S12)}$$

for sufficiently small finite Δ_j. Since the determinant of the product of two matrices is the product of their determinants, then from eqn (S12) it follows that

$$\det\left(\vec{\alpha}_j^A(u)\left(I - \Delta_j M3\right)\right) = \det\left(\left(I - \Delta_j M3\right)\vec{\alpha}_j^A(u)\right) = \det\left(I - \Delta_j M3\right)\det\left(\vec{\alpha}_j^A(u)\right) \quad \text{(S13)}$$

has the same sign as $\det\left(\vec{\alpha}_j^A(u)\right)$. Hence, for sufficiently small finite Δ_j, the eigenvalues of $\vec{\alpha}_j^A(u)\left(I - \Delta_j M3\right)$ and $\left(I - \Delta_j M3\right)\vec{\alpha}_j^A(u)$ must have the same signs as the eigenvalues of $\vec{\alpha}_j^A(u)$. Since $\det\left(I - \Delta_j M3\right)$ is not close to zero for sufficiently small finite Δ_j (eqn (S12)), then none of the eigenvalues of $\vec{\alpha}_j^A(u)\left(I - \Delta_j M3\right)$ and $\left(I - \Delta_j M3\right)\vec{\alpha}_j^A(u)$ can be zero if none of the eigenvalues of $\vec{\alpha}_j^A(u)$ are zero. By definition, a real-valued matrix M is positive definite if and only if

$$z^T M z > 0 \quad \text{(S14)}$$

for every non-zero vector z. A real-valued matrix is positive definite if and only if its symmetric part has all positive eigenvalues.[1] Taken together, the above results show that when $\vec{\alpha}_j^A(u)$ is positive definite, then $\vec{\alpha}_j^A(u)\left(I - \Delta_j M3\right)$ and $\left(I - \Delta_j M3\right)\vec{\alpha}_j^A(u)$ are also positive definite for sufficiently small finite Δ_j. The sum of two positive definite matrices $M = M' + M''$ is always positive definite:

$$z^T\left(M' + M''\right)z = \underbrace{z^T M' z}_{>0} + \underbrace{z^T M'' z}_{>0} > 0 \quad \text{(S15)}$$

Since $\vec{\alpha}_{j+1}^A(u)$ is one-half the sum of $\vec{\alpha}_j^A(u)\left(I - \Delta_j M3\right)$ and $\left(I - \Delta_j M3\right)\vec{\alpha}_j^A(u)$, it follows that $\vec{\alpha}_{j+1}^A(u)$ is positive definite if $\vec{\alpha}_j^A(u)\left(I - \Delta_j M3\right)$ and $\left(I - \Delta_j M3\right)\vec{\alpha}_j^A(u)$ are positive definite. Therefore, $\vec{\alpha}_{j+1}^A(u)$ is positive definite when $\vec{\alpha}_j^A(u)$ is positive definite for sufficiently small finite Δ_j.

The directional screening process starts with

$$\vec{\alpha}_{j=1}^{A}(u)=\begin{bmatrix}\alpha_{A}^{non-dir}(u) & 0 & 0\\ 0 & \alpha_{A}^{non-dir}(u) & 0\\ 0 & 0 & \alpha_{A}^{non-dir}(u)\end{bmatrix} \quad \text{(S16)}$$

and ends with $\alpha_{A}^{screened}(u)$ as one-third the trace of the tensor on the left-side of eqn (S8) after the last screening increment finishes. Therefore,

$$\det\left(\vec{\alpha}_{j=1}^{A}(u)\right)=\left(\alpha_{A}^{non-dir}(u)\right)^{3}\geq 0 \quad \text{(S17)}$$

and the eigenvalues of $\vec{\alpha}_{j=1}^{A}(u)$ are $\alpha_{A}^{non-dir}(u)\geq 0$. There are two cases. *Case 1*: If $\alpha_{A}^{non-dir}(u)=0$, then all eigenvalues of $\vec{\alpha}_{j=1}^{A}(u)$ are zero, and by eqn (S11) the limit $\Delta_{j}\to 0$ gives $\vec{\alpha}_{j+1}^{A}(u)=0$ and hence $\alpha_{A}^{screened}(u)=0$. *Case 2*: If $\alpha_{A}^{non-dir}(u)>0$, then all eigenvalues of $\vec{\alpha}_{j=1}^{A}(u)$ are > 0 (i.e., $\vec{\alpha}_{j=1}^{A}(u)$ is positive definite), and by the analysis of the prior paragraph, $\vec{\alpha}_{j+1}^{A}(u)$ is also positive definite for sufficiently small finite Δ_{j}. Since the trace is the sum of eigenvalues, all positive eigenvalues yields positive trace. Thus,

$$\alpha_{A}^{screened}(u)\geq 0 \quad \text{(S18)}$$

in all cases. Since $\alpha_{A}^{low\_freq}=\alpha_{A}^{screened}(u=\text{Nimfreqs})$, then $\alpha_{A}^{low\_freq}\geq 0$.

S2. Derivations and proofs related to the failproof conjugate residual (FCR) algorithm
S2.1 Problem definition and matrix conditioning

FCR solves any linear equation system with Hermitian coefficients matrix

$$\mathcal{A}\mathcal{x}=\mathcal{B} \quad \text{(S19)}$$

for an exact solution (within the convergence tolerance) if one exists or for a conditioned least-squares solution if no exact solution exists. The matrices $\mathcal{x}$ and $\mathcal{B}$ may contain a single column or more than one column. Matrix $\mathcal{A}$ is non-singular if and only if its determinant is nonzero (i.e., all of its eigenvalues are non-zero). In this case, matrix $\mathcal{A}$ is invertible and the equation $\mathcal{A}\mathcal{x}=\mathcal{B}$ has the unique solution $\mathcal{x}=\mathcal{A}^{-1}\mathcal{B}$. If matrix $\mathcal{A}$ is singular, then $\mathcal{A}\mathcal{x}=\mathcal{B}$ has either no solution or an infinite number of solutions. If the linear equation system is consistent (i.e., has at least one solution), this FCR algorithm returns one of its solutions. When matrix $\mathcal{B}$ has more than one column, the FCR algorithm is applied separately to each column. When $\mathcal{B}$ = I (identity matrix), the method solves for $\mathcal{x}=\mathcal{A}^{-1}$, the inverse of matrix $\mathcal{A}$.

The goal of conditioning is to rotate and scale matrix $\mathcal{A}$ to improve convergence speed:

$$M=C\mathcal{A}C^{H} \quad \text{(S20)}$$

$$My=C\mathcal{A}C^{H}y=C\left(\mathcal{B}-\mathcal{A}\mathcal{x}_{0}\right)=W \quad \text{(S21)}$$

where

$$\mathcal{x}=C^{H}y+\mathcal{x}_{0} \quad \text{(S22)}$$

Here, $\mathcal{x}_0$ is any initial guess for $\mathcal{x}$. This shift always makes y_0=0 as the initial guess for y. The conditioning matrix C must be non-singular. Since $\mathcal{A}$ is Hermitian, the matrix M will automatically be Hermitian for any conditioning matrix C:

$$M^H = \left(C\mathcal{A}C^H\right)^H = \left(C^H\right)^H \mathcal{A}^H C^H = C\mathcal{A}C^H = M \quad \text{(S23)}$$

As shown in Section 2.2 below, the algorithm's convergence speed is largely affected by matrix M's eigenspectrum. As with other conjugate gradient methods, convergence is most rapid if the eigenvalues are clustered together.[2] Thus, conditioning's primary goal is to make the eigenspectrum more clustered together.[2]

In this article, the dot project of two vectors v and w is defined as

$$\langle v|w\rangle = v^H w = \left(v^*\right)^T w = \sum_i v_i^* w_i \quad \text{(S24)}$$

A Hermitian matrix M = M^H can be freely moved between sides of the dot product. For example,

$$\langle MMq|p\rangle = \langle Mq|Mp\rangle = \langle q|MMp\rangle = q^H M^2 p \quad \text{(S25)}$$

As commonly known, the Hermitian conjugate of a matrix product follows

$$\left(\Upsilon\Lambda\right)^H = \Lambda^H \Upsilon^H \quad \text{(S26)}$$

If the linear equation system (i.e., eqn (S19)) is inconsistent (i.e., has no exact solution), this FCR algorithm returns a statement that the linear equation system has no exact solution along with a value y = y^{FCR} that minimizes the least-squares problem

$$\text{Minimize} \quad F = \langle z|z\rangle = \left|W - My\right|^2 \quad \text{(S27)}$$

This represents a best possible choice for y irrespective of whether an exact solution to eqn (S19) exists. There are some applications where this least-squares fit has utility even if an exact solution to eqn (S19) does not exist. (Note: The case where My=W is consistent arises as the special case of eqn (S27) where the least-squares error is simply zero.)

As commonly defined, the kernel of M is the set of all y values that solve My=0,

$$\text{kernel}\left(M\right) \Rightarrow My = 0 \quad \text{(S28)}$$

When M is non-singular, y=0 is the only vector in the kernel. When M is singular, the kernel includes y=0 along with an infinite number of non-zero vectors. Manifestly, any vector from the kernel of M can be added to y^{FCR} without changing the conditioned residual value. Therefore, the set of all equally good (aka 'best') solutions to eqn (S27) is

$$y^{all} = y^{FCR} + \text{span}\left[\text{kernel}\left(M\right)\right] \quad \text{(S29)}$$

If the kernel(M) is known, this allows calculating the entire set of $\left\{y^{all}\right\}$ that give the same minimum value of F. If My=W is consistent, this is the set of y values yielding F=0 (i.e., satisfying eqn (S21)). The number of vectors in $\left\{y^{all}\right\}$ is one if M is non-singular; otherwise, $\left\{y^{all}\right\}$ contains an infinite number of vectors.

S2.2 Eigenvalue decomposition analysis using Krylov subspaces

S2.2.1 Mathematical formalism

The method optimizes the conditioned residuals

$$z^{(i)} = W - My^{(i)} = C\,r^{(i)} = C\left(B - \mathcal{A}x^{(i)}\right) \tag{S30}$$

where enclosed subscripts refer to the iteration number. A Krylov subspace is defined as

$$K_n\left(M, W\right) = \mathrm{span}\left[W, MW,\ M^2W, M^3W,\ \ldots M^{n-1}W\right] \tag{S31}$$

In each iteration, $y^{(i)}$ is chosen from the Krylov subspace

$$y^{(i)} \in K_{2i}\left(M, W\right) \tag{S32}$$

to minimize the norm

$$\Gamma^{(i)} = \left\langle z^{(i)} \middle| z^{(i)} \right\rangle \tag{S33}$$

Since $z^{(0)} = W$, from eqn (S30) – (S32) it follows that

$$z^{(i)} \in K_{2i+1}\left(M, W\right) \tag{S34}$$

Convergence analysis can be obtained by expanding W in terms of the eigenvectors $\left\{\vec{V}_j\right\}$ of M:

$$W = \sum_j c_j \vec{V}_j \tag{S35}$$

The building blocks of the Krylov subspace are thus

$$M^k W = \sum_j c_j \left(\lambda_j\right)^k \vec{V}_j \tag{S36}$$

where $\left\{\lambda_j\right\}$ are the corresponding eigenvalues of M. These building blocks can generate at most Ξ linearly independent vectors, where W is a linear combination of eigenvectors of M having exactly Ξ distinct eigenvalues λ_j.

FCR expands $y^{(i)}$ as

$$y^{(i)} = a_0 W + a_1 MW + \ldots a_{2i-1} M^{2i-1} W = \sum_j c_j \mathrm{Poly}^{(i)}\left(\lambda_j\right)\vec{V}_j \tag{S37}$$

$$My^{(i)} = a_0 MW + a_1 M^2 W + \ldots a_{2i-1} M^{2i} W = \sum_j c_j \lambda_j \mathrm{Poly}^{(i)}\left(\lambda_j\right)\vec{V}_j \tag{S38}$$

$$z^{(i)} = W - My^{(i)} = \sum_j c_j \vec{V}_j - \sum_j c_j \lambda_j \mathrm{Poly}^{(i)}\left(\lambda_j\right)\vec{V}_j = \sum_j c_j \vec{V}_j \left(1 - \lambda_j \mathrm{Poly}^{(i)}\left(\lambda_j\right)\right) \tag{S39}$$

where $\left\{a_k\right\}$ are some optimized coefficients and

$$\mathrm{Poly}^{(i)}\left(\lambda\right) = \sum_{k=0}^{2i-1} a_k \left(\lambda\right)^k \tag{S40}$$

is the associated polynomial. Examining eqn (S39), an exact solution is reached if

$$\mathrm{Poly}^{(i)}\left(\lambda_j\right) \Rightarrow 1/\lambda_j \tag{S41}$$

The FCR convergence properties are thus dictated by the difficulty of representing $\{1/\lambda_j\}$ via a polynomial $\mathrm{Poly}^{(i)}(\lambda)$. When the eigenvalues are close to one, this is trivial because

$$\frac{1}{1+\underbrace{\left(\lambda_j-1\right)}_{\varepsilon_j}}=1-\varepsilon_j+\varepsilon_j{}^2-\varepsilon_j{}^3+\varepsilon_j{}^4\ldots \quad \text{(S42)}$$

When the eigenvalues are extremely spread out in values, then it takes a higher-order polynomial (and hence a larger number of FCR iterations) to make $\mathrm{Poly}^{(i)}(\lambda)\approx 1/\lambda$ for all of the eigenvalues contributing to W (eqn (S35)). Thus, the primary goal of conditioning (eqn (S20)) is to make the eigenvalues less spread out in values.

Combining eqn (S31) and (S35) means the Krylov subspaces can be expanded in terms of eigenvalues as

$$K_n\left(M,W\right)=\mathrm{span}\left[\sum_j c_j\vec{V}_j\sum_{k=0}^{n-1}b_k\left(\lambda_j\right)^k\right] \quad \text{(S43)}$$

where span refers to the space generated by all possible $\{b_k\}$ values.

S2.2.2 Non-singular coefficients matrix

The matrix M is non-singular if and only if all of its eigenvalues are non-zero: $\lambda_j\neq 0$ for every j. Every non-singular matrix M is invertible. Therefore, the linear equation system My=W is consistent and has the unique solution: y = M^{-1}W.

For non-singular matrices in exact arithmetic, FCR converges to the exact solution in at most ceiling(Ξ/2) iterations. This follows immediately from eqn (S37) and (S41). Specifically, the exact solution occurs when $\mathrm{Poly}^{(i)}(\lambda_j)=1/\lambda_j$ for j = 1, 2, … Ξ distinct eigenvalues. A polynomial containing Ξ coefficients (e.g., a_0, a_1, … $a_{(\Xi-1)}$) can obviously be fit to pass through exactly Ξ points. By eqn (S37), the polynomial contains 2i coefficients, where i is the number of FCR iterations. Therefore, in exact arithmetic FCR converges to the exact solution in at most ceiling(Ξ/2) iterations. Because the polynomial adds two new coefficients in each FCR iteration, we say each FCR iteration searches 2 independent directions. Note that $\Xi\leq \mathrm{Nrows}$, where Nrows is the number of rows in matrix M. For example, if matrix M contains Nrows =1 million, but matrix M has only 12 distinct eigenvalues, then FCR will converge to the exact result in at most 6 iterations in exact arithmetic.

S2.2.3 Singular coefficients matrix

Matrix M is singular if and only if one of its distinct eigenvalues equals zero. Singular matrices are not invertible. Let $\lambda^\circ=0$ be the particular eigenvalue that is zero. Let c° be the coefficient in eqn (S35) associated with this particular eigenvalue. If $c^\circ\neq 0$, then let V° be the eigenvector (associated with zero eigenvalue λ°) that makes non-zero contribution to W in eqn (S35).

The eigenvectors of a Hermitian matrix can always be chosen to form an orthonormal basis that spans the whole Nrows-dimensional space. Consequently, for singular matrices the eigenvector(s) spanning kernel(M) are orthogonal to all eigenvectors not in kernel(M).

For singular matrices, there are obviously two separate cases. _Case 1_: When $c^\circ = 0$, then the zero-eigenvalue of M does not contribute anything to the vector W. In this case, eigenvector(s) $\{V^\circ\}$ associated with zero eigenvalue do not appear in the Krylov subspace sequence is built-up as W, MW, M^2W, …. Since the conjugate residual method is based on this Krylov subspace sequence, the eigenstate(s) $\{(\lambda^\circ, V^\circ)\}$ are completely irrelevant to the problem in exact arithmetic, because these zero eigenstates simply do not appear in the equation to be solved nor in its solution. In this case, the linear equation system My=W is consistent and its solution in exact arithmetic proceeds analogously to the non-singular case.

Case 2: When $c^\circ \neq 0$, then a zero eigenstate makes non-zero contribution to W in eqn (S35). Examining eqn (S39), an exact solution would be reached if

$$1 - \lambda_j \mathrm{Poly}^{(i)}(\lambda_j) = 0 \tag{S44}$$

for every eigenstate contributing to W. For $\lambda^\circ = 0$, eqn (S44) simplifies to

$$1 = 0$$

which is manifestly inconsistent. Therefore, the linear equation system My=W is manifestly inconsistent in this case. Because My multiplies each eigenvector contributing to y by its eigenvalue, then My does not contain any contributions from zero eigenstates. Therefore, whenever W contains a non-zero contribution from a zero eigenstate, no exact solution exists.

For singular coefficients matrix (whether the linear equation system is consistent or inconsistent), each iteration removes conditioned residual along two z-search directions that are orthogonal to each other and all prior z-search directions. Therefore, convergence is reached in at most ceiling(Ξ/2) iterations, which is the same result as for the non-singular case given in Section S2.2.2 above.

S2.2.4 Deriving the convergence criteria

Regardless of whether the linear equation system is non-singular consistent, singular consistent, or singular inconsistent, the least-squares solution corresponds to removing all contributions to W that are not in kernel(M), with the final residual being that portion of W that lies within kernel(M):

$$z^{\min} = W \cap \mathrm{kernel}(M) \tag{S45}$$

$$\Gamma^{\min} = \langle z^{\min} | z^{\min} \rangle = \min |W - My|^2 \tag{S46}$$

Examining eqn (S39), this FCR method always proceeds normally along all z-search directions that are not in kernel(M). Because these z-search directions are a linear combination of eigenstates that are orthogonal to kernel(M), projection of the conditioned residual onto kernel(M) is not effected during this search.

Manifestly, $\left\langle Mz^{(i)} \middle| Mz^{(i)} \right\rangle = 0$ if only if $z^{(i)} \in \text{kernel}(M)$, in which case $z^{(i)} = z^{\min}$ and the algorithm is converged. Therefore, a needed condition to construct a fail-proof conjugate residual method is that one of the z-search directions (say $Mp^{(i)}$) is chosen such that

$$\left\langle Mp^{(i)} \middle| z^{(i-1)} \right\rangle = \left\langle Mz^{(i-1)} \middle| Mz^{(i-1)} \right\rangle \geq 0 \tag{S47}$$

so that z-search always reduces the conditioned residual norm in each and every iteration until convergence. If $\left\langle Mp^{(i)} \middle| Mp^{(i)} \right\rangle = 0$ then $p^{(i)} \in \text{kernel}(M)$ and hence $\left\langle Mp^{(i)} \middle| z^{(i-1)} \right\rangle = \left\langle Mz^{(i-1)} \middle| Mz^{(i-1)} \right\rangle = 0$. Therefore, if $\left\langle Mz^{(i-1)} \middle| Mz^{(i-1)} \right\rangle > 0$ then $\left\langle Mp^{(i)} \middle| Mp^{(i)} \right\rangle > 0$.

Therefore, the FCR algorithm has two convergence criteria. If $z^{(i)} \Rightarrow 0$, the FCR algorithm converged to an exact solution (within the convergence tolerance). If $z^{(i)} \not\approx 0$ but $\left\langle Mz^{(i)} \middle| Mz^{(i)} \right\rangle \Rightarrow 0$, then the linear equation system is inconsistent and the FCR algorithm converged to the least-squares solution (within the convergence tolerance).

S2.3 Defining conjugate search directions that span the Krylov subspaces

Each iteration involves two search directions, $p^{(i)}$ and $q^{(i)}$:

$$y^{(i)} = y^{(i-1)} + \gamma^{(i)} p^{(i)} + \tau^{(i)} q^{(i)} \tag{S48}$$

$$z^{(i)} = z^{(i-1)} - \gamma^{(i)} Mp^{(i)} - \tau^{(i)} Mq^{(i)} \tag{S49}$$

subject to the constraints

$$p^{(i)}, q^{(i)} \in K_{2i}(M, W) \tag{S50}$$

$$K_{2i}(M, W) = \text{span}\left[p^{(1)}, q^{(1)}, \ldots p^{(i)}, q^{(i)} \right] \tag{S51}$$

In exact arithmetic, all z-search directions are chosen to be orthogonal, thus ensuring a conjugate method:

$$\left\langle Mq^{(i)} \middle| Mq^{(j\neq i)} \right\rangle = \left\langle Mp^{(i)} \middle| Mq^{(j)} \right\rangle = \left\langle Mp^{(i)} \middle| Mp^{(j\neq i)} \right\rangle = 0 \tag{S52}$$

The conditioned residual component along z-search directions $Mp^{(i)}$ and $Mq^{(i)}$ are removed in iteration i. Since all other z-search directions are orthogonal to $Mp^{(i)}$ and $Mq^{(i)}$, no conditioned residual (except for round-off errors) is subsequently contaminated along these directions. Hence,

$$\left\langle Mq^{(i)} \middle| z^{(j\geq i)} \right\rangle = \left\langle Mp^{(i)} \middle| z^{(j\geq i)} \right\rangle = 0 \tag{S53}$$

Let $S^{(i)}$ be the new z-search space for iteration i. Then,

$$S^{(i)} = \text{span}\left[Mp^{(i)}, Mq^{(i)} \right] \tag{S54}$$

Following eqn (S53) and (S54), the conditioned residual is perpendicular to the current and prior search subspaces:

$$z^{(j\geq i)} \perp \text{span}\left[S^{(1)}, S^{(2)}, \ldots S^{(i)} \right] \tag{S55}$$

Because two conjugate search directions are chosen from a Krylov subspace that increases by two directions per iteration (and are orthogonal to all prior z-search directions), it follows that

$$\mathrm{span}\left[S^{(1)},S^{(2)},\ldots S^{(i+k)}\right]=\mathrm{span}\left[MW,M^{2}W,\ldots M^{2i+2k}W\right] \tag{S56}$$

$$M^{2k>0}p^{(i)},M^{1+2k>0}p^{(i)},M^{2k>0}q^{(i)},M^{1+2k>0}q^{(i)}\in\mathrm{span}\left[S^{(1)},S^{(2)},\ldots S^{(i+k)}\right] \tag{S57}$$

Similarly,

$$Mz^{(i)},M^{2}z^{(i)}\in\mathrm{span}\left[S^{(1)},S^{(2)},\ldots S^{(i+1)}\right] \tag{S58}$$

Thus,

$$\left\langle z^{(j\ge i+1)}\middle|Mz^{(i)}\right\rangle=\left\langle z^{(j\ge i+1)}\middle|M^{2}z^{(i)}\right\rangle=0 \tag{S59}$$

Combining eqn (S55) and (S57) yields:

$$\left\langle z^{(j\ge i+k)}\middle|M^{2k>0}p^{(i)}\right\rangle=\left\langle z^{(j\ge i+k)}\middle|M^{1+2k>0}p^{(i)}\right\rangle=\left\langle z^{(j\ge i+k)}\middle|M^{2k>0}q^{(i)}\right\rangle=\left\langle z^{(j\ge i+k)}\middle|M^{1+2k>0}q^{(i)}\right\rangle=0 \tag{S60}$$

Combining eqn (S49), (S52), and (S53) yields

$$\gamma^{(i)}=\frac{\left\langle Mp^{(i)}\middle|z^{(i-1)}\right\rangle}{\left\langle Mp^{(i)}\middle|Mp^{(i)}\right\rangle} \tag{S61}$$

$$\tau^{(i)}=\frac{\left\langle Mq^{(i)}\middle|z^{(i-1)}\right\rangle}{\left\langle Mq^{(i)}\middle|Mq^{(i)}\right\rangle} \tag{S62}$$

As stated above in eqn (S51) – (S52), $p^{(i)},q^{(i)}$ are M^2-conjugate to all prior search directions that span the Krylov subspaces of orders ≤2i-2. Since

$$M^{j\ge 0}\left(p,q\right)^{(i)}\in K_{2i+j} \tag{S63}$$

it follows that for j≥0:

$$\left\langle \left(p,q\right)^{(k>i+(j/2))}\middle|M^{2+j}\left(p,q\right)^{(i)}\right\rangle=0 \tag{S64}$$

where $\left(p,q\right)$ means the equation holds for both p and q (i.e., $M^{j\ge 0}\left(p,q\right)^{(i)}$ means it holds for $M^{j\ge 0}p^{(i)}$ and $M^{j\ge 0}q^{(i)}$). These equations are for exact arithmetic.

These requirements will be fulfilled by choosing

$$p^{(i)}=Mz^{(i-1)}-\sum_{j=1}^{i-1}\beta_{i-j}^{(i)}p^{(j)}-\sum_{j=1}^{i-1}\xi_{i-j}^{(i)}q^{(j)} \tag{S65}$$

First, we derive the conjugacy conditions. Contracting eqn (S65) with $M^{2}p^{(k<i)}$ gives

$$\left\langle M^{2}p^{(k)}\middle|p^{(i)}\right\rangle=\left\langle M^{2}p^{(k)}\middle|Mz^{(i-1)}\right\rangle-\sum_{j=1}^{i-1}\beta_{i-j}^{(i)}\left\langle M^{2}p^{(k)}\middle|p^{(j)}\right\rangle-\sum_{j=1}^{i-1}\xi_{i-j}^{(i)}\underbrace{\left\langle M^{2}p^{(k)}\middle|q^{(j)}\right\rangle}_{0}\Rightarrow 0 \tag{S66}$$

which must equal zero to satisfy eqn (S52). By eqn (S60),

$$\left\langle M^{2}p^{(k<i-1)}\middle|Mz^{(i-1)}\right\rangle=\left\langle M^{2}q^{(k<i-1)}\middle|Mz^{(i-1)}\right\rangle=0 \tag{S67}$$

which gives

$$\beta^{(i)}_{n\geq 2} = 0 \tag{S68}$$

$$\beta^{(i)} = \beta^{(i)}_1 = \frac{\left\langle M^2 p^{(i-1)} \middle| Mz^{(i-1)} \right\rangle}{\left\langle Mp^{(i-1)} \middle| Mp^{(i-1)} \right\rangle} \tag{S69}$$

Since $\beta^{(i)}_1$ is the only non-zero component, for convenience the subscript is dropped. Contracting eqn (S65) with $M^2 q^{(k<i)}$ gives

$$\left\langle M^2 q^{(k)} \middle| p^{(i)} \right\rangle = \left\langle M^2 q^{(k)} \middle| Mz^{(i-1)} \right\rangle - \sum_{j=1}^{i-1} \beta^{(i)}_{i-j} \underbrace{\left\langle M^2 q^{(k)} \middle| p^{(j)} \right\rangle}_{0} - \sum_{j=1}^{i-1} \xi^{(i)}_{i-j} \left\langle M^2 q^{(k)} \middle| q^{(j)} \right\rangle \Rightarrow 0 \tag{S70}$$

Making use of eqn (S67) gives

$$\xi^{(i)}_{n\geq 2} = 0 \tag{S71}$$

$$\xi^{(i)} = \xi^{(i)}_1 = \frac{\left\langle M^2 q^{(i-1)} \middle| Mz^{(i-1)} \right\rangle}{\left\langle Mq^{(i-1)} \middle| Mq^{(i-1)} \right\rangle} \tag{S72}$$

Hence,

$$p^{(i)} = Mz^{(i-1)} - \beta^{(i)} p^{(i-1)} - \xi^{(i)} q^{(i-1)} \tag{S73}$$

This choice for $p^{(i)}$ satisfies the requirement in eqn (S47) above:

$$\left\langle p^{(i)} \middle| Mz^{(i-1)} \right\rangle = \left\langle Mz^{(i-1)} \middle| Mz^{(i-1)} \right\rangle - \beta^{(i)} \left\langle p^{(i-1)} \middle| Mz^{(i-1)} \right\rangle - \xi^{(i)} \left\langle q^{(i-1)} \middle| Mz^{(i-1)} \right\rangle \tag{S74}$$

which rearranges to give

$$\left\langle Mp^{(i)} \middle| z^{(i-1)} \right\rangle = \left\langle Mz^{(i-1)} \middle| Mz^{(i-1)} \right\rangle - \beta^{(i)} \underbrace{\left\langle Mp^{(i-1)} \middle| z^{(i-1)} \right\rangle}_{0} - \xi^{(i)} \underbrace{\left\langle Mq^{(i-1)} \middle| z^{(i-1)} \right\rangle}_{0} = \left\langle Mz^{(i-1)} \middle| Mz^{(i-1)} \right\rangle \tag{S75}$$

Next, a method is derived to compute $q^{(i)}$. Since

$$K_{2i}\left(M, W\right) = \text{span}\left[W, MW, M^2 p^{(1)}, M^2 q^{(1)}, \ldots M^2 p^{(i-1)}, M^2 q^{(i-1)}\right] \tag{S76}$$

it follows that $M^2 q^{(i-1)}$ and $M^2 p^{(i-1)}$ are linearly independent of $K_{2i-2}\left(M, W\right)$ and each other (unless the FCR algorithm has converged). Together with eqn (S56)–(S57) this means that $M^3 p^{(i-1)}$ and $M^3 q^{(i-1)}$ have non-zero projections onto $S^{(i)}$. Since $Mp^{(i)}$ and $Mq^{(i)}$ form a basis for $S^{(i)}$ (eqn (S54)), we can construct the vector $Mq^{(i)}$ by first constructing a linear combination of $M^3 p^{(i-1)}$ and $M^3 q^{(i-1)}$ that is orthogonal to $Mp^{(i)}$, and then subtracting all projections onto the prior search spaces $S^{(j\leq i)}$ (which are spanned by $Mp^{(j\leq i)}$ and $Mq^{(j\leq i)}$). By eqn (S64),

$$\left\langle M^3 \left(p, q\right)^{(i-1)} \middle| M\left(p, q\right)^{(h<i-2)} \right\rangle = \left\langle \left(p, q\right)^{(i-1)} \middle| M^4 \left(p, q\right)^{(h<i-2)} \right\rangle = 0 \tag{S77}$$

Therefore, projections onto $S^{(h<i-2)}$ are automatically zero in exact arithmetic. The following ordered sequence of assignment operations gives $q^{(i)}$ with these required properties. Assigning

$$\kappa_1^{(i)} = -\frac{\left\langle M^2p^{(i)} \middle| M^2q^{(i-1)} \right\rangle}{\sqrt{\left|\left\langle M^2p^{(i)} \middle| M^2q^{(i-1)} \right\rangle\right|^2 + \left|\left\langle M^2p^{(i)} \middle| M^2p^{(i-1)} \right\rangle\right|^2}} \tag{S78}$$

$$\kappa_2^{(i)} = \frac{\left\langle M^2p^{(i)} \middle| M^2p^{(i-1)} \right\rangle}{\sqrt{\left|\left\langle M^2p^{(i)} \middle| M^2q^{(i-1)} \right\rangle\right|^2 + \left|\left\langle M^2p^{(i)} \middle| M^2p^{(i-1)} \right\rangle\right|^2}} \tag{S79}$$

$$q^{(i)} \leftarrow \kappa_1^{(i)} M^2p^{(i-1)} + \kappa_2^{(i)} M^2q^{(i-1)} \tag{S80}$$

yields a provisional $q^{(i)}$ that is M^2-conjugate to $p^{(i)}$. Assigning

$$\chi_2^{(i)} = \frac{\left\langle M^2p^{(i-2)} \middle| q^{(i)} \right\rangle}{\left\langle Mp^{(i-2)} \middle| Mp^{(i-2)} \right\rangle} \tag{S81}$$

$$\sigma_2^{(i)} = \frac{\left\langle M^2q^{(i-2)} \middle| q^{(i)} \right\rangle}{\left\langle Mq^{(i-2)} \middle| Mq^{(i-2)} \right\rangle} \tag{S82}$$

$$q^{(i)} \leftarrow q^{(i)} - \chi_2^{(i)} p^{(i-2)} - \sigma_2^{(i)} q^{(i-2)} \tag{S83}$$

yields a provisional $q^{(i)}$ that is M^2-conjugate to $p^{(i-2)}$ and $p^{(i-2)}$ in exact arithmetic. Assigning

$$\chi_1^{(i)} = \frac{\left\langle M^2p^{(i-1)} \middle| q^{(i)} \right\rangle}{\left\langle Mp^{(i-1)} \middle| Mp^{(i-1)} \right\rangle} \tag{S84}$$

$$\sigma_1^{(i)} = \frac{\left\langle M^2q^{(i-1)} \middle| q^{(i)} \right\rangle}{\left\langle Mq^{(i-1)} \middle| Mq^{(i-1)} \right\rangle} \tag{S85}$$

$$q^{(i)} \leftarrow q^{(i)} - \chi_1^{(i)} p^{(i-1)} - \sigma_1^{(i)} q^{(i-1)} \tag{S86}$$

yields a provisional $q^{(i)}$ that is M^2-conjugate to $p^{(i-1)}$ and $p^{(i-1)}$. Assigning

$$\chi_0^{(i)} = \frac{\left\langle M^2p^{(i)} \middle| q^{(i)} \right\rangle}{\left\langle Mp^{(i)} \middle| Mp^{(i)} \right\rangle} \tag{S87}$$

$$q^{(i)} \leftarrow q^{(i)} - \chi_0^{(i)} p^{(i)} \tag{S88}$$

yields non-provisional $q^{(i)}$ that is M^2-conjugate to $p^{(i)}$.

The algorithm is initialized with

$$p^{(i\le 0)} = q^{(i\le 0)} = 0 \tag{S89}$$

$$p^{(1)} = Mz^{(0)} = MW \tag{S90}$$

$$q^{(1)} = z^{(0)} - \frac{\left\langle MW \middle| M^2W \right\rangle}{\left\langle Mp^{(1)} \middle| Mp^{(1)} \right\rangle} p^{(1)} \tag{S91}$$

This choice satisfies the conjugacy condition:

$$\left\langle Mp^{(1)} \middle| Mq^{(1)} \right\rangle = \left\langle Mp^{(1)} \middle| Mz^{(0)} - \frac{\left\langle MW \middle| M^2W \right\rangle}{\left\langle Mp^{(1)} \middle| Mp^{(1)} \right\rangle} Mp^{(1)} \right\rangle$$
$$= \left\langle M^2W \middle| MW - \frac{\left\langle MW \middle| M^2W \right\rangle}{\left\langle M^2W \middle| M^2W \right\rangle} M^2W \right\rangle \quad \text{(S92)}$$
$$= \left\langle M^2W \middle| MW \right\rangle - \left\langle MW \middle| M^2W \right\rangle$$
$$= 0$$

S2.4 Vector lengths in exact arithmetic

The above relationships prove the z-search directions are orthogonal and members of the appropriate Krylov subspaces, but additional analysis is required to prove the search directions have non-zero lengths (at least until convergence). If $p^{(i)} = 0$, $Mp^{(i)} = 0$, $q^{(i)} = 0$, or $Mq^{(i)} = 0$ occur on any iteration except the last one, then the correct Krylov subspaces may not be spanned by these vectors. Note that a vector ϑ equals zero if and only if its squared length ($\left\langle \vartheta \middle| \vartheta \right\rangle$) is also zero. Also, the squared length of any vector is non-negative:

$$\left\langle \vartheta \middle| \vartheta \right\rangle \geq 0 \quad \text{(S93)}$$

Here is a proof that $p^{(i)} \neq 0$ until convergence is reached. From eqn (S53),

$$\left\langle Mz^{(i-1)} \middle| p^{(i-1)} \right\rangle = \left\langle Mz^{(i-1)} \middle| q^{(i-1)} \right\rangle = 0 \quad \text{(S94)}$$

Thus, taking the dot product of eqn (S65) with itself yields

$$\left\langle p^{(i)} \middle| p^{(i)} \right\rangle = \left\langle Mz^{(i-1)} \middle| Mz^{(i-1)} \right\rangle + \left\langle \beta^{(i)} p^{(i-1)} + \xi^{(i)} q^{(i-1)} \middle| \beta^{(i)} p^{(i-1)} + \xi^{(i)} q^{(i-1)} \right\rangle > 0 \quad \text{(S95)}$$

From eqn (S93), the two terms on the right side of eqn (S95) must be non-negative. As mentioned in Section S2.2.4 above, if $\left\langle Mz^{(i-1)} \middle| Mz^{(i-1)} \right\rangle \Rightarrow 0$ the FCR algorithm would have already exited in iteration (i-1) due to convergence. Thus, during the FCR algorithm,

$$\left\langle p^{(i)} \middle| p^{(i)} \right\rangle > 0 \quad \text{(S96)}$$

It was already proved in Section S2.2.4 above that if $\left\langle Mz^{(i-1)} \middle| Mz^{(i-1)} \right\rangle > 0$, then $\left\langle Mp^{(i)} \middle| Mp^{(i)} \right\rangle > 0$. Therefore, $Mp^{(i)} \neq 0$ during the FCR algorithm.

The condition under which $q^{(i)} = 0$ could occur is now derived. Examining eqn (S78) – (S88), $q^{(i)}$ is a linear combination of $M^2p^{(i-1)}$, $M^2q^{(i-1)}$, $p^{(i-1)}$, $q^{(i-1)}$, $p^{(i-2)}$, and $q^{(i-2)}$ that fulfills the conjugacy relations in eqn (S52). (Note that in exact arithmetic, $\chi_0^{(i)} = 0$ because eqn (S78) – (S80) already enforce $\left\langle Mp^{(i)} \middle| Mq^{(i)} \right\rangle = 0$) The condition $q^{(i)} = 0$ would imply that $\text{span}\left[M^2p^{(i-1)}, M^2q^{(i-1)}\right] \subset \text{span}\left[p^{j\leq i}, q^{j<i}\right]$. This could only occur if

$\text{span}\left[p^{j\le i}, q^{j<i}, M^2 p^{(i-1)}, M^2 q^{(i-1)}\right]$ contains fewer than 2i linearly independent vectors. Combining eqn (S51) and (S76) gives

$$\text{span}\left[p^{j\le i}, q^{j<i}, M^2 p^{(i-1)}, M^2 q^{(i-1)}\right] = K_{2i}\left(M, W\right) \tag{S97}$$

Therefore $K_{2i}\left(M, W\right)$ contains $\le$ (2i -1) linearly independent terms if $q^{(i)} = 0$. From eqn (S43), this can only mean there are $\Xi \le 2i - 1$ distinct eigenvalues having non-zero contributions to W. As derived in Sections S2.2.2 and S2.2.3 above, FCR converges in at most ceiling(Ξ/2) iterations. Thus, FCR converges in at most ceiling((2i-1)/2) = i iterations when $q^{(i)} = 0$. Therefore, $q^{(i)} = 0$ can occur only on the last iteration during which FCR converges.

The condition $Mq^{(i)} = 0$ could only occur if $q^{(i)} \in \text{kernel}\left(M\right)$. This can occur in the $q^{(i)} = 0$ case discussed in the prior paragraph (i.e., on the last iteration during which FCR converges). It may be possible for this condition to also arise during other iterations. The algorithm includes a division by zero check to handle cases where $\left\langle Mq^{(i)} \middle| Mq^{(i)} \right\rangle = 0$. When $\left\langle Mq^{(i)} \middle| Mq^{(i)} \right\rangle = 0$ the required M^2-conjugacy between a vector ϑ and $q^{(i)}$ is automatically satisfied: $\left\langle M\vartheta \middle| Mq^{(i)} = 0 \right\rangle = 0$. Therefore, the corresponding parameter $\tau^{(i)}$ (eqn (S62)), $\xi^{(i)}$ (eqn (S72)), $\kappa_1^{(i)}$ (eqn (S78)), $\kappa_2^{(i)}$ (eqn (S79)), $\sigma_1^{(i)}$ (eqn (S85)), or $\sigma_2^{(i)}$ (eqn (S82)) is set to zero when its corresponding denominator would be zero.

S2.5 Robust convergence and round-off error resistance

The Orthodir conjugate gradient algorithm can work for both positive definite and indefinite Hermitian coefficients matrix M.[3] However, it suffers from the accumulation of round-off errors. In the Orthodir algorithm, the y-search direction at each successive iteration is computed as

$$p^{(i)} = Mp^{(i-1)} - \varsigma^{(i)} p^{(i-1)} - \vartheta^{(i)} p^{(i-2)} \tag{S98}$$

where $\varsigma^{(i)}$ and $\vartheta^{(i)}$ are chosen to fulfill some chosen conjugacy condition.[3] In exact arithmetic, eqn (S98) would enforce orthogonality between $Mp^{(i)}$, $Mp^{(i-1)}$, and $Mp^{(i-2)}$. Because the choice of direction $p^{(i)}$ does not explicitly depend on the residual's value (see eqn (S98)), a buildup of round-off errors over many iterations can cause the chosen z-search direction $Mp^{(i)}$ to become uncorrelated to the residual's value. When this occurs, the Orthodir algorithm is does not operate as intended and may fail to converge.

The FCR algorithm solves this problem by making the new search direction $p^{(i)}$ depend explicitly on the residual's current value (eqn (S73)). Thus, irrespective of any round-off errors that have occurred, this search direction will never become uncoupled from the residual's value. Round-off errors can potentially cause the $q^{(i)}$ direction to stray from its precise value.

FCR explicitly enforces orthogonality between $\mathrm{Mp}^{(i)}$, $\mathrm{Mq}^{(i)}$, $\mathrm{Mp}^{(i-1)}$, and $\mathrm{Mq}^{(i-1)}$ in each iteration regardless of any round-off errors that have occurred. Eqn (S87)–(S88) ensure that

$$\left\langle \mathrm{Mq}^{(i)} \middle| \mathrm{Mp}^{(i)} \right\rangle = 0 \tag{S99}$$

even if round-off errors occurred during prior computations. With this result, eqn (S84)–(S86) and eqn (S65), (S69), and (S72) ensure that

$$\left\langle \mathrm{Mq}^{(i)} \middle| \mathrm{Mp}^{(i-1)} \right\rangle = \left\langle \mathrm{Mq}^{(i)} \middle| \mathrm{Mq}^{(i-1)} \right\rangle = 0 \tag{S100}$$

$$\left\langle \mathrm{Mp}^{(i)} \middle| \mathrm{Mp}^{(i-1)} \right\rangle = \left\langle \mathrm{Mp}^{(i)} \middle| \mathrm{Mq}^{(i-1)} \right\rangle = 0 \tag{S101}$$

even if round-off errors occurred during prior computations. M^2-conjugacy between $(\mathrm{p},\mathrm{q})^{(i)}$ and $(\mathrm{p},\mathrm{q})^{(h<i-1)}$ may be compromised by round-off errors.

Irrespective of any round-off errors that occurred during prior computations, eqn (S99), (S61), and (S62) ensure that

$$\left\langle \mathrm{Mq}^{(i)} \middle| \mathrm{z}^{(i)} \right\rangle = \left\langle \mathrm{Mp}^{(i)} \middle| \mathrm{z}^{(i)} \right\rangle = 0 \tag{S102}$$

In other words, during iteration i the conditioned residual components along z-search directions $\mathrm{Mp}^{(i)}$ and $\mathrm{Mq}^{(i)}$ are completely removed regardless of any round-off errors that occurred during prior computations.

It will now be proved $\Gamma^{(i)}$ necessarily decreases in each iteration until FCR converges, even when using fixed precision arithmetic. Rearranging eqn (S49) gives

$$\mathrm{z}^{(i-1)} = \mathrm{z}^{(i)} + \gamma^{(i)}\mathrm{Mp}^{(i)} + \tau^{(i)}\mathrm{Mq}^{(i)} \tag{S103}$$

Taking the dot product of eqn (S103) with itself gives

$$\begin{aligned}\left\langle \mathrm{z}^{(i-1)} \middle| \mathrm{z}^{(i-1)} \right\rangle &= \left\langle \mathrm{z}^{(i)} \middle| \mathrm{z}^{(i)} \right\rangle + \left|\gamma^{(i)}\right|^2 \left\langle \mathrm{Mp}^{(i)} \middle| \mathrm{Mp}^{(i)} \right\rangle + \left|\tau^{(i)}\right|^2 \left\langle \mathrm{Mq}^{(i)} \middle| \mathrm{Mq}^{(i)} \right\rangle \\ &+ \gamma^{(i)} \underbrace{\left\langle \mathrm{z}^{(i)} \middle| \mathrm{Mp}^{(i)} \right\rangle}_{0} + \tau^{(i)} \underbrace{\left\langle \mathrm{z}^{(i)} \middle| \mathrm{Mq}^{(i)} \right\rangle}_{0} + \left(\gamma^{(i)}*\right)\tau^{(i)} \underbrace{\left\langle \mathrm{Mp}^{(i)} \middle| \mathrm{Mq}^{(i)} \right\rangle}_{0} \\ &+ \left(\gamma^{(i)}*\right) \underbrace{\left\langle \mathrm{Mp}^{(i)} \middle| \mathrm{z}^{(i)} \right\rangle}_{0} + \left(\tau^{(i)}*\right) \underbrace{\left\langle \mathrm{Mq}^{(i)} \middle| \mathrm{z}^{(i)} \right\rangle}_{0} + \left(\tau^{(i)}*\right)\gamma^{(i)} \underbrace{\left\langle \mathrm{Mq}^{(i)} \middle| \mathrm{Mp}^{(i)} \right\rangle}_{0}\end{aligned} \tag{S104}$$

Hence,

$$\left\langle \mathrm{z}^{(i-1)} \middle| \mathrm{z}^{(i-1)} \right\rangle - \left\langle \mathrm{z}^{(i)} \middle| \mathrm{z}^{(i)} \right\rangle = \left|\gamma^{(i)}\right|^2 \left\langle \mathrm{Mp}^{(i)} \middle| \mathrm{Mp}^{(i)} \right\rangle + \left|\tau^{(i)}\right|^2 \left\langle \mathrm{Mq}^{(i)} \middle| \mathrm{Mq}^{(i)} \right\rangle \tag{S105}$$

Substituting eqn (S33), (S61), and (S62) into (S105) gives

$$\Gamma^{(i)} = \Gamma^{(i-1)} - \frac{\left|\left\langle \mathrm{Mp}^{(i)} \middle| \mathrm{z}^{(i-1)} \right\rangle\right|^2}{\left\langle \mathrm{Mp}^{(i)} \middle| \mathrm{Mp}^{(i)} \right\rangle} - \frac{\left|\left\langle \mathrm{Mq}^{(i)} \middle| \mathrm{z}^{(i-1)} \right\rangle\right|^2}{\left\langle \mathrm{Mq}^{(i)} \middle| \mathrm{Mq}^{(i)} \right\rangle} \tag{S106}$$

Expanding $\mathrm{p}^{(i)}$ using eqn (S73) gives

$$\left\langle Mp^{(i)} \middle| z^{(i-1)} \right\rangle = \left\langle Mz^{(i-1)} \middle| Mz^{(i-1)} \right\rangle - \left(\beta^{(i)} *\right)\underbrace{\left\langle Mp^{(i-1)} \middle| z^{(i-1)} \right\rangle}_{0} - \left(\xi^{(i)} *\right)\underbrace{\left\langle Mq^{(i-1)} \middle| z^{(i-1)} \right\rangle}_{0} \quad \text{(S107)}$$

where the quantities marked zero via eqn (S53). Substituting eqn (S107) into (S106) gives

$$\Gamma^{(i-1)} - \Gamma^{(i)} = \frac{\left\langle Mz^{(i-1)} \middle| Mz^{(i-1)} \right\rangle^2}{\left\langle Mp^{(i)} \middle| Mp^{(i)} \right\rangle} + \frac{\left|\left\langle Mq^{(i)} \middle| z^{(i-1)} \right\rangle\right|^2}{\left\langle Mq^{(i)} \middle| Mq^{(i)} \right\rangle} > 0 \quad \text{(S108)}$$

As discussed in Section S2.2.4 above, FCR is not yet converged when $\left\langle Mz^{(i-1)} \middle| Mz^{(i-1)} \right\rangle > 0$.

Inserting this into eqn (S108) gives $\Gamma^{(i-1)} - \Gamma^{(i)} > 0$ when FCR is not yet converged. Hence, the FCR algorithm always decreases the conditioned residual norm in every iteration until convergence. Because the required conjugacy conditions (eqn (S99) – (S102)) hold even for fixed precision arithmetic, eqn (S108) remains valid for fixed precision arithmetic.

S2.6 Computational algorithm

Convergence thresholds: The following three parameters control convergence thresholds. (1) FCR will exit if the maximum absolute value of every conditioned residual component is less than CR_convergence_tol. (2) FCR will exit if SQRT(<Mz | Mz>) is less than Mz_length_tolerance. The division_tolerance = (Mz_length_tolerance^2) sets the threshold for when <Mq | Mq> and related denominators are considered to be effectively zero. Mz_length_tolerance should be set to a value much smaller than CR_convergence_tol. (3) max_CR_steps specifies the maximum number of FCR iterations that can be performed.

Description of key variables to declare: (a) scalars: CR_gamma, CR_tau, CR_beta, CR_xi, CR_z_z, CR_Mz_Mz, CR_Mp_z, CR_Mq_z, CR_Mp_Mq, temp1, temp2, temp3, (b) vectors with a small number of rows and one column: CR_kappa(2 rows), CR_chi(2 rows), CR_sigma(2 rows), CR_Mp_Mp(2 rows), CR_Mq_Mq(2 rows), (c) vectors with N rows and one column: W, y, z, Mz, Mp, Mq, (d) arrays with N rows and 3 columns: p, MMp, q, MMq. If both M and W are real-valued, all of these will be declared as real variables; otherwise, they will be declared as complex variables.

Initiation: The FCR algorithm is initialized as follows:

1. Initialize y=0, z = W, p(:,:) = 0, q(:,:) = 0, MMp(:,:) = 0, MMq(:,:) = 0
2. Compute CR_z_z = <z | z> and the maximum absolute value of any z-component. If the maximum absolute value of any z-component is less than CR_convergence_tol, print the following message "The linear equation system is consistent. A nearly exact solution has been returned." and exit.
3. Compute Mz = M×z
4. Compute CR_Mz_Mz = < Mz | Mz >. If SQRT(CR_Mz_Mz) < Mz_length_tolerance, print the following message "The linear equation system is inconsistent. A least squares solution has been returned." and exit.
5. Initialize p(:,1) = Mz(:)
6. Compute Mp = M×p(:,1)

7. Compute CR_Mp_Mp(1) = <Mp | Mp> and temp1 = <p(:,1) | Mp>.
8. Initialize q(:,1) = z(:) – (temp1/ CR_Mp_Mp(1))×p(:,1)
9. Initialize Mq = Mz – (temp1/ CR_Mp_Mp(1))×Mp
10. Compute MMp(:,1) = M×Mp and MMq(:,1) = M×Mq
11. Initialize CR_Mq_Mq(:) = 1, CR_Mp_Mp(2) = 1, CR_kappa(:) = 0

<u>Iterations</u>: Each FCR iteration follows the following sequence of steps:

1. Set CR_Mq_Mq(2) = CR_Mq_Mq(1). Then compute CR_Mq_Mq(1) = <Mq | Mq>, CR_Mp_z = < Mp | z >, CR_Mq_z = < Mq | z >, and CR_Mz_Mz = <Mz | Mz>. Note that CR_Mz_Mz does not need to be used by the method, but is optionally printed along with CR_Mp_z as a consistency check to show round-off errors are suppressed since CR_Mz_Mz = CR_Mp_z in exact arithmetic.
2. Compute CR_gamma = CR_Mp_z/CR_Mp_Mp(1). IF (CR_Mq_Mq(1) > division_tolerance) THEN CR_tau = CR_Mq_z/CR_Mq_Mq(1), ELSE CR_tau = 0.
3. Compute y(:) = y(:) + CR_gamma×p(:,1) + CR_tau×q(:,1), z(:) = z(:) - CR_gamma×Mp(:) - CR_tau×Mq(:), and Mz(:) = Mz(:) - CR_gamma×MMp(:,1) - CR_tau×MMq(:,1).
4. Compute the conditioned residual norm CR_z_z = < z | z > and the maximum absolute value of any z-component. If desired, these can be printed. If the maximum absolute value of z-components is less than CR_convergence_tol, print the following message "The linear equation system is consistent. A nearly exact solution has been returned." and exit.
5. Compute CR_Mz_Mz = < Mz | Mz >. If SQRT(CR_Mz_Mz) < Mz_length_tolerance, print the following message "The linear equation system is inconsistent. A least squares solution has been returned." and exit.
6. *Optional printing of conjugacy tests*: If desired, the following quantities can be recomputed and printed: CR_Mp_z = < Mp | z >, CR_Mq_z = < Mq | z >, and CR_Mp_Mq = < Mp | Mq >. When the conjugacy conditions are satisfied, all three of these quantities should be zero. If desired, CR_Mz_Mz and CR_Mp_z can be printed; these should equal each other.
7. Shift the p and q vector related quantities to store them as prior iteration: p(:,3) = p(:,2), q(:,3) = q(:,2), MMp(:,3) = MMp(:,2), MMq(:,3) = MMq(:,2), p(:,2) = p(:,1), q(:,2) = q(:,1), MMp(:,2) = MMp(:,1), MMq(:,2) = MMq(:,1)
8. Compute temp1 = < MMp(:,2) | Mz >, temp2 = < MMq(:,2) | Mz >. Then compute CR_beta = temp1/CR_Mp_Mp(1). IF (CR_Mq_Mq(1) > division_tolerance) THEN CR_xi = temp2/CR_Mq_Mq(1), ELSE CR_xi = 0.
9. Compute p(:,1) = Mz(:) - CR_beta×p(:,2) - CR_xi×q(:,2)
10. Compute Mp = M×p(:,1)
11. Compute MMp(:,1) = M×Mp

12. Use the following sequence to compute CR_kappa. First, compute temp1 = - < MMp(:,1) | MMq(:,2) > and temp2 = < MMp(:,1) | MMp(:,2) >. Then compute temp3 = sqrt((temp1)×CONJG(temp1) + (temp2)×CONJG(temp2)). (Note: The function CONJG returns the complex conjugate.) IF (temp3 > division_tolerance) THEN CR_kappa(1) = temp1/temp3 and CR_kappa(2) = temp2/temp3, ELSE CR_kappa(:) = 0. Then compute q(:,1) = CR_kappa(1)×MMp(:,2) + CR_kappa(2)×MMq(:,2).
13. Compute temp1 = < MMp(:,3) | q(:,1) > and temp2 = < MMq(:,3) | q(:,1) >. Then compute CR_chi(2)= temp1/CR_Mp_Mp(2). IF (CR_Mq_Mq(2) > division_tolerance) THEN CR_sigma(2)= temp2/CR_Mq_Mq(2), ELSE CR_sigma(2) = 0. Then compute q(:,1) = q(:,1) – CR_chi(2)×p(:,3) – CR_sigma(2)×q(:,3).
14. Compute temp1 = < MMp(:,2) | q(:,1) > and temp2 = < MMq(:,2) | q(:,1) >. Then compute CR_chi(1)= temp1/CR_Mp_Mp(1). IF (CR_Mq_Mq(1) > division_tolerance) THEN CR_sigma(1)= temp2/CR_Mq_Mq(1), ELSE CR_sigma(1) = 0. Then compute q(:,1) = q(:,1) – CR_chi(1)×p(:,2) – CR_sigma(1)×q(:,2).
15. Set CR_Mp_Mp(2) = CR_Mp_Mp(1). Then compute temp1 = < MMp(:,1) | q(:,1) > and CR_Mp_Mp(1) = < Mp | Mp >.
16. Compute q(:,1) = q(:,1) – temp1/CR_Mp_Mp(1))×p(:,1)
17. Compute Mq = M×q(:,1)
18. Compute MMq(:,1) = M×Mq
19. IF the current FCR iteration number equals max_CR_steps, print the following message "Maximum number of FCR cycles reached. The current estimate of the solution has been returned." and exit; ELSE return to step 1 above to start the next FCR cycle.

S3. Proof the wp lookup table method yields error proportional to the interval squared

As explained in the main text, a wp lookup table is constructed on a logarithmic scale:

$$x_1 = \ln\left(\min\left(wp\_values\right)\right) - 10^{-2} \tag{S109}$$

$$x_{Num\_lookup} = \ln\left(\max\left(wp\_values\right)\right) + 10^{-2} \tag{S110}$$

$$interval = x_i - x_{i-1} = \frac{x_{Num\_lookup} - x_1}{Num\_lookup - 1} \tag{S111}$$

$$wp\_table_i = e^{x_i} \tag{S112}$$

Each atom A's polarizability α_A contributes to two adjacent array values j and (j+1), where

$$j = floor\left(\frac{\ln\left(wp\_values_A\right) - x_1}{interval} + 1\right) \tag{S113}$$

$$c_{j+1} = \frac{wp\_values_A - wp\_table_j}{wp\_table_{j+1} - wp\_table_j} \tag{S114}$$

$$c_j = 1 - c_{j+1} \tag{S115}$$

$$alpha\_table_j \leftarrow alpha\_table_j + c_j \alpha_A \tag{S116}$$

$$\mathrm{alpha\_table}_{j+1} \leftarrow \mathrm{alpha\_table}_{j+1} + c_{j+1}\alpha_A \tag{S117}$$

$$C_6^{total} = \sum_{i=1}^{\mathrm{Num\_lookup}} \sum_{k=1}^{\mathrm{Natoms}} \left[\frac{3}{2}\left(\mathrm{alpha\_table}_i\right)\left(\alpha_k\right) \frac{\mathrm{wp\_table}_i \mathrm{wp\_values}_k}{\mathrm{wp\_table}_i + \mathrm{wp\_values}_k} \right] \tag{S118}$$

Observe that

$$0 \le c_j, c_{j+1} \le 1 \tag{S119}$$

Under practical applications,

$$0.01 \le \mathrm{wp\_values}_A \le 20 \tag{S120}$$

in atomic units. Because Num_lookup=10^5 is much larger than ((ln(max(wp_values)) + 10^{-2}) – (ln(min(wp_values)) – 10^{-2})), it follows that

$$0 < \mathrm{interval} \ll 1 \tag{S121}$$

Defining

$$q = \mathrm{wp\_values}_A / \mathrm{wp\_values}_B \tag{S122}$$

gives

$$C_{6,AB} = \frac{3}{2}\alpha_A\alpha_B \frac{\mathrm{wp\_values}_A \mathrm{wp\_values}_B}{\mathrm{wp\_values}_A + \mathrm{wp\_values}_B} = \frac{3}{2}\alpha_A\alpha_B \frac{\mathrm{wp\_values}_A}{q+1} \tag{S123}$$

For k = B in the inner loop of eqn (S118), this lookup table method corresponds to the following

$$C_{6,AB} \Rightarrow \frac{3}{2}\alpha_A\alpha_B \left[c_j \frac{\mathrm{wp\_table}_j \mathrm{wp\_values}_B}{\mathrm{wp\_table}_j + \mathrm{wp\_values}_B} + c_{j+1} \frac{\mathrm{wp\_table}_{j+1} \mathrm{wp\_values}_B}{\mathrm{wp\_table}_{j+1} + \mathrm{wp\_values}_B} \right] \tag{S124}$$

Rearranging eqn (S114) gives

$$c_{j+1} = \frac{\mathrm{wp\_values}_A - \mathrm{wp\_table}_j}{\mathrm{wp\_table}_j \left(e^{\mathrm{interval}} - 1\right)} \tag{S125}$$

which further rearranges to give

$$\frac{\mathrm{wp\_values}_A}{\mathrm{wp\_table}_j} = 1 + c_{j+1}\left(e^{\mathrm{interval}} - 1\right) \tag{S126}$$

$$\frac{\mathrm{wp\_values}_A}{\mathrm{wp\_table}_{j+1}} = \frac{\mathrm{wp\_values}_A}{\mathrm{wp\_table}_j e^{\mathrm{interval}}} = \left[1 + c_{j+1}\left(e^{\mathrm{interval}} - 1\right)\right] e^{\text{-interval}} = c_{j+1} + \left(1 - c_{j+1}\right) e^{\text{-interval}} \tag{S127}$$

Expanding

$$\frac{\mathrm{wp\_table}_j \mathrm{wp\_values}_B}{\mathrm{wp\_table}_j + \mathrm{wp\_values}_B} = \frac{\mathrm{wp\_values}_A}{\frac{\mathrm{wp\_values}_A}{\mathrm{wp\_values}_B} + \frac{\mathrm{wp\_values}_A}{\mathrm{wp\_table}_j}} = \frac{\mathrm{wp\_values}_A}{q + \left(1 + c_{j+1}\left(e^{\mathrm{interval}} - 1\right)\right)} \tag{S128}$$

$$\frac{\mathrm{wp\_table}_{j+1} \mathrm{wp\_values}_B}{\mathrm{wp\_table}_{j+1} + \mathrm{wp\_values}_B} = \frac{\mathrm{wp\_values}_A}{\frac{\mathrm{wp\_values}_A}{\mathrm{wp\_values}_B} + \frac{\mathrm{wp\_values}_A}{\mathrm{wp\_table}_{j+1}}} = \frac{\mathrm{wp\_values}_A}{q + \left(c_{j+1} + \left(1 - c_{j+1}\right) e^{\text{-interval}}\right)} \tag{S129}$$

$$\frac{wp\_values_A wp\_values_B}{wp\_values_A + wp\_values_B} = \frac{wp\_values_A}{\left(\frac{wp\_values_A}{wp\_values_B}\right)+1} = \frac{wp\_values_A}{q+1} \tag{S130}$$

Hence, the relative error in computed $C_{6,AB}$ is

$$\phi = \frac{\frac{3}{2}\alpha_A\alpha_B\left[c_j \frac{wp\_table_j wp\_values_B}{wp\_table_j + wp\_values_B} + c_{j+1}\frac{wp\_table_{j+1} wp\_values_B}{wp\_table_{j+1} + wp\_values_B}\right]}{\frac{3}{2}\alpha_A\alpha_B \frac{wp\_values_A wp\_values_B}{wp\_values_A + wp\_values_B}} - 1 \tag{S131}$$

which expands as

$$\phi = \left(1-c_{j+1}\right)\frac{q+1}{q+\left(1+c_{j+1}\left(e^{interval}-1\right)\right)} + c_{j+1}\frac{q+1}{q+\left(c_{j+1}+\left(1-c_{j+1}\right)e^{-interval}\right)} - 1 \tag{S132}$$

Using Mathcad's symbolic engine, this was expanded to a Taylor series in powers of interval:

$$\phi = \frac{-\left(interval^2\right)c_{j+1}\left(1-c_{j+1}\right)q}{\left(1+q\right)^2} + \mathit{Order}\left(interval^3\right) + ... \tag{S133}$$

This formula lends itself to extremely easy magnitude analysis. Over the interval $0 \le c_{j+1} \le 1$, locating the maximum value of function $c_{j+1}\left(1-c_{j+1}\right)$ using the standard technique of setting the function's derivative to zero yields

$$0 \le c_{j+1}\left(1-c_{j+1}\right) \le 1/4 \tag{S134}$$

For $q \ge 0$, a similar analysis for the function $q/(1+q)^2$ yields

$$0 \le \frac{q}{\left(1+q\right)^2} \le \frac{1}{4} \tag{S135}$$

Inserting eqn (S134) and (S135) into (S133) yields

$$\left|\phi\right| \le \frac{interval^2}{16} \tag{S136}$$

where the third-order and higher terms can be neglected, because $0 < interval << 1$.

Because C_6^{total} is the sum of ***positive*** $C_{6,AB}$ values, the unsigned relative error (URE) of C_6^{total} is necessarily less than or equal to that of the largest URE of $C_{6,AB}$. Therefore, the URE in C_6^{total} is bounded by

$$\frac{\left|C_6^{total,calc} - C_6^{total,exact}\right|}{C_6^{total,exact}} \le \frac{interval^2}{16} \le \frac{\sim 4}{Num\_lookup^2} \tag{S137}$$

It follows from eqns (S120) and (S121) that

$$\ln\left(\frac{\max\left(wp\_values\right)}{\min\left(wp\_values\right)}\right) \le \underbrace{\left(x_{Num\_lookup} - x_1\right)}_{\left(interval\right)\left(Num\_lookup-1\right)} <\sim 8 \tag{S138}$$

Substituting eqn (S138) into the center of eqn (S137) yields the right-most inequality of eqn (S137). For Num_lookup = 10^5, a URE ≤ ~4×10^{-10} is expected.

S4. Linear-scaling algorithm for setting up the lists of interacting atom pairs

S4.1 Overview

Figure S1 is a flow diagram of the linear-scaling algorithm to set up the lists of interacting atom pairs. Data is grouped to enable fast computation by avoiding all array searches. For example, information is ordered such that arrays do not have to be searched to identify which atoms belong in each spatial region. Also, each array allocation is performed once, rather than continuously appending arrays (which would be extremely slow). This is accomplished by first performing a 'dry run' code block that executes a sequence to count up the required array size, followed by array allocation, followed by a code block that writes data to the allocated array. The four key steps to construct these lists are:

1. <u>Define basis vectors and unit cell parallelepiped</u>: A parallelepiped of non-zero volume is constructed to enclose the system's unit cell. Three basis vectors correspond to this parallelepiped's non-collinear edges. For a periodic direction, the basis vector is the corresponding periodic lattice vector. For a non-periodic direction, the basis vector is chosen to be of a non-zero length that fully encloses all the nuclear positions. Periodic basis vectors can be non-perpendicular to each other (e.g., triclinic unit cells), but each non-periodic basis vector is chosen to be perpendicular to the other basis vectors.
2. <u>Divide the unit cell parallelepiped into spatial regions</u>: This unit cell parallelepiped is divided into a whole number of spatial regions along each basis vector. A periodic direction produces an infinite number of periodic images of each region, while a non-periodic direction has only the reference image. Atoms in the reference unit cell are classified by region, and a sorted list is prepared such that atoms of the same region are adjacent in this sorted list. Because each spatial region is defined such that its volume is less than that of a sphere of dipole interaction cutoff length radius, the number of atoms in each region is always below a threshold. The code ignores regions that do not contain any atoms. Because empty regions are skipped, having a few atoms in the center of an enormous unit cell would execute quickly.
3. <u>Construct arrays listing interacting region pair images</u>: Two spatial region images interact if the minimum distance between inter-region points is ≤ the dipole interaction cutoff length. Because the spatial regions and their images are coordinate system indexed, a list of interacting region pair images is constructed without having to construct a double summation over all region pairs. Thus, even for an extremely large unit cell (e.g., containing billions of atoms) divided into many regions (e.g., millions), the list of interacting region pair images is constructed in time and memory scaling linearly with increasing unit cell size. Different regions can interact with different periodic images. For example, in an extremely large unit cell, a region near the center would interact only with nearby regions in the reference unit cell, while a region not too

far from the left edge would interact with some regions in the reference unit cell and some other region images in the left-translated unit cell. Thus, a first array is constructed listing pairs of regions having any interacting images, and a second array is prepared that lists which specific images of each particular region pair interact. Region pairs that do not interact are not included in these two arrays.

4. Construct two lists of interacting atom pairs: Because interacting atom pairs must be contained in interacting region pair images, the code identifies the interacting atom pairs by executing an outer loop over the interacting region pair images and inner loops over the atoms in these regions (along with tests for inclusion criteria). Using the list of atoms sorted by region makes this process cache access friendly. For each such atom pair, tests are performed to determine if it meets the small and large list inclusion criteria. If so, its information is added to the small and/or large lists. Because the number of interacting region pair images scales linearly with large Natoms and the number of atoms in each region is below a threshold, these small and large lists are constructed in linear-scaling computational time and memory for large Natoms.

The zip archive included in the Electronic Supplementary Information contains a folder named "construct_lists_of_interacting_atom_pairs". This folder contains example Fortran code that implements the method described here. Readers can examine this for finer coding details. This code is in the form of modules that should be called by a main program (not included). The main program must supply the required input data (e.g., number of atoms, coordinates for each atom, unit cell information, etc).

The following Sections S4.2, S4.3, S4.4, and S4.5 explain important mathematical equations and procedural details for the four key steps to construct the lists of interacting atom pairs.

Input Data

Parameters: dipole interaction cutoff length, multibody screening parameter (MCLF), distance to attenuation ratio cutoff. Unit cell properties: number of atoms, number of periodic directions, lattice vector for each periodic direction, whether to ignore periodic boundary conditions. Properties for each atom in the material: coordinates, unscreened damping radius (MCLF), wp, static unscreened polarizability

↓

Define basis vectors and unit cell parallelepiped

Define: basis vectors and origin. Compute: reciprocal basis vectors, volume of unit cell parallelepiped, coordinates and reciprocal coordinates of atoms in the reference unit cell

↓

Divide the unit cell parallelepiped into spatial regions

Define: the whole number of spatial regions along each basis vector

Group data: sort atoms in the reference unit cell according to spatial region

Indicate which spatial regions contain no atoms. (These regions are skipped in all subsequent analysis.)

↓

Construct arrays listing interacting region pair images

Compute: the sum limit along each basis vector, and the largest body diagonal of one spatial region. Construct using data grouping: one array listing the spatial region pairs having any interacting images, and another array listing which images interact for each particular interacting region pair

↓

Construct two lists of interacting atom pairs

Both the small and large lists contain only translation symmetry unique atom pairs. The small list contains 'overlapping atom images' as determined by the distance to attenuation ratio being less than the distance to attenuation ratio cutoff. For each entry in the small list, the first atom resides the in the reference unit cell, while the second atom may or may not reside in the reference unit cell. The large list contains atom pairs for which any images are within the dipole interaction cutoff length. The large list stores only sums collected over all periodic images for an atom pair; no individual image data is stored in the large list.

Figure S1: Flow diagram for setting up the lists of interacting atom pairs. The inputs followed by (MCLF) are needed only for the MCLF method and not the TS-SCS method.

S4.2 Define basis vectors and unit cell parallelepiped

A parallelepiped of non-zero volume is constructed to enclose the system's unit cell. Three basis vectors $\left(\vec{v}^{(A)}, \vec{v}^{(B)}, \vec{v}^{(C)}\right)$ correspond to this parallelepiped's non-collinear edges. For a periodic direction, the basis vector is the corresponding periodic lattice vector. For a non-periodic direction, the basis vector is chosen to be of a non-zero length that fully encloses all the nuclear positions. Periodic basis vectors can be non-perpendicular to each other (e.g., triclinic unit cells), but each non-periodic basis vector is chosen to be perpendicular to the other basis vectors. Following is a brief description of how the basis vectors and origin are chosen for different numbers of periodic boundary conditions:

0. No periodic boundary conditions: The minimum and maximum values of atomic XYZ coordinates are computed in atomic units (bohrs). The three basis vectors are chosen as: basis_vector_a = max_X – min_X + 1, basis_vector_b = max_Y – min_Y + 1, and basis_vector_c = max_Z – min_Z + 1. (This definition ensures each basis vector has a length ≥ 1 even for single atoms, linear molecules, and planar molecules.) The origin is set to origin = ((min_X - ½), (min_Y - ½), (min_Z - ½)) to ensure the reciprocal coordinates are > 0 even in the presence of rounding errors.
1. One periodic boundary condition: One basis vector is set equal to the periodic lattice vector. The two other basis vectors are defined to be perpendicular to this lattice vector and to each other. Two pre-basis vectors of unit lengths are constructed parallel to the two non-periodic directions. For each of the two non-periodic basis vectors, the following process is performed separately to determine its length: *A loop over atoms is performed. In each iteration of this loop, the dot product is constructed from the current atom's coordinates and the corresponding pre-basis vector. The minimum and maximum dot products across the set of all atoms is computed. The length of this basis vector (in atomic units) is then set to the max_dot_product – min_dot_product + 1.* Then the origin is defined such that the minimum DOT_PRODUCT((coords(atom1,:) – origin), nonperiodic_basis_vector_1) = ½ and minimum DOT_PRODUCT((coords(atom1,:) – origin), nonperiodic_basis_vector_2) = ½, where the minimum is computed over the set of all atoms.
2. Two periodic boundary conditions: Two basis vectors are set equal to the periodic lattice vectors. The third basis vector is defined to be parallel to the cross product of the two periodic lattice vectors. A pre-basis vector of unit length is formed along this direction. Then, a loop over atoms is performed. In each iteration of this loop, the dot product is constructed from the current atom's coordinates and this pre-basis vector. The minimum and maximum dot products across the set of all atoms is computed. The length of this basis vector (in atomic units) is then set to the max_dot_product – min_dot_product + 1. Then the origin is defined such that the minimum DOT_PRODUCT(((coords(atom1,:) – origin), nonperiodic_basis_vector) = ½, where the minimum is computed over the set of all atoms.

3. <u>Three periodic boundary conditions</u>: The basis vectors are set equal to the periodic lattice vectors. The origin is set to (0,0,0).

Define the matrix of basis vectors as

$$\text{basis\_vectors} = \begin{pmatrix} v_1^{(A)} & v_2^{(A)} & v_3^{(A)} \\ v_1^{(B)} & v_2^{(B)} & v_3^{(B)} \\ v_1^{(C)} & v_2^{(C)} & v_3^{(C)} \end{pmatrix} \quad \text{(S139)}$$

Then, the unit cell volume is the absolute value of the determinant:

$$\text{unit\_cell\_volume} = \text{abs}(\det(\text{basis\_vectors})) \quad \text{(S140)}$$

The reciprocal basis vectors are the transposed inverse:

$$\text{inverse\_vectors} = \left((\text{basis\_vectors})^T\right)^{-1} \quad \text{(S141)}$$

The reciprocal space coordinates for each atom1 are then given by

$$\text{inverse\_coords}(:,\text{atom1}) = \text{matmul}\left(\text{inverse\_vectors},\left(\text{coords}(:,\text{atom1}) - \text{origin}\right)\right) \quad \text{(S142)}$$

where matmul is the matrix multiplication function. Then a modulo is applied to select the image of atom A that resides within the reference unit cell:

$$\text{inverse\_coords}(:,\text{atom1}) = \text{modulo}\left(\text{inverse\_coords}(:,\text{atom1}),1.0\right) \quad \text{(S143)}$$

This ensures the reciprocal space coordinates lie within the interval ([0,1), [0,1), [0,1)).

S4.3 Divide the unit cell parallelepiped into spatial regions

First, a few geometric factors are computed from the unit cell parallelepiped:

$$\text{length\_a} = \sqrt{\text{DOT\_PRODUCT}\left(\vec{v}^{(A)},\vec{v}^{(A)}\right)} \quad \text{(S144)}$$

$$\cos\_a\_b = \frac{\text{DOT\_PRODUCT}\left(\vec{v}^{(A)},\vec{v}^{(A)}\right)}{(\text{length\_a})(\text{length\_b})} \quad \text{(S145)}$$

$$\sin\_a\_b = \sqrt{1-(\cos\_a\_b)^2} \quad \text{(S146)}$$

and so forth for the other basis vectors.

$$\text{height\_a} = \frac{\text{unit\_cell\_volume}}{\text{length\_b}\cdot\text{length\_c}\cdot\sin\_b\_c} \quad \text{(S147)}$$

is the positive projection of $\vec{v}^{(A)}$ onto the direction perpendicular to the parallelogram formed by $\vec{v}^{(B)}$ and $\vec{v}^{(C)}$. Note that (length_b · length_c · sin_b_c) is the area of this parallelogram. Analogously,

$$\text{height\_b} = \frac{\text{unit\_cell\_volume}}{\text{length\_a}\cdot\text{length\_c}\cdot\sin\_a\_c} \quad \text{(S148)}$$

$$\text{height\_c} = \frac{\text{unit\_cell\_volume}}{\text{length\_a}\cdot\text{length\_b}\cdot\sin\_a\_b} \quad \text{(S149)}$$

Then, the whole number of regions along each basis vector is calculated as:

$$\text{ref\_regions\_a} = \text{ceiling}\left(\left(\frac{3}{\text{dipole\_interaction\_cutoff\_length}}\right)\text{height\_a}\right) \tag{S150}$$

$$\text{ref\_regions\_b} = \text{ceiling}\left(\left(\left(\frac{3}{\text{dipole\_interaction\_cutoff\_length}}\right)\text{height\_b}\right)\right) \tag{S151}$$

$$\text{ref\_regions\_c} = \text{ceiling}\left(\left(\left(\frac{3}{\text{dipole\_interaction\_cutoff\_length}}\right)\text{height\_c}\right)\right) \tag{S152}$$

The ceiling function rounds up to an integer. The factor (3/dipole_interaction_cutoff_length) sets the preferred length of a region to approximately one-third the dipole interaction cutoff length. This guarantees the size of each region is always substantially smaller than the dipole interaction cutoff length while also not creating an unnecessarily large number of regions.

During a loop performed over atoms, each atom is assigned to a spatial region as labeled by three whole number indices in the interval ([1,ref_regions_a], [1,ref_regions_b], [1,ref_regions_c]). Along the first basis vector, the corresponding region index for atom1 is

$$\text{region\_a} = \text{NINT}\left(\text{ref\_regions\_a} \cdot \text{inverse\_coords}(1,\text{atom1}) + 0.5\right) \tag{S153}$$

with analogous expressions for the second and third basis vectors, where NINT rounds to the closest integer (0.5 being rounded up). If any region does not contain any atoms, this region is marked as empty and skipped in all subsequent analysis.

Finally, data grouping is performed to prepare a list of atoms and their coordinates such that all atoms from the same region are grouped together in this list. The starting and ending index values for each region in this list is computed and stored. This makes it extremely easy to perform loops over all atoms in a particular region without having to do any array searches.

S4.4 Construct arrays listing interacting region pair images

When constructing a list of representative interacting region pair images, the first region can always be chosen within the reference unit cell. Since there are no periodic translations along a non-periodic direction, then for a non-periodic direction the second region in the interacting region pair image also always lies within the reference unit cell. Along a periodic direction, the second region in the interacting region pair image can be a periodically translated image of some region such that this image has some points located closer than dipole_interaction_cutoff_length of some points in the first region. Therefore, periodicsumlimitA, periodicsumlimitB, and periodicsumlimitC are defined such that only region images with integer translation indices

$$-\text{periodicsumlimitA} \le L_1 \le \text{periodicsumlimitA} \tag{S154}$$

$$-\text{periodicsumlimitB} \le L_2 \le \text{periodicsumlimitB} \tag{S155}$$

$$-\text{periodicsumlimitC} \le L_3 \le \text{periodicsumlimitC} \tag{S156}$$

need to be tested when setting up the list of interacting region pair images. For a non-periodic direction, the corresponding periodic sum limit is obviously set to zero. If ignore_PBC = true, then

periodic boundary conditions are ignored and all periodic sum limits are set to zero. For a periodic direction, the corresponding periodic sum limit is set to the corresponding one of the following:

$$\text{periodicsumlimitA} = \text{ceiling}\left(\frac{\text{dipole\_interaction\_cutoff\_length}}{\text{height\_a}}\right) \tag{S157}$$

$$\text{periodicsumlimitB} = \text{ceiling}\left(\frac{\text{dipole\_interaction\_cutoff\_length}}{\text{height\_b}}\right) \tag{S158}$$

$$\text{periodicsumlimitC} = \text{ceiling}\left(\frac{\text{dipole\_interaction\_cutoff\_length}}{\text{height\_c}}\right) \tag{S159}$$

The longest body diagonal (aka largest_body_diag) of one spatial region is computed by finding the maximum real-space distance corresponding to a change of ((ref_regions_a)$^{-1}$, ±(ref_regions_b)$^{-1}$, ±'(ref_regions_c)$^{-1}$) in reciprocal space coordinates. The real-space distance along these diagonals is:

$$\text{distance} = \sqrt{\begin{aligned}&\left(\frac{v_1^{(A)}}{\text{ref\_regions\_a}} \pm \frac{v_1^{(B)}}{\text{ref\_regions\_b}} \pm' \frac{v_1^{(C)}}{\text{ref\_regions\_c}}\right)^2 \\ &+\left(\frac{v_2^{(A)}}{\text{ref\_regions\_a}} \pm \frac{v_2^{(B)}}{\text{ref\_regions\_b}} \pm' \frac{v_2^{(C)}}{\text{ref\_regions\_c}}\right)^2 \\ &+\left(\frac{v_3^{(A)}}{\text{ref\_regions\_a}} \pm \frac{v_3^{(B)}}{\text{ref\_regions\_a}} \pm' \frac{v_3^{(C)}}{\text{ref\_regions\_c}}\right)^2\end{aligned}} \tag{S160}$$

All four (±, ±') combinations are computed and the maximum real-space distance among these four combinations is chosen.

The condition for two region pair images to be included in the list of interacting region pair images is that the distance from the 'lower left' corner of the first region to the 'lower left' corner of the second region image should be ≤ (dipole_interaction_cutoff_length + largest_body_diag). Including the largest_body_diag ensures that all points in the first region are farther away than dipole_interaction_cutoff_length from all points in the second region image. This list is assembled in linear-scaling computational time by using the following computational efficiencies. First, only translation indices satisfying eqn (S154) – (S156) need to be considered for the second region image. Second, according to eqn (S150) – (S152) the whole number of regions along each basis vector in the reference unit cell is chosen such that the region widths (along directions parallel to each basis vector) are as large as feasible without being greater than one-third of the dipole_interaction_cutoff_length. This means a line segment of dipole_interaction_cutoff_length placed parallel to a basis vector direction could completely cross at most four regions and partially cross at most five regions. Let each region in the reference unit cell be identified by whole number triplets in the interval ([1, ref_regions_a], [1, ref_regions_b], [1, ref_regions_c]). Then let (region_A, region_B, region_C) be the region indices identifying a first region in the unit cell and (i, j, k) be the region indices identifying a second region in the reference unit cell. Then, the second region could potentially have one (or

more) images that interact with the first region if and only if all of the following conditions are satisfied. If periodicsumlimitA = 0, then

$$\max\left(\left(\text{region\_A}-5\right),1\right)\le \text{i}\le \min\left(\left(\text{region\_A}+5\right),\text{ref\_regions\_a}\right) \quad \text{(S161)}$$

else

$$\text{i}\in\left\{\text{modulo}\left(\text{region\_A}-\text{Б},\text{ref\_regions\_a}\right)\right\} \text{ for } -5\le \text{Б}\le 5 \quad \text{(S162)}$$

If periodicsumlimitB = 0, then

$$\max\left(\left(\text{region\_B}-5\right),1\right)\le \text{j}\le \min\left(\left(\text{region\_B}+5\right),\text{ref\_regions\_b}\right) \quad \text{(S163)}$$

else

$$\text{j}\in\left\{\text{modulo}\left(\text{region\_B}-\text{Б},\text{ref\_regions\_b}\right)\right\} \text{ for } -5\le \text{Б}\le 5 \quad \text{(S164)}$$

If periodicsumlimitC = 0, then

$$\max\left(\left(\text{region\_C}-5\right),1\right)\le \text{k}\le \min\left(\left(\text{region\_C}+5\right),\text{ref\_regions\_c}\right) \quad \text{(S165)}$$

else

$$\text{k}\in\left\{\text{modulo}\left(\text{region\_C}-\text{Б},\text{ref\_regions\_c}\right)\right\} \text{ for } -5\le \text{Б}\le 5 \quad \text{(S166)}$$

Loops over the above possibilities are performed and then a region pair image is added to the list if and only if the computed distance from the 'lower left' corner of the first region to the 'lower left' corner of the second region image is ≤ (dipole_interaction_cutoff_length + largest_body_diag). A first array is constructed listing pairs of regions having any interacting images, and a second array is prepared that lists which specific images of each particular region pair interact. The second array is constructed such that all images for a particular interacting region pair are grouped together. Region pairs that do not interact are not included in these arrays.

S4.5 Construct two lists of interacting atom pairs

To construct the 'small' and 'large' lists of interacting atom pairs, the outermost loop runs over atoms in the reference unit cell as taken from the array containing atoms grouped by region. For each such atom, the region to which it belongs and the atom's coordinates are loaded from the array. A second loop, which is nested inside the first loop, runs over all second regions that have at least one image interacting with the first region (as taken from the arrays constructed in Section S4.4 above). The third loop, which is nested inside the second loop, runs over all atoms in this second region. The coordinates of the second atom are loaded here. The fourth loop, which is nested inside the third loop, runs over all potentially interacting images for the chosen region pair (as taken from the arrays constructed in Section S4.4 above). Inside this fourth loop, the distance between the two atoms is computed. If this distance is larger than dipole_interaction_cutoff_length, then the program cycles to the next loop iteration. Otherwise, it checks whether the atom pair is translationally symmetry unique. If so, the atom pair is marked for inclusion in the 'large' list; otherwise, the program cycles to the next loop iteration. Then, the distance_to_attenuation_ratio is computed for the atom pair. If distance_to_attenuation_ratio ≤ distance_to_attenuation_ratio_cutoff, then the pair is marked for inclusion in the 'small' list. Then the appropriate data is written to the 'small' and/or 'large' lists if the atom pair was marked

for inclusion in those lists, respectively. (The 'large' list accumulates sums over the interacting images for a particular pair of atoms in the reference unit cell. On the other hand, the 'small' list stores separate data for individual interacting atom pair images.) Then the program proceeds to the next loop iteration. This process is continued until all iterations are complete in the four nested loops.

References:

1. E. W. Weisstein, Positive Definite Matrix, MathWorld–A Wolfram Web Resource, http://mathworld.wolfram.com/PositiveDefiniteMatrix.html, (accessed February 2019).
2. O. Axelsson and G. Lindskog, On the rate of convergence of the preconditioned conjugate-gradient method, *Numer. Math.*, 1986, **48**, 499-523. doi:10.1007/BF01389448
3. S. F. Ashby, T. A. Manteuffel and P. E. Saylor, A taxonomy for conjugate-gradient methods, *Siam J. Numer. Anal.*, 1990, **27**, 1542-1568. doi:10.1137/0727091
